# Supplementary material for: Virescenosides from the Holothurian-Associated Fungus Acremonium striatisporum Kmm 4401
Source: Mar Drugs. 2019 Oct 29;17(11):616. doi: 10.3390/md17110616 (PMC6891331; doi:10.3390/md17110616)
Supplement: Supplementary file 1 [file marinedrugs-17-00616-s001.pdf]

SUPPLEMENTARY MATERIAL

Virescenosides from the holothurian-associated fungus *Acremonium striatisporum* KMM 4401

Olesya I. Zhuravleva [1,2], Alexandr S. Antonov [1], Galina K. Oleinikova [1], Yuliya V Khudyakova [1], Roman S. Popov [1], Vladimir A. Denisenko [1], Evgeny A. Pislyagin [1], Ekaterina A. Chingizova [1], Shamil Sh. Afiyatullov [1,*]

[1] G.B. Elyakov Pacific Institute of Bioorganic Chemistry, Far Eastern Branch of the Russian Academy of Sciences, Prospect 100-letiya Vladivostoka, 159, Vladivostok 690022, Russia; zhuravleva.oi@dvfu.ru (O.I.Z.); pibocfebras@gmail.com (A.S.A.); oleingk@mail.ru (G.K.O.); 161070@rambler.ru (Y.V.K.); prs_90@mail.ru (R.S.P.); vladenis@pidoc.dvo.ru (V.A.D.); pislyagin@hotmail.com (E.A.P.); martyyas@mail.ru (E.A.C.)

[2] School of Natural Science, Far Eastern Federal University, Sukhanova St., 8, Vladivostok 690000, Russia

***** Correspondence: afiyat@piboc.dvo.ru; Tel.: +7-423-231-1168

Virescenosides from the holothurian-associated fungus *Acremonium striatisporum* KMM 4401

Ten new diterpene glycosides virescenosides Z_9-Z_{18} (**1–10**) together with three known analogues (**11–13**) and aglycon of virescenoside A (**14**) were isolated from the marine-derived fungus *Acremonium striatisporum* KMM 4401. These compounds were obtained by cultivating fungus on wort agar medium with the addition of potassium bromide. Structures of the isolated metabolites were established based on spectroscopic methods. The effects of some isolated glycosides and aglycons **15–18** on urease activity and regulation of Reactive Oxygen Species (ROS) and Nitric Oxide (NO) production in macrophages stimulated with lipopolysaccharide (LPC) were evaluated.

Keywords: *Acremonium striatisporum*, secondary metabolites, diterpene glycosides, urease activity

Content

Figure S1. Chemical structures of **11–18** ... 5
Experimental Section ... 6
*Spectral Data for compounds **15**–1**8*** ... 6
Figure S2. HPLC-MS data for subfraction II ... 7
Figure S3. ^{1}H NMR spectrum (700 MHz, Pyr-d_5) of **1** ... 8
Figure S4. ^{13}C NMR spectrum (176 MHz, Pyr-d_5) of **1** ... 9
Figure S5. COSY-45 spectrum (700 MHz, Pyr-d_5) of **1** ... 10
Figure S6. HSQC spectrum (700 MHz, Pyr-d_5) of **1** ... 11
Figure S7. HMBC spectrum (700 MHz, Pyr-d_5) of **1** ... 12
Figure S8. NOESY spectrum (700 MHz, Pyr-d_5) of **1** ... 13
Figure S9. ^{1}H NMR spectrum (700 MHz, CD_3OD) of **2** ... 14
Figure S10. ^{13}C NMR spectrum (176 MHz, CD_3OD) of **2** ... 15
Figure S11. HSQC spectrum (700 MHz, CD_3OD) of **2** ... 16
Figure S12. HMBC spectrum (700 MHz, CD_3OD) of **2** ... 17
Figure S13. NOESY spectrum (700 MHz, CD_3OD) of **2** ... 18
Figure S14. ^{1}H NMR spectrum (700 MHz, CD_3OD) of **3** ... 19
Figure S15. ^{13}C NMR spectrum (176 MHz, CD_3OD) of **3** ... 20
Figure S16. HSQC spectrum (700 MHz, CD_3OD) of **3** ... 21
Figure S17. HMBC spectrum (700 MHz, CD_3OD) of **3** ... 22
Figure S18. NOESY spectrum (700 MHz, CD_3OD) of **3** ... 23
Figure S19. ^{1}H NMR spectrum (500 MHz, CD_3OD) of **4** ... 24
Figure S20. ^{13}C NMR spectrum (125 MHz, CD_3OD) of **4** ... 25
Figure S21. HSQC spectrum (500 MHz, CD_3OD) of **4** ... 26
Figure S22. HMBC spectrum (500 MHz, CD_3OD) of **4** ... 27
Figure S23. NOESY spectrum (500 MHz, CD_3OD) of **4** ... 28
Figure S24. ^{1}H NMR spectrum (700 MHz, CD_3OD) of **5** ... 29
Figure S25. ^{13}C NMR spectrum (176 MHz, CD_3OD) of **5** ... 30
Figure S26. HSQC spectrum (700 MHz, CD_3OD) of **5** ... 31
Figure S27. HMBC spectrum (700 MHz, CD_3OD) of **5** ... 32
Figure S28. NOESY spectrum (700 MHz, CD_3OD) of **5** ... 33
Figure S29. ^{1}H NMR spectrum (500 MHz, CD_3OD) of **6** ... 34
Figure S30. ^{13}C NMR spectrum (125 MHz, CD_3OD) of **6** ... 35
Figure S31. HSQC spectrum (500 MHz, CD_3OD) of **6** ... 36
Figure S32. HMBC spectrum (500 MHz, CD_3OD) of **6** ... 37
Figure S33. NOESY spectrum (500 MHz, CD_3OD) of **6** ... 38
Figure S34. ^{1}H NMR spectrum (700 MHz, CD_3OD) of **7** ... 39
Figure S35. ^{13}C NMR spectrum (176 MHz, CD_3OD) of **7** ... 40
Figure S36. HSQC spectrum (700 MHz, CD_3OD) of **7** ... 41
Figure S37. HMBC spectrum (700 MHz, CD_3OD) of **7** ... 42
Figure S38. NOESY spectrum (700 MHz, CD_3OD) of **7** ... 43
Figure S39. ^{1}H NMR spectrum (500 MHz, CD_3OD) of **8** ... 44
Figure S40. ^{13}C NMR spectrum (125 MHz, CD_3OD) of **8** ... 45
Figure S41. HSQC spectrum (500 MHz, CD_3OD) of **8** ... 46

Figure S42. HMBC spectrum (500 MHz, CD_3OD) of **8**.....47
Figure S43. NOESY spectrum (500 MHz, CD_3OD) of **8**.....48
Figure S44. ^{1}H NMR spectrum (700 MHz, CD_3OD) of **9**.....49
Figure S45. ^{13}C NMR spectrum (176 MHz, CD_3OD) of **9**.....50
Figure S46. HSQC spectrum (700 MHz, CD_3OD) of **9**.....51
Figure S47. HMBC spectrum (700 MHz, CD_3OD) of **9**.....48
Figure S48. NOESY spectrum (700 MHz, CD_3OD) of **9**.....53
Figure S49. ^{1}H NMR spectrum (500 MHz, Pyr-d_5) of **10**.....54
Figure S50. ^{13}C NMR spectrum (125 MHz, Pyr-d_5) of **10**.....55
Figure S51. COSY-45 spectrum (500 MHz, Pyr-d_5) of **10**.....56
Figure S52. HSQC spectrum (500 MHz, Pyr-d_5) of **10**.....57
Figure S53. HMBC spectrum (500 MHz, Pyr-d_5) of **10**.....58
Figure S54. NOESY spectrum (500 MHz, Pyr-d_5) of **10**.....59
Figure S55. ^{1}H NMR spectrum (700 MHz, CD_3OD) of **11**.....60
Figure S56. ^{13}C NMR spectrum (176 MHz, CD_3OD) of **11**.....61
Figure S57. HSQC spectrum (700 MHz, CD_3OD) of **11**.....62
Figure S58. HMBC spectrum (700 MHz, CD_3OD) of **11**.....63
Figure S59. NOESY spectrum (700 MHz, CD_3OD) of **11**.....64
Figure S60. ^{1}H NMR spectrum (700 MHz, CD_3OD) of **12**.....65
Figure S61. ^{13}C NMR spectrum (176 MHz, CD_3OD) of **12**.....66
Figure S62. HSQC spectrum (700 MHz, CD_3OD) of **12**.....67
Figure S63. HMBC spectrum (700 MHz, CD_3OD) of **12**.....68
Figure S64. NOESY spectrum (700 MHz, CD_3OD) of **12**.....69
Figure S65. ^{1}H NMR spectrum (700 MHz, DMSO-d_6) of **13**.....70
Figure S66. ^{13}C NMR spectrum (176 MHz, DMSO-d_6) of **13**.....71
Figure S67. HSQC spectrum (700 MHz, DMSO-d_6) of **13**.....72
Figure S68. HMBC spectrum (700 MHz, DMSO-d_6) of **13**.....73
Figure S69. ^{1}H NMR spectrum (500 MHz, CD_3OD) of **14**.....74
Figure S70. ^{13}C NMR spectrum (125 MHz, CD_3OD) of **14**.....75
Figure S71. HSQC spectrum (500 MHz, CD_3OD) of **14**.....76
Figure S72. HMBC spectrum (500 MHz, CD_3OD) of **14**.....77
Figure S73. NOESY spectrum (500 MHz, CD_3OD) of **14**.....78

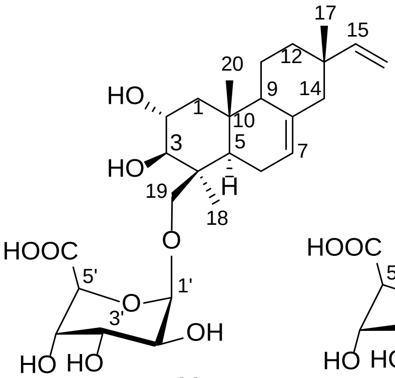

11

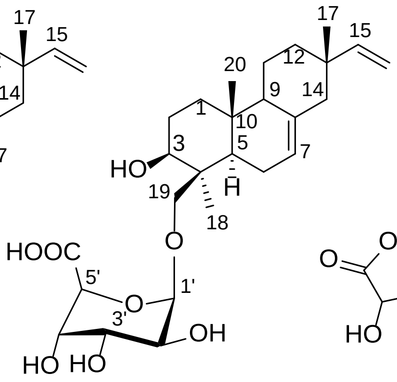

12

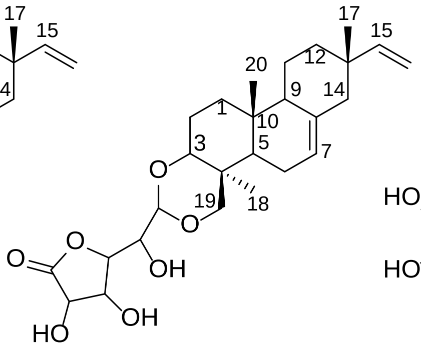

13

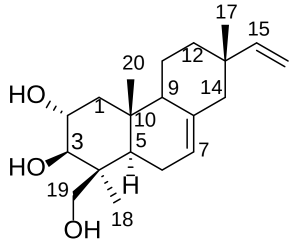

14

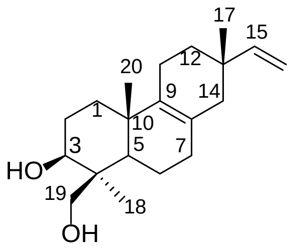

15

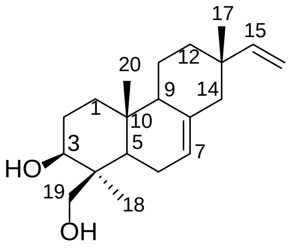

16

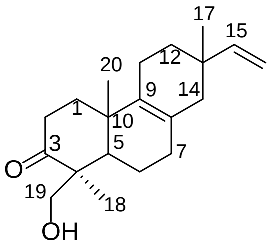

17

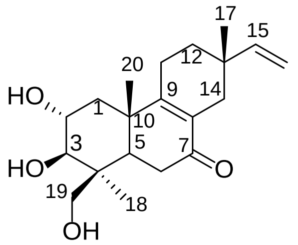

18

Figure S1. Chemical structures of **11–18**

Experimental Section

Spectral Data

Aglycon of virescenoside B ($\Delta^{8,9}$) (**15**): white powder; ^{1}H NMR (500MHz, $CDCl_3$) δ: 5.72 (1H, dd, *J* = 10.8, 17.5 Hz, H-15), 4.89 (1H, dd, *J* = 1.6, 10.8 Hz, H-16b), 4.84 (1H, dd, *J* = 1.6, 17.5 Hz, H-16a), 4.24 (1H, d, *J* = 11.1 Hz, H-19b), 3.46 (1H, dd, *J* = 4.5, 11.4 Hz, H-3), 3.34 (1H, d, *J* = 11.1 Hz, H-19a), 1.93 (2H, m, H_2-7), 1.87 (2H, m, H_2-11), 1.83 (1H, m, H-2a), 1.80 (1H, d, *J* = 16.1 Hz, H-14a), 1.77 (1H, m, H-1a), 1.75 (1H, m, H-6a), 1.74 (1H, m, H-2b), 1.71 (1H, d, *J* = 16.1 Hz, H-14b), 1.50 (1H, d, *J* = 11.1 Hz, H-12a), 1.40 (1H, dd, *J* = 4.0, 12.5 Hz, H-6b), 1.30 (1H, dd, *J* = 11.1 Hz, H-12b), 1.25 (3H, s, Me-18), 1.21 (1H, dd, *J* = 1.7, 12.7 Hz, H-5), 1.13 (1H, m, H-1b), 0.96 (3H, s, Me-17), 0.92 (3H, s, Me-20). ^{13}C NMR (125MHz, $CDCl_3$) δ: 146.1 (C-15), 136.1 (C-9), 124.7 (C-8), 110.8 (C-16), 80.8 (C-3), 64.3 (C-19), 51.8 (C-5), 42.9 (C-4), 41.8 (C-14), 37.1 (C-10), 35.1 (C-13), 34.9 (C-12), 34.5 (C-1), 32.8 (C-7), 28.2 (C-2), 28.0 (C-17), 22.3 (C-18), 21.3 (C-11), 20.1 (C-20), 18.7 (C-6); EI MS $[M]^+$ 304.

Genuine aglycon of virescenoside B ($\Delta^{7,8}$) (**16**): white powder; ^{1}H NMR (700MHz, $CDCl_3$) δ: 5.79 (1H, dd, *J* = 10.8, 17.5 Hz, H-15), 5.35 (1H, brs, H-7), 4.92 (1H, dd, *J* = 1.6, 10.8 Hz, H-16b), 4.87 (1H, dd, *J* = 1.6, 17.5 Hz, H-16a), 4.33 (1H, d, *J* = 11.1 Hz, H-19b), 3.49 (1H, dd, *J* = 4.5, 12.0 Hz, H-3), 3.48 (1H, d, *J* = 11.1 Hz, H-19a), 2.00 (1H, m, H-6a), 1.95 (1H, brd, H-14a), 1.90 (1H, dd, *J* = 2.7, 13.7 Hz, H-14b), 1.89 (1H, td, *J* = 3.6, 13.5 Hz, H-1a), 1.86 (1H, m, H-6b), 1.83 (1H, dd, *J* = 3.4, 13.6 Hz, H-2a), 1.72 (1H, qd, *J* = 3.8, 13.2 Hz, H-2b), 1.63 (1H, brs, H-9), 1.55 (1H, m, H-11a), 1.48 (1H, td, *J* = 3.0, 9.3 Hz, H-12a), 1.34 (1H, m, H-11b), 1.33 (1H, d, *J* = 9.6 Hz, H-12b), 1.27 (1H, dd, *J* = 4.1, 12.4 Hz, H-5), 1.24 (3H, s, Me-18), 1.18 (1H, dt, *J* = 3.8, 13.4 Hz, H-1b), 0.85 (3H, s, Me-17), 0.81 (3H, s, Me-20). ^{13}C NMR (175MHz, $CDCl_3$) δ: 150.2 (C-15), 135.5 (C-8), 121.2 (C-7), 109.3 (C-16), 81.3 (C-3), 64.4 (C-19), 51.9 (C-9), 51.3 (C-5), 45.9 (C-14), 42.3 (C-4), 37.8 (C-1), 36.8 (C-13), 36.1 (C-12), 35.0 (C-10), 28.0 (C-2), 23.0 (C-6), 22.5 (C-18), 21.5 (C-17), 20.4 (C-11), 16.0 (C-20); EI MS $[M]^+$ 304.

Aglycon of virescenoside C ($\Delta^{8,9}$) (**17**): white powder; ^{1}H NMR (500MHz, $CDCl_3$) δ: 5.72 (1H, dd, *J* = 10.7, 17.5 Hz, H-15), 4.91 (1H, dd, *J* = 1.6, 10.7 Hz, H-16b), 4.83 (1H, dd, *J* = 1.6, 17.5 Hz, H-16a), 4.02 (1H, d, *J* = 11.3 Hz, H-19b), 3.44 (1H, d, *J* = 11.3 Hz, H-19a), 2.58 (1H, m, H-2a), 2.45 (1H, m, H-2b), 1.98 (1H, m, H-1a), 1.96 (2H, m, H_2-7), 1.91 (1H, m, H-11), 1.90 (1H, dd, *J* = 2.3, 13.0 Hz, H-5), 1.89 (1H, m, H-11), 1.87 (1H, dd, *J* = 16.1 Hz, H-14a), 1.75 (1H, brd, *J* = 16.1 Hz, H-14b), 1.66 (1H, m, H-6a), 1.64 (1H, m, H-1b),1.52 (1H, m, H-12a), 1.45 (1H, dd, *J* = 4.0, 12.5 Hz, H-6b), 1.34 (1H, m, H-12b), 1.29 (3H, s, Me-18), 1.01 (3H, s, Me-20), 0.98 (3H, s, Me-17). ^{13}C NMR (125MHz, $CDCl_3$) δ: 221.1 (C-3), 145.8 (C-15), 134.3 (C-9), 125.8 (C-8), 110.9 (C-16), 65.8 (C-19), 52.1 (C-5), 50.7 (C-4), 41.7 (C-14), 36.9 (C-10), 35.1 (C-13), 34.7 (C-12), 34.6 (C-2), 34.4 (C-1),32.3 (C-7), 28.2 (C-17), 22.1 (C-18), 21.3 (C-11), 19.8 (C-20), 19.3 (C-6); EI MS $[M]^+$ 302.

Aglycon of virescenoside M (**18**): white powder; ^{1}H NMR (500MHz, $CDCl_3$) δ: 5.66 (1H, dd, *J* = 10.7, 17.5 Hz, H-15), 4.93 (1H, dd, *J* = 1.2, 10.7 Hz, H-16b), 4.83 (1H, dd, *J* = 1.2, 17.5 Hz, H-16a), 4.18 (1H, d, *J* = 11.2 Hz, H-19b), 3.99 (1H, ddd, *J* = 4.3, 9.6, 11.6 Hz, H-2), 3.48 (1H, dd, *J* = 1.2, 11.2 Hz, H-19a), 3.20 (1H, dd, *J* = 1.2, 9.6 Hz, H-3), 2.56 (1H, dd, *J* = 3.4, 16.9 Hz, H-6a), 2.35 (1H, d, *J* = 17.5 Hz, H-14a), 2.33 (1H, dd, *J* = 14.4, 16.9 Hz, H-6b), 2.21 (1H, dd, *J* = 4.1, 12.6 Hz, H-1a), 2.20 (1H, m, H_2-11), 2.01 (1H, dt, *J* = 2.4, 17.7 Hz, H-14b), 1.83 (1H, dd, *J* = 3.5, 14.6 Hz, H-5), 1.61 (1H, m, H-12a), 1.33 (1H, m, H-12b), 1.29 (1H, t, *J* = 11.6 Hz, H-1b), 1.28 (3H, s, Me-18), 1.14 (3H, s, Me-20), 1.01 (3H, s, Me-17). ^{13}C NMR (125MHz, $CDCl_3$) δ: 198.4 (C-7), 163.8 (C-9), 144.9 (C-15), 129.0 (C-8), 111.8 (C-16), 84.4 (C-3), 68.8 (C-2), 65.0 (C-19), 49.6 (C-5), 42.7 (C-4), 41.7 (C-1), 40.3 (C-10), 34.7 (C-6), 34.4 (C-13), 33.5 (C-12), 33.3 (C-14), 28.1 (C-17), 23.3 (C-11), 22.4 (C-18), 19.6 (C-20); EI MS $[M]^+$ 334.

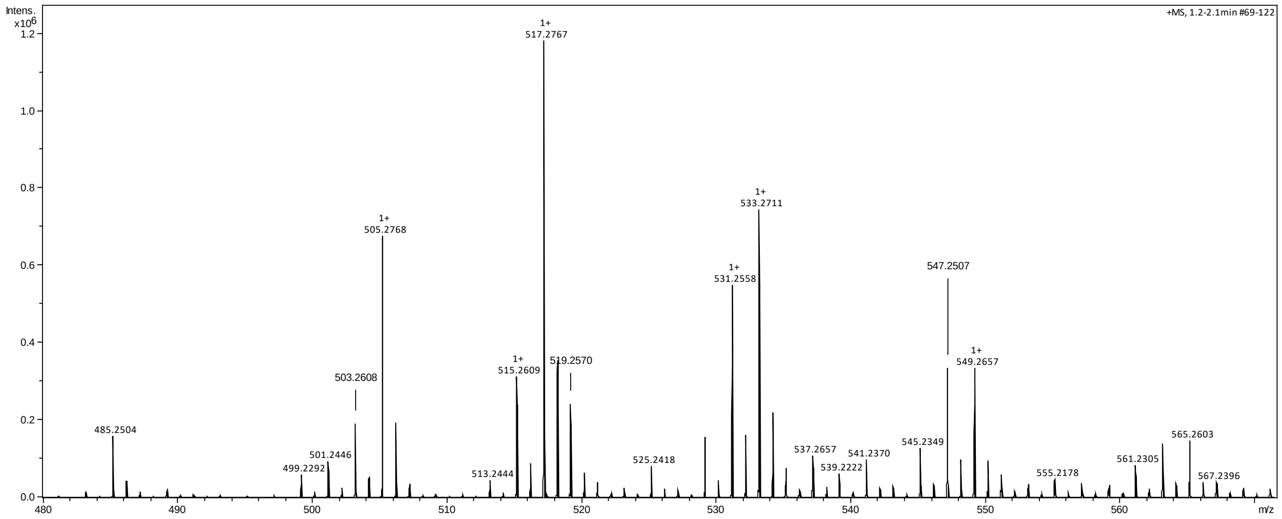

Figure S2. HPLC-MS data for subfraction II

Virescenoside Z_{12} (**4**) $[M+Na]^+$ 517.2767
Virescenoside Z_{13} (**5**) $[M+Na]^+$ 533.2711
Virescenoside Z_{14} (**6**) $[M+Na]^+$ 547.2507
Virescenoside Z_{15} (**7**) $[M+Na]^+$ 547.2507
Virescenoside Z_{16} (**8**) $[M+Na]^+$ 515.2609
Virescenoside Z_{18} (**10**) $[M+Na]^+$ 517.2767

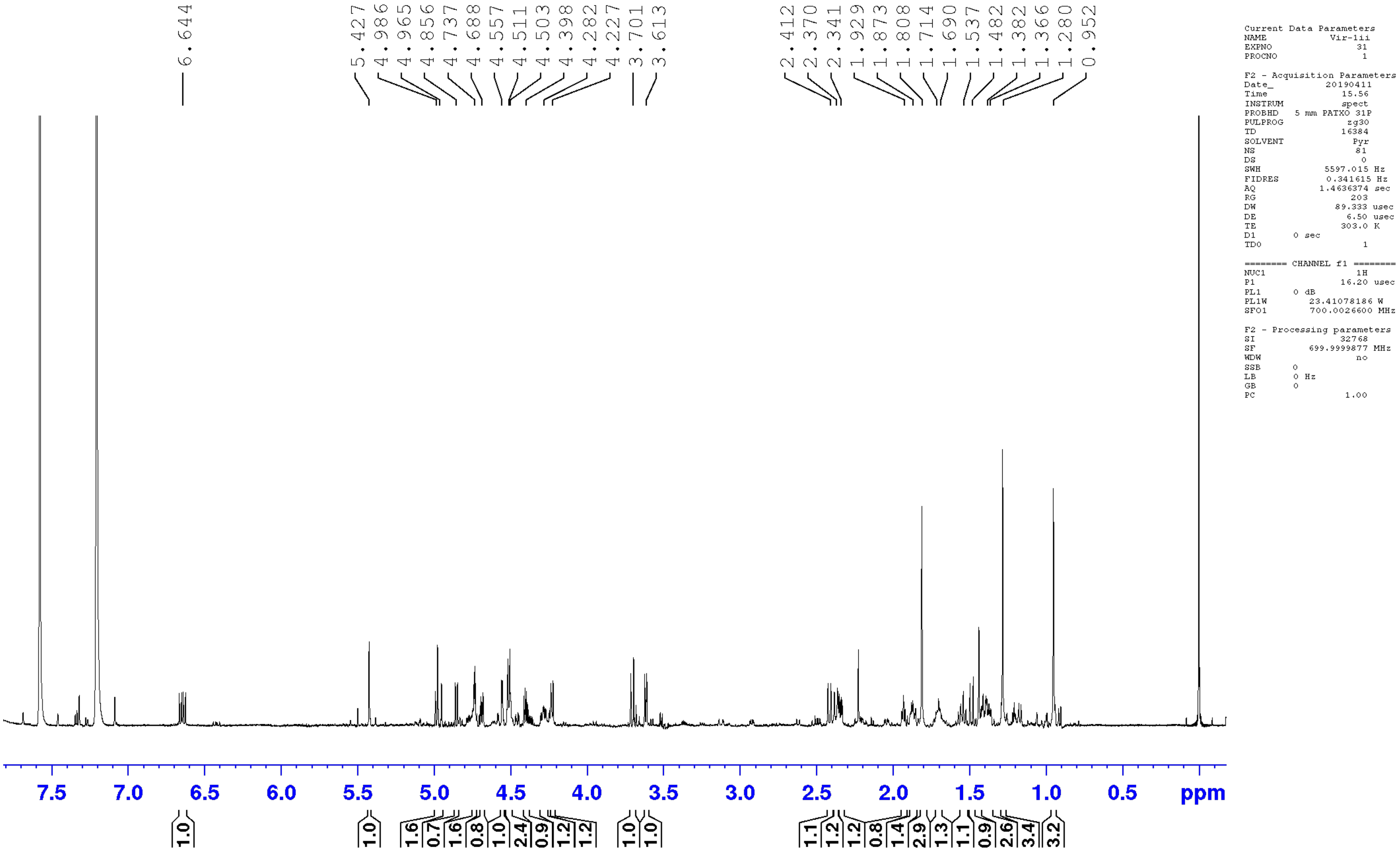

Figure S3. ^{1}H NMR spectrum (700 MHz, Pyr-d_5) of **1**

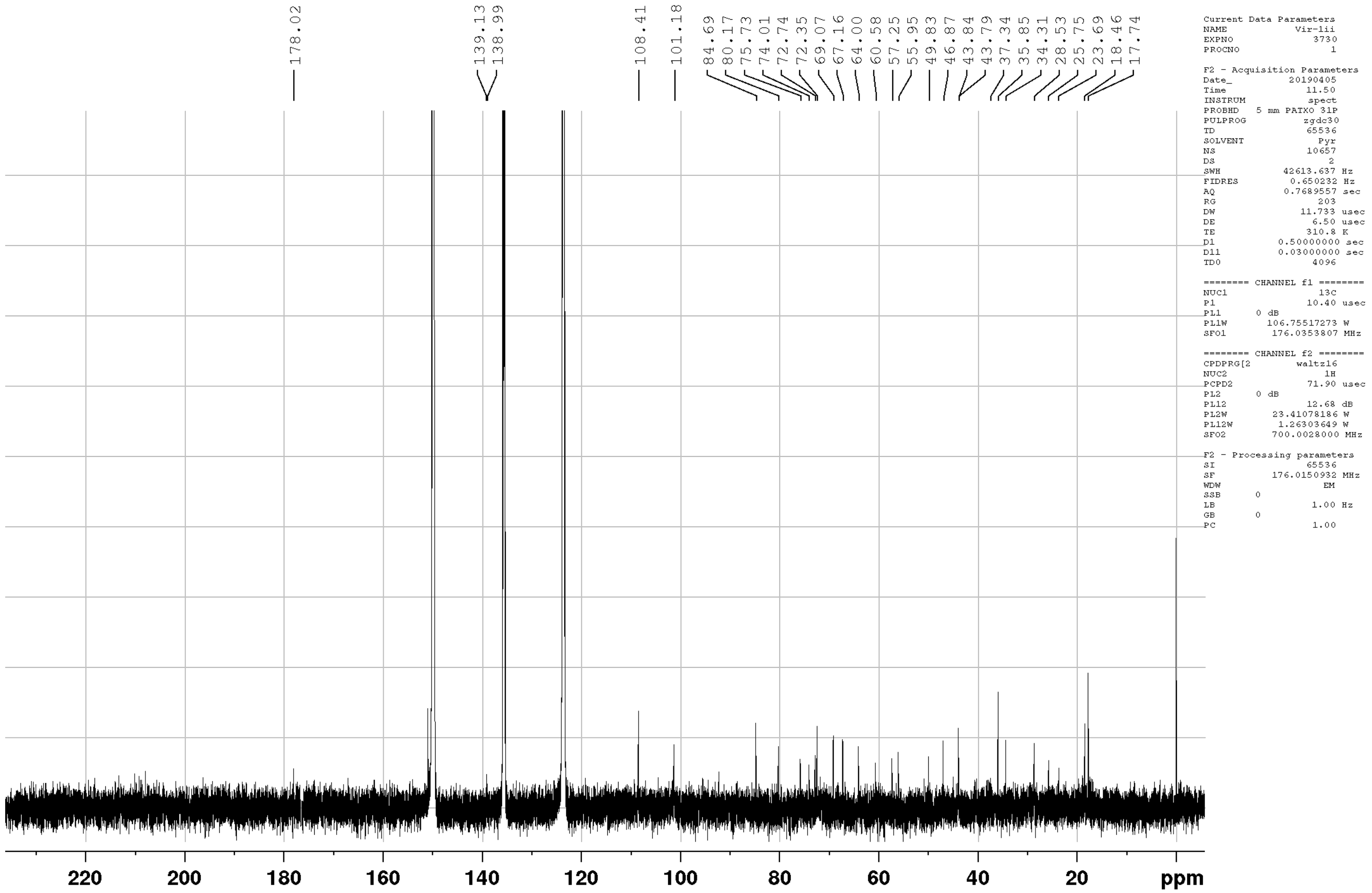

Figure S4. ^{13}C NMR spectrum (176 MHz, Pyr-d_5) of **1**

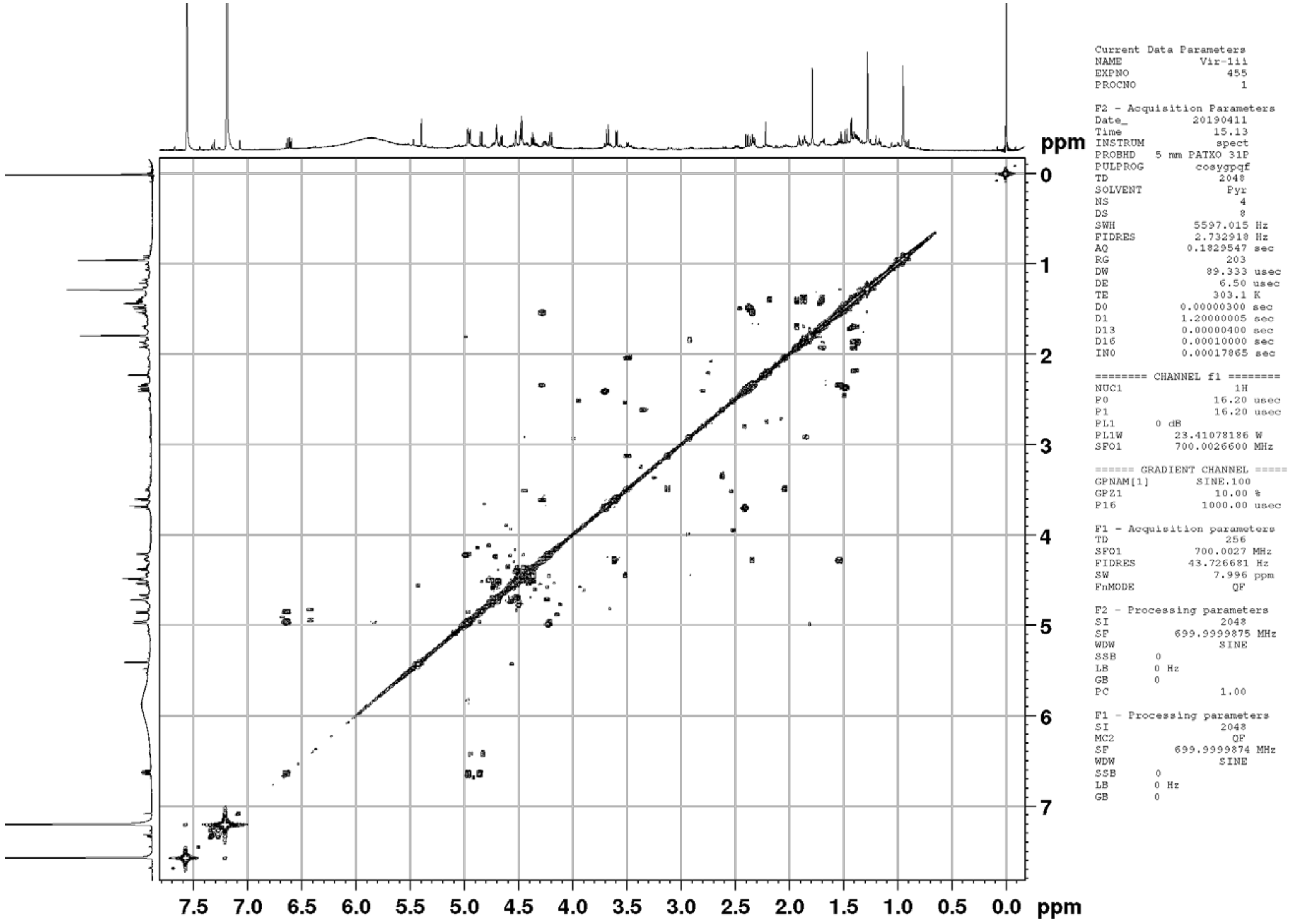

Figure S5. COSY-45 spectrum (700 MHz, Pyr-d_5) of **1**

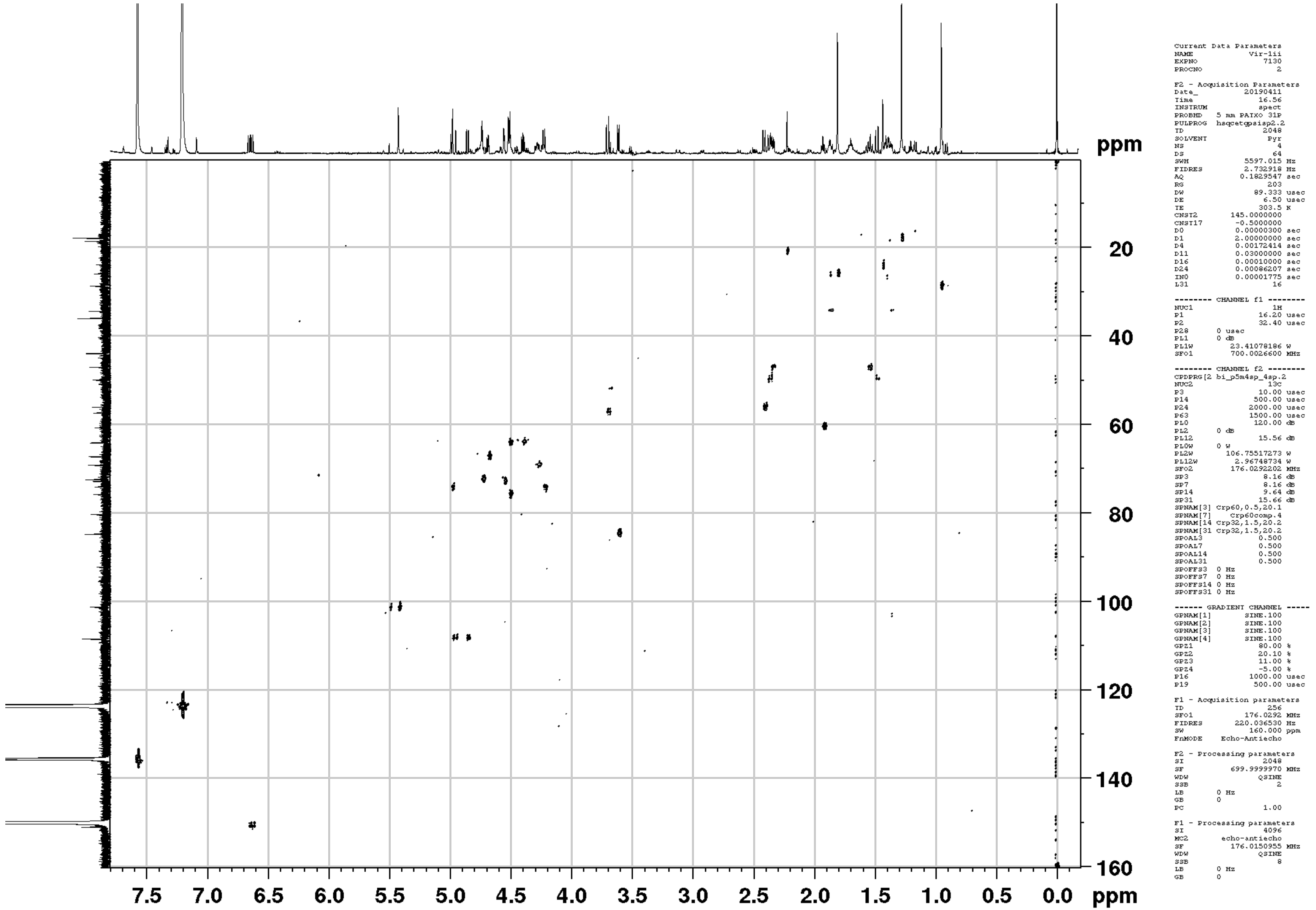

Figure S6. HSQC spectrum (700 MHz, Pyr-d_5) of **1**

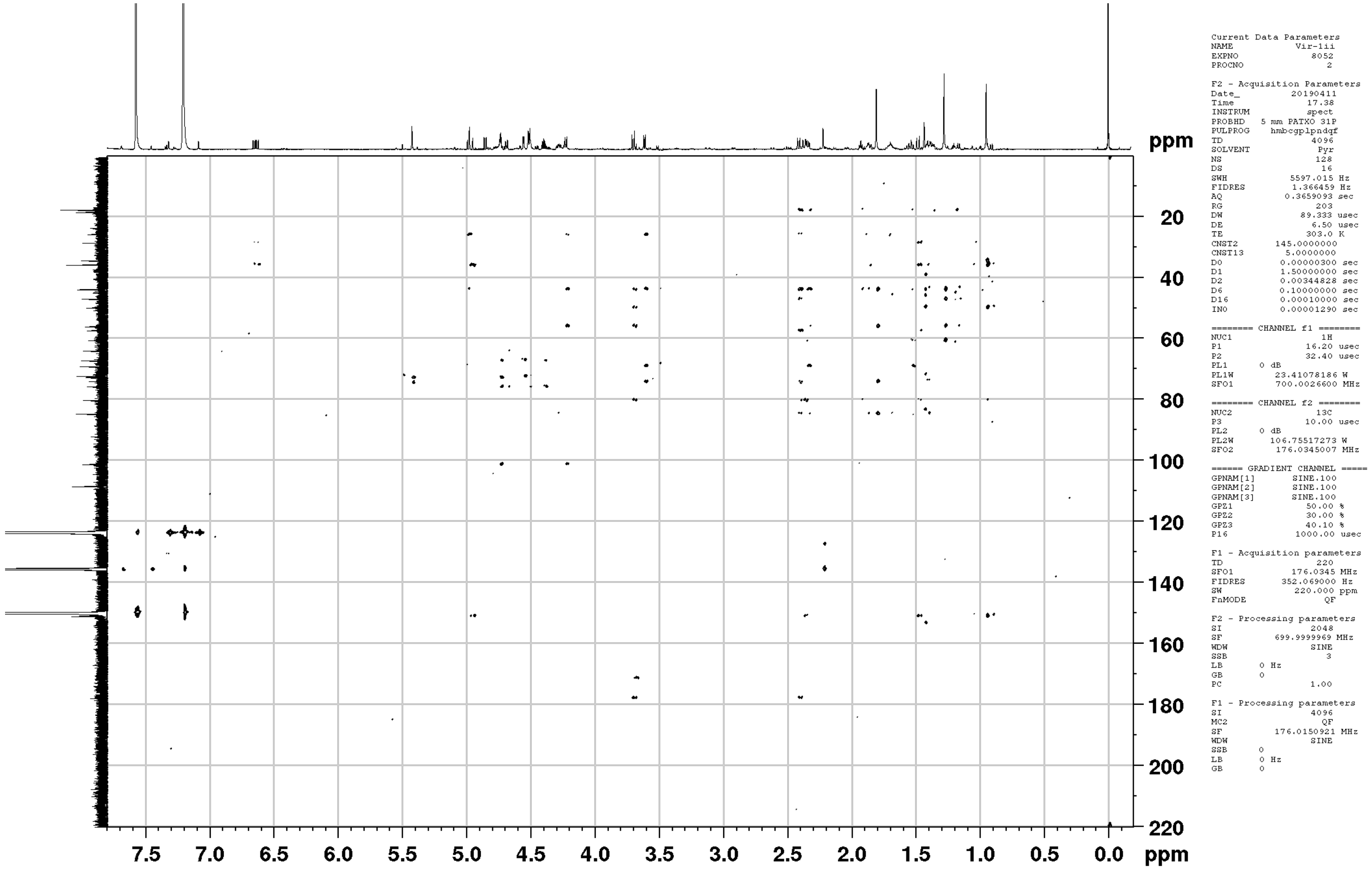

Figure S7. HMBC spectrum (700 MHz, Pyr-d_5) of **1**

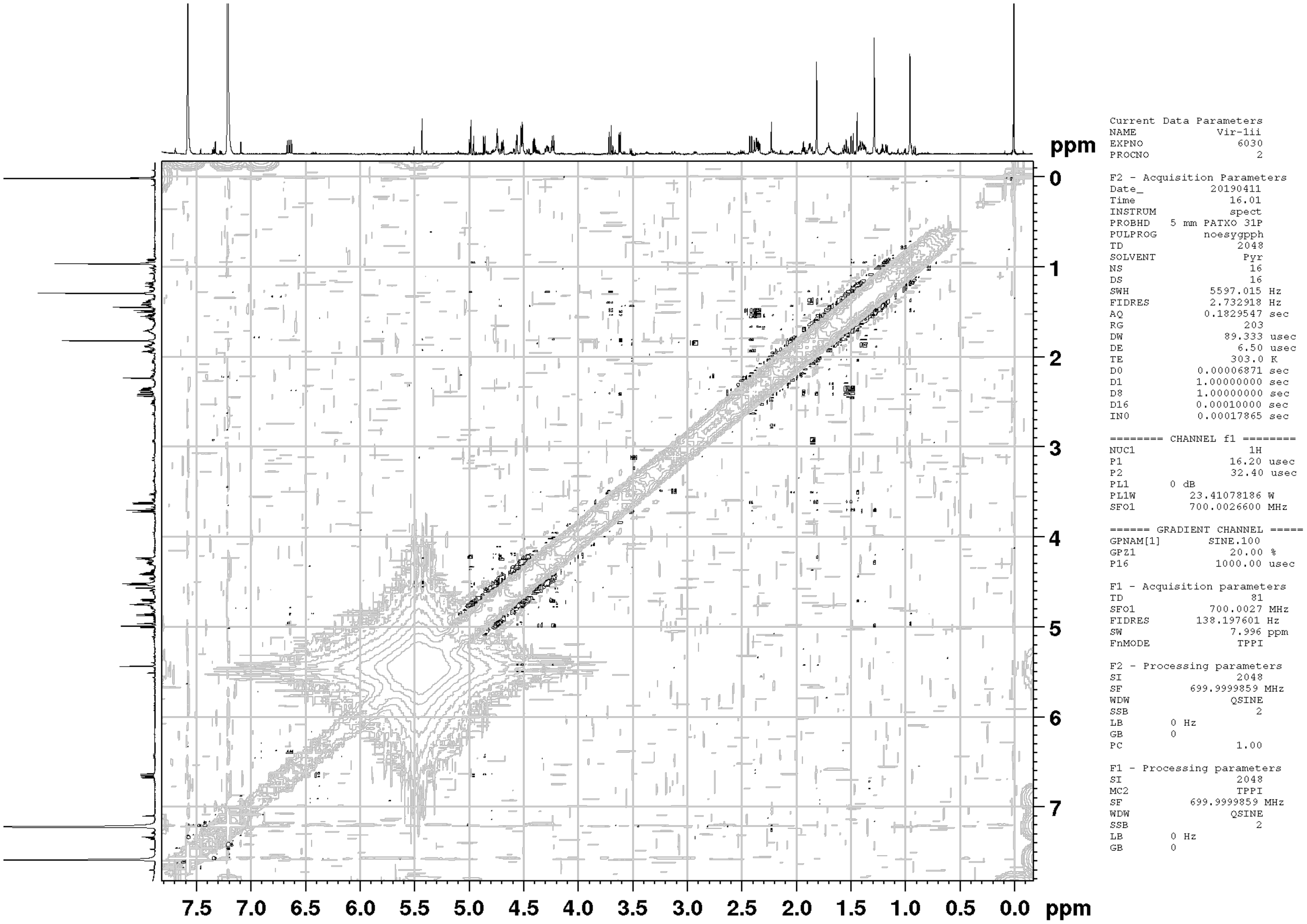

Figure S8. NOESY spectrum (700 MHz, Pyr-d_5) of **1**

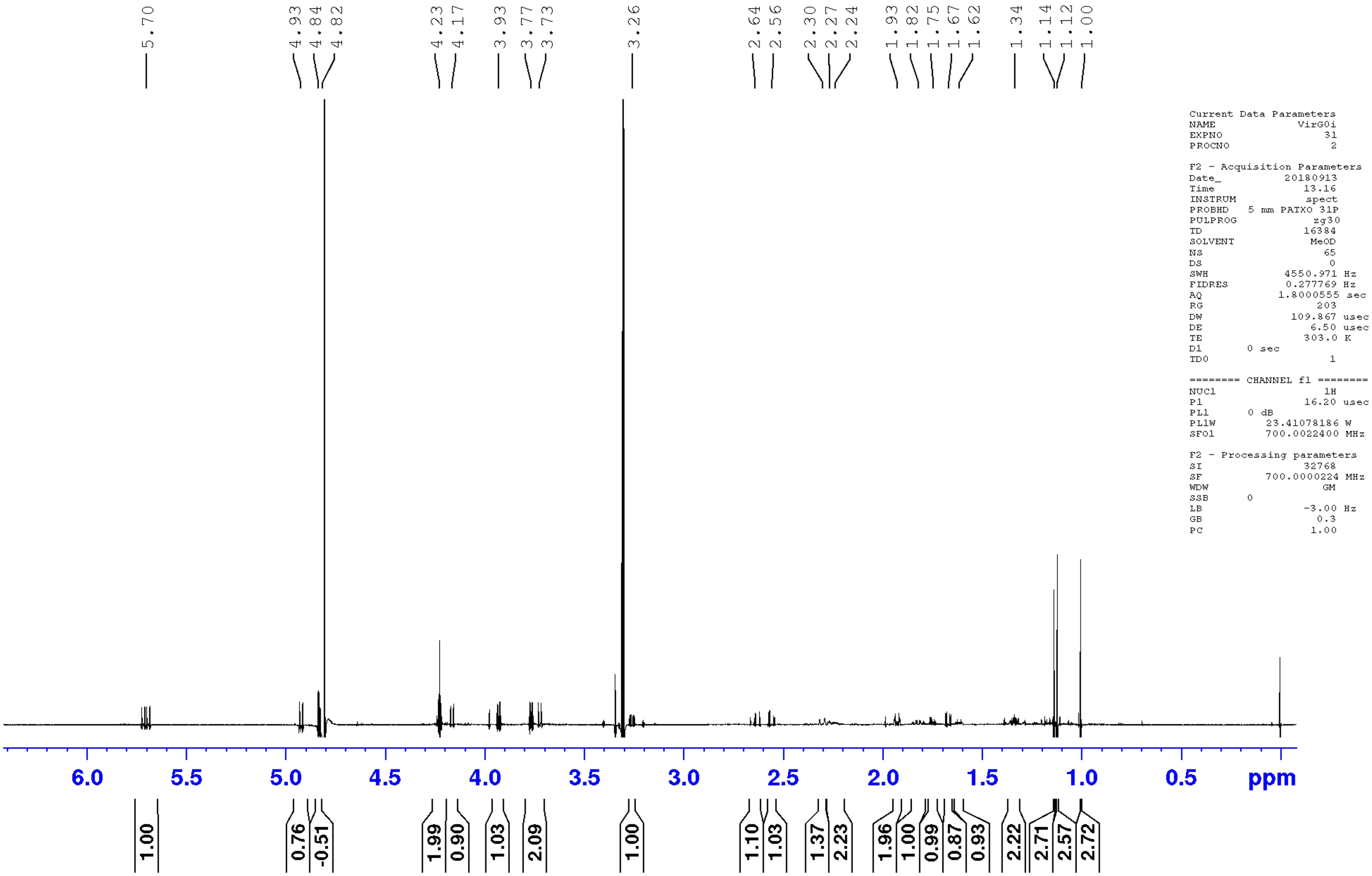

Figure S9. ^{1}H NMR spectrum (700 MHz, CD_3OD) of **2**

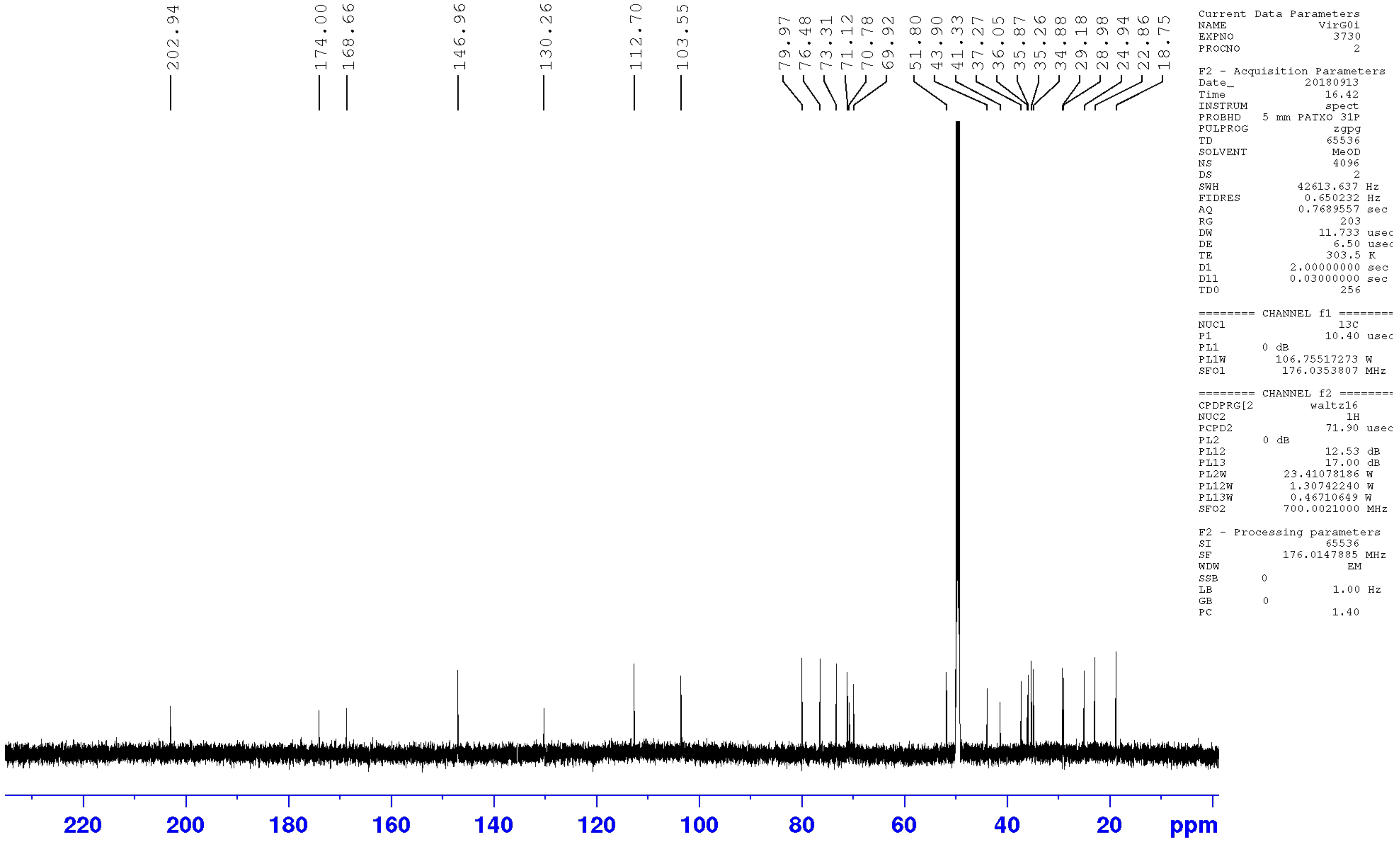

Figure S10. ^{13}C NMR spectrum (176 MHz, CD_3OD) of **2**

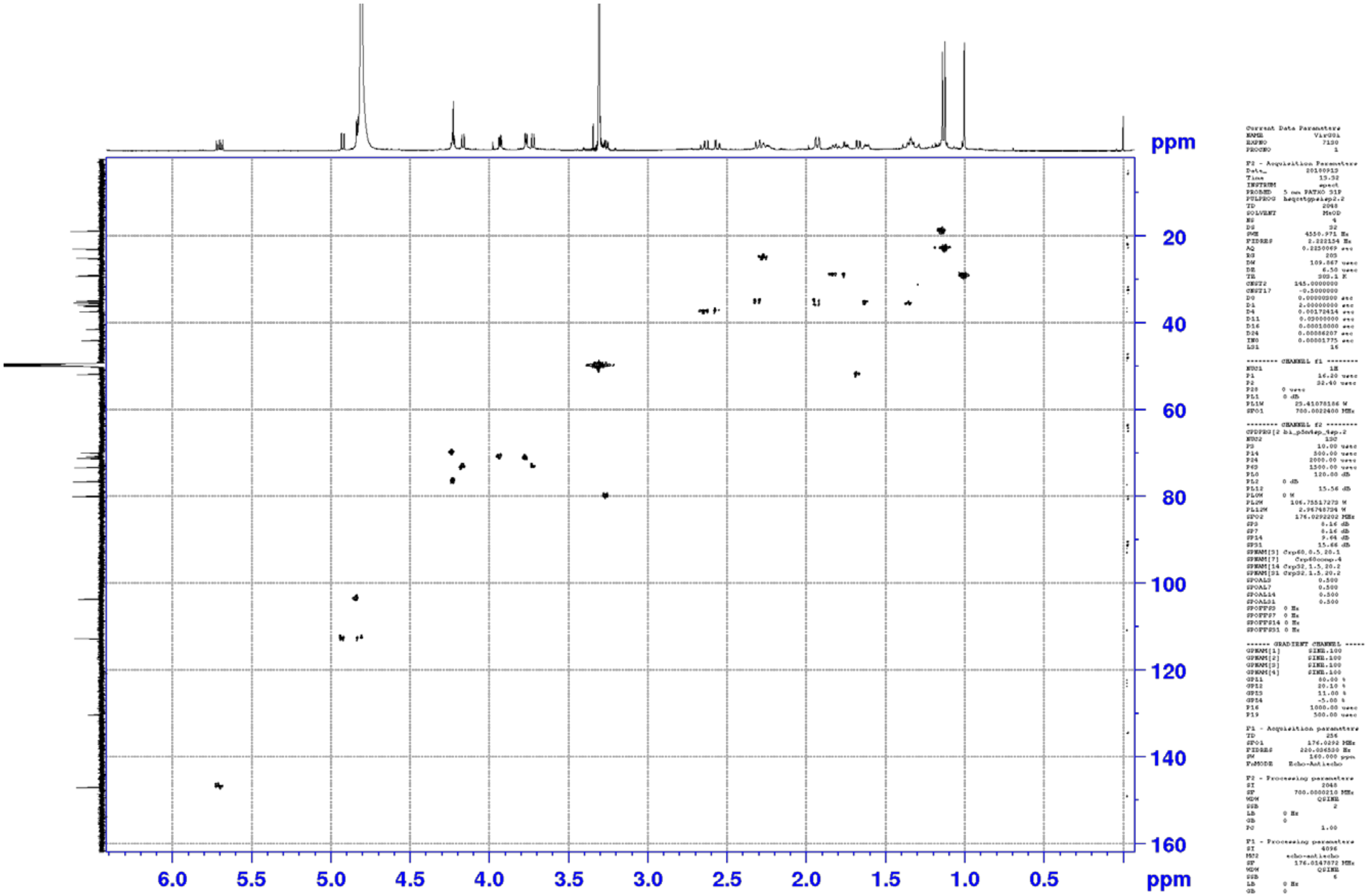

Figure S11. HSQC spectrum (700 MHz, CD_3OD) of **2**

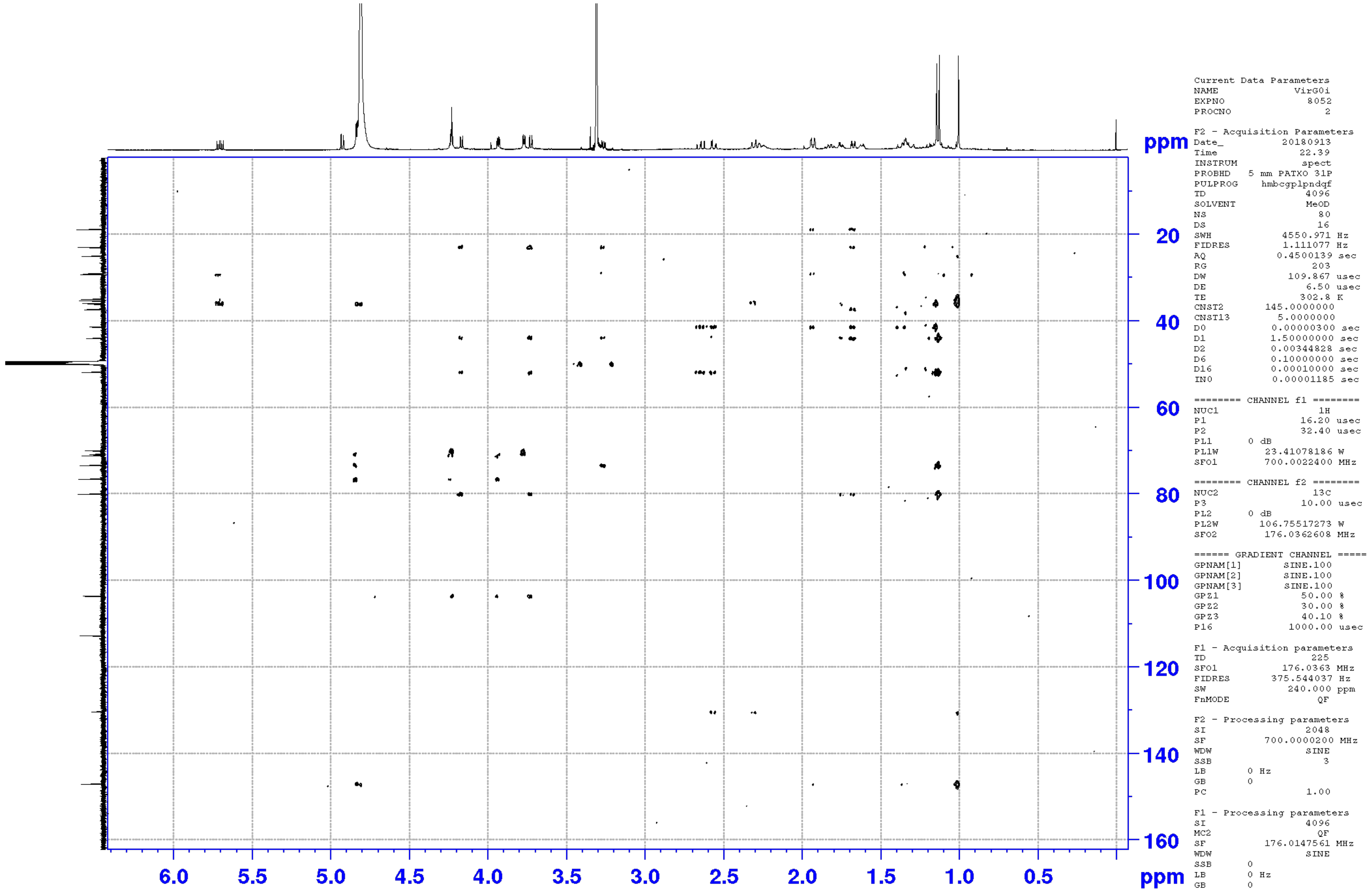

Figure S12. HMBC spectrum (700 MHz, CD_3OD) of **2**

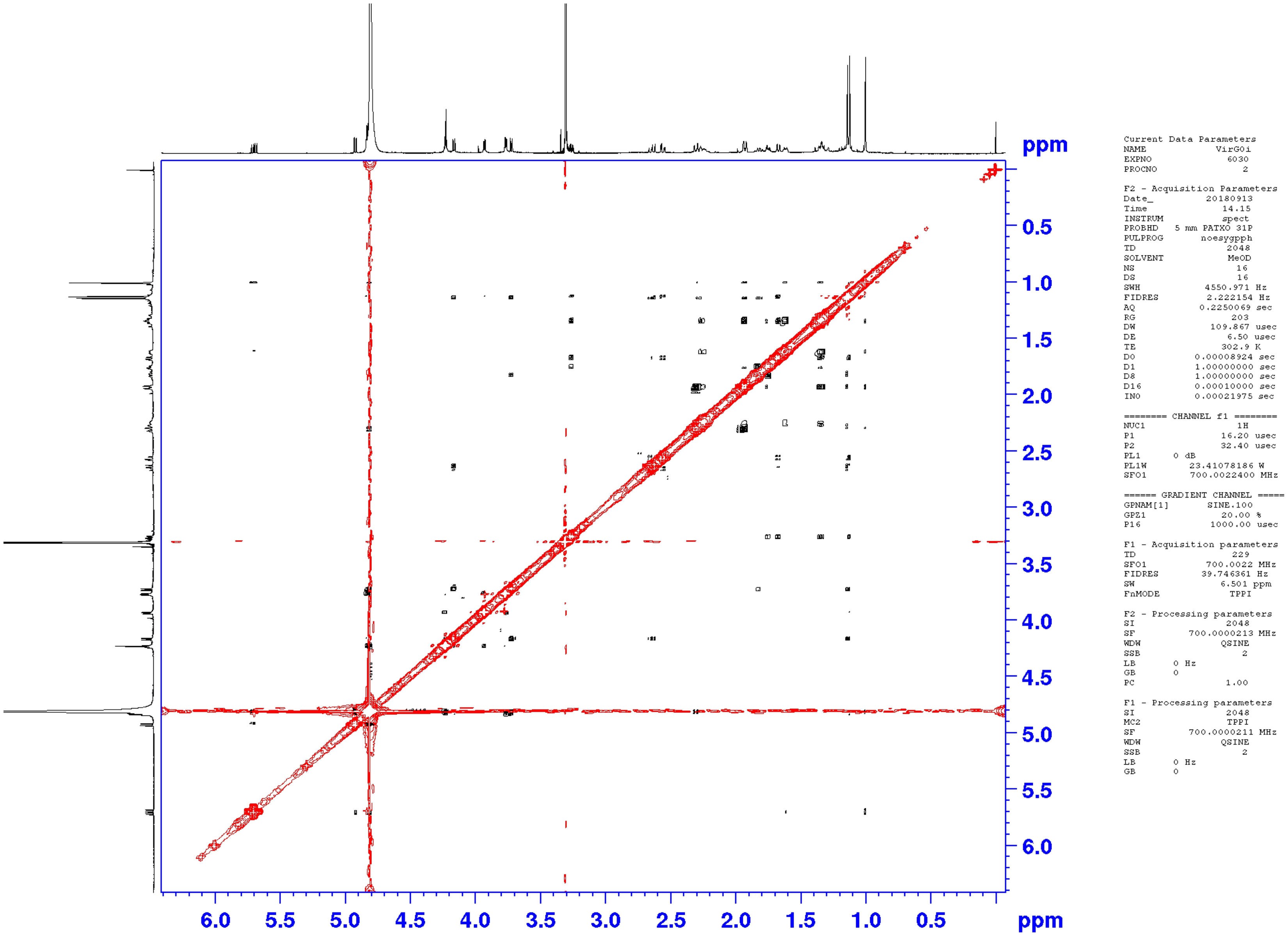

Figure S13. NOESY spectrum (700 MHz, CD_3OD) of **2**

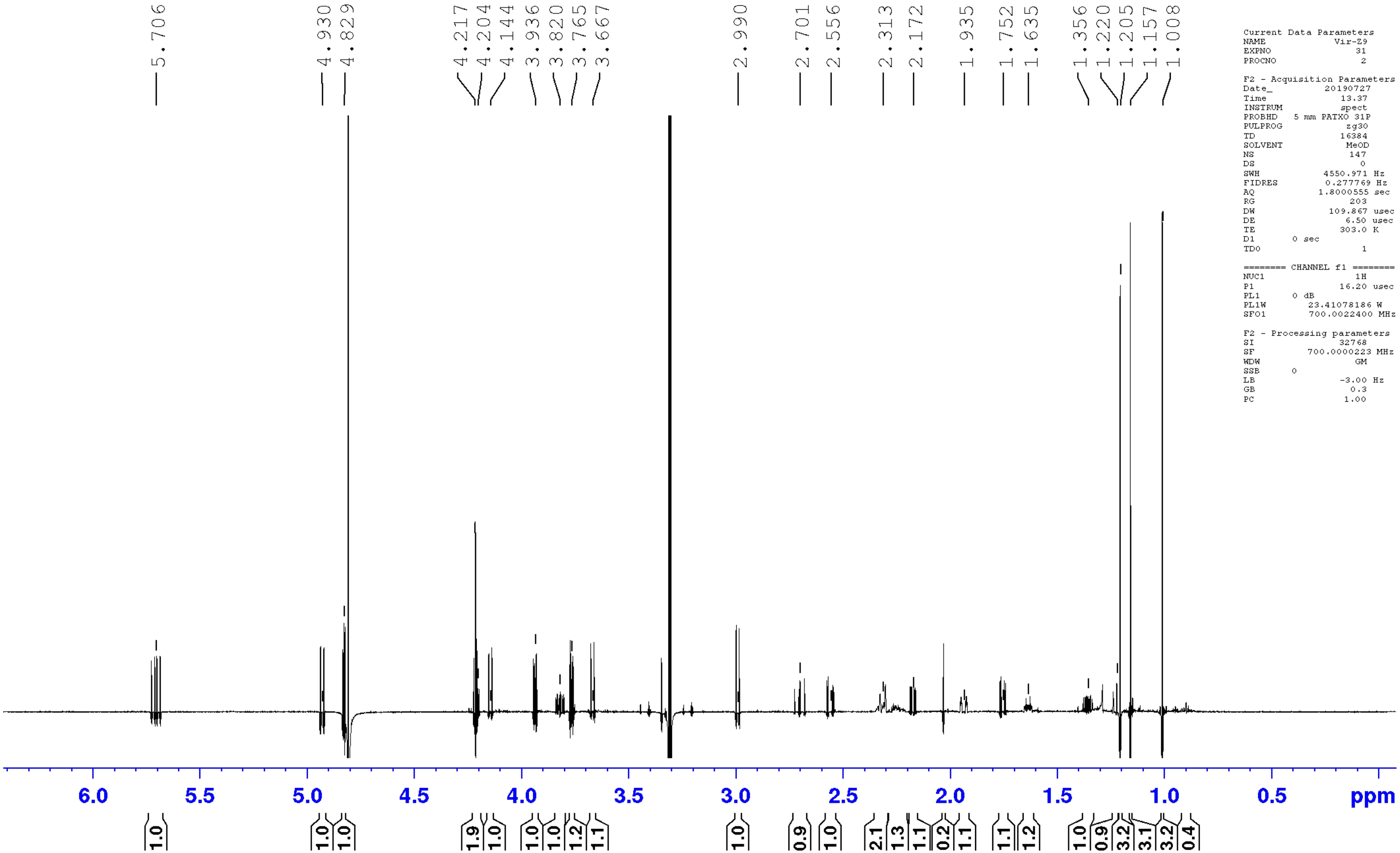

Figure S14. ^{1}H NMR spectrum (700 MHz, CD_3OD) of **3**

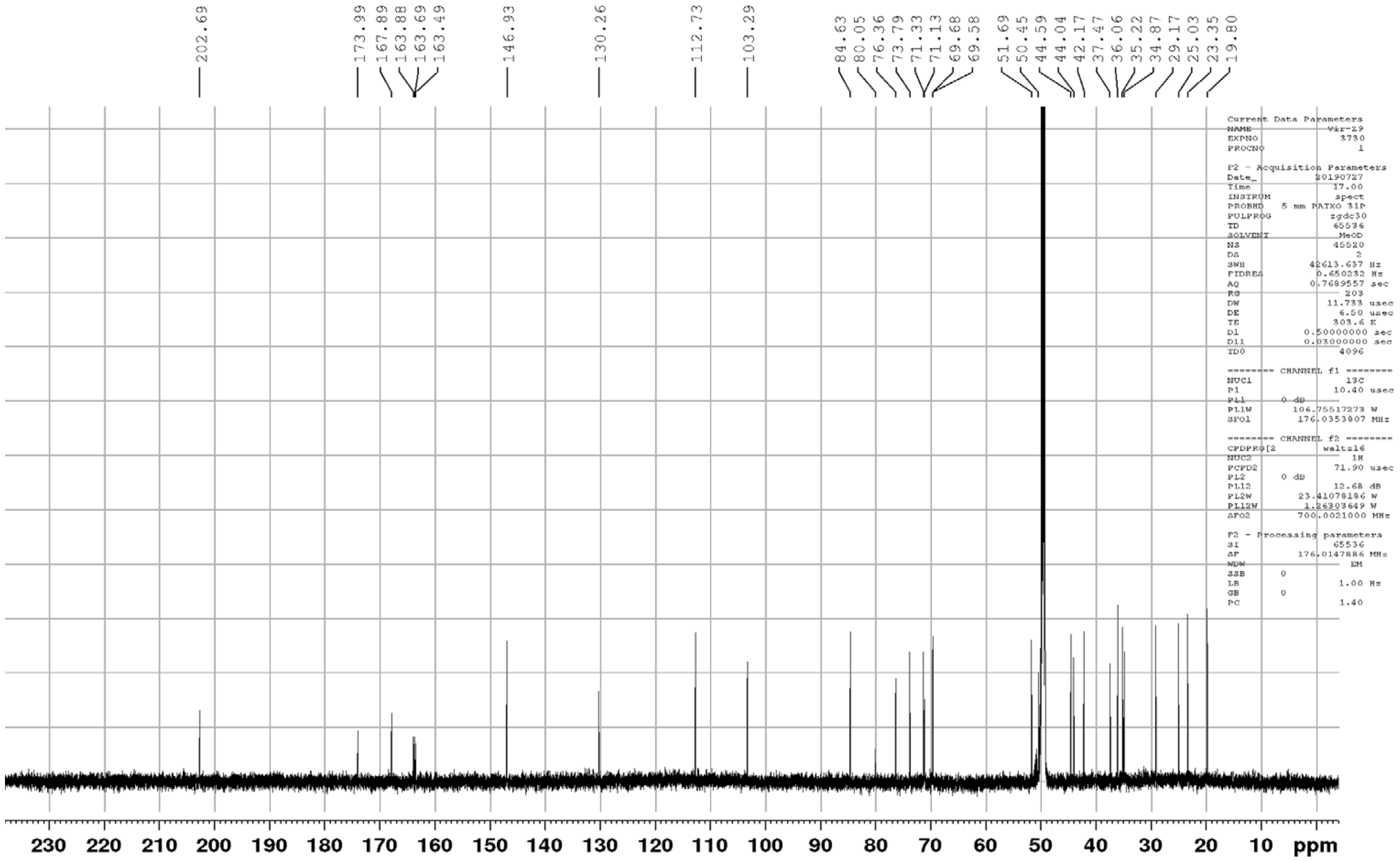

Figure S15. ^{13}C NMR spectrum (176 MHz, CD_3OD) of **3**

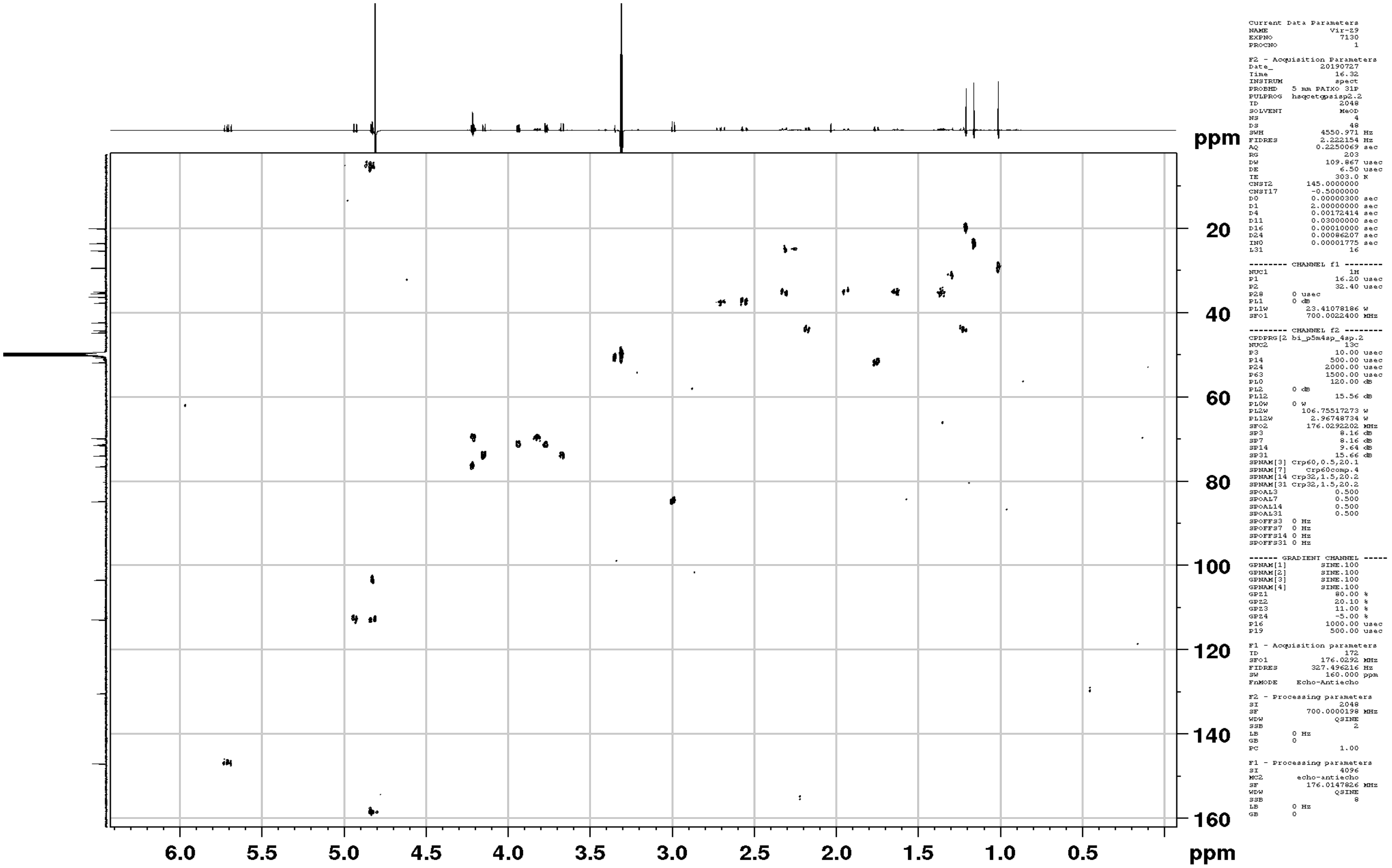

Figure S16. HSQC spectrum (700 MHz, CD_3OD) of **3**

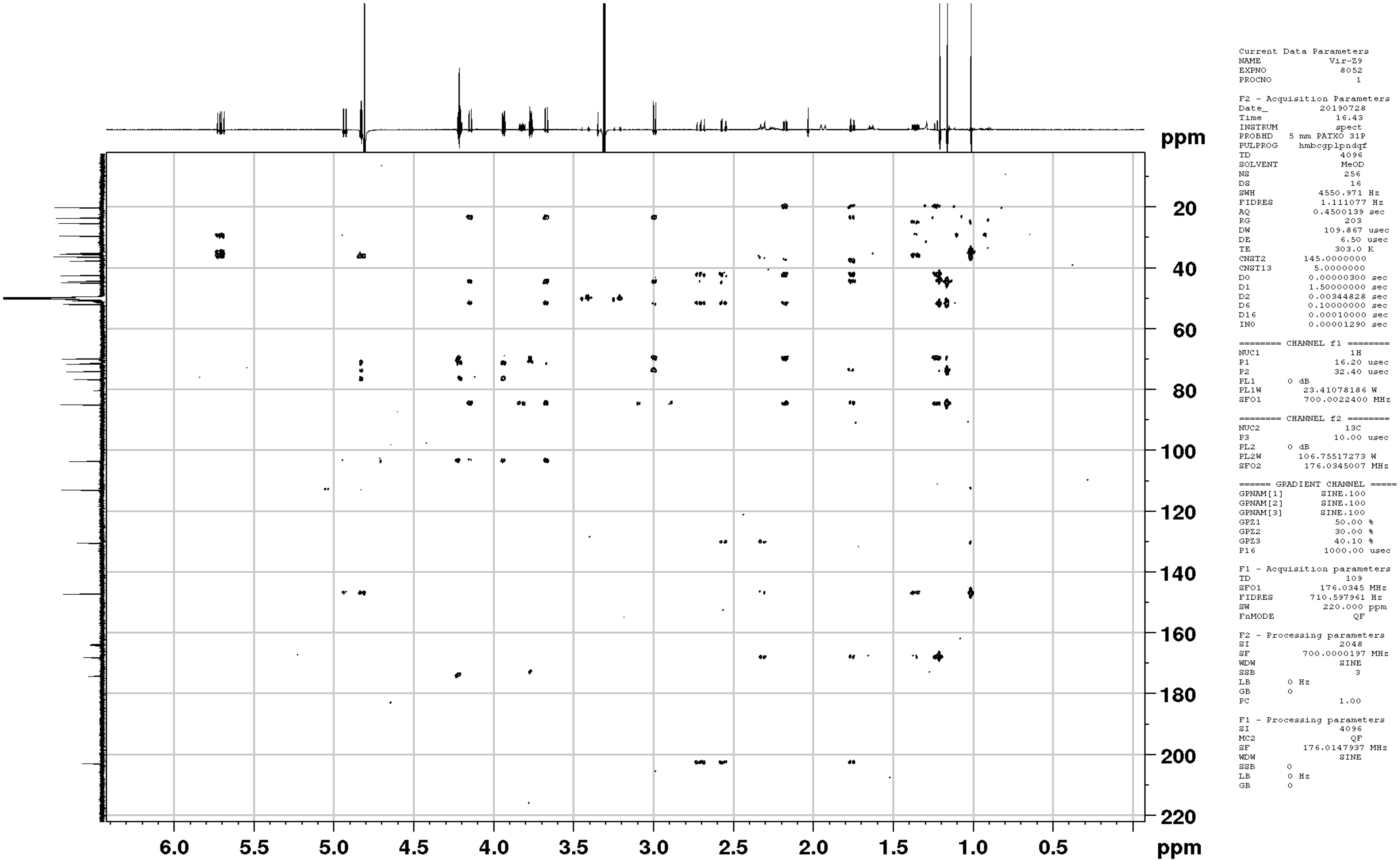

Figure S17. HMBC spectrum (700 MHz, CD_3OD) of **3**

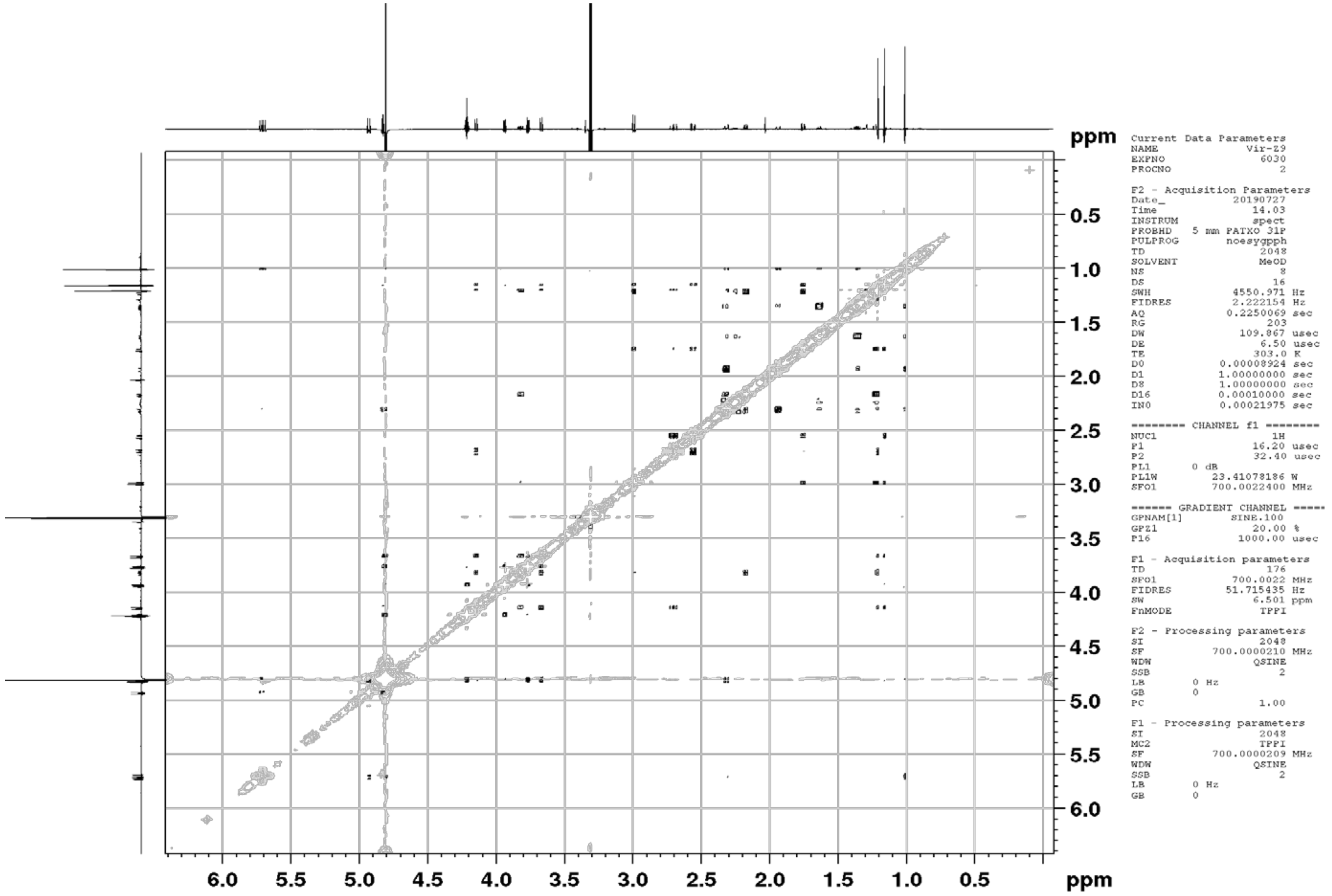

Figure S18. NOESY spectrum (700 MHz, CD_3OD) of **3**

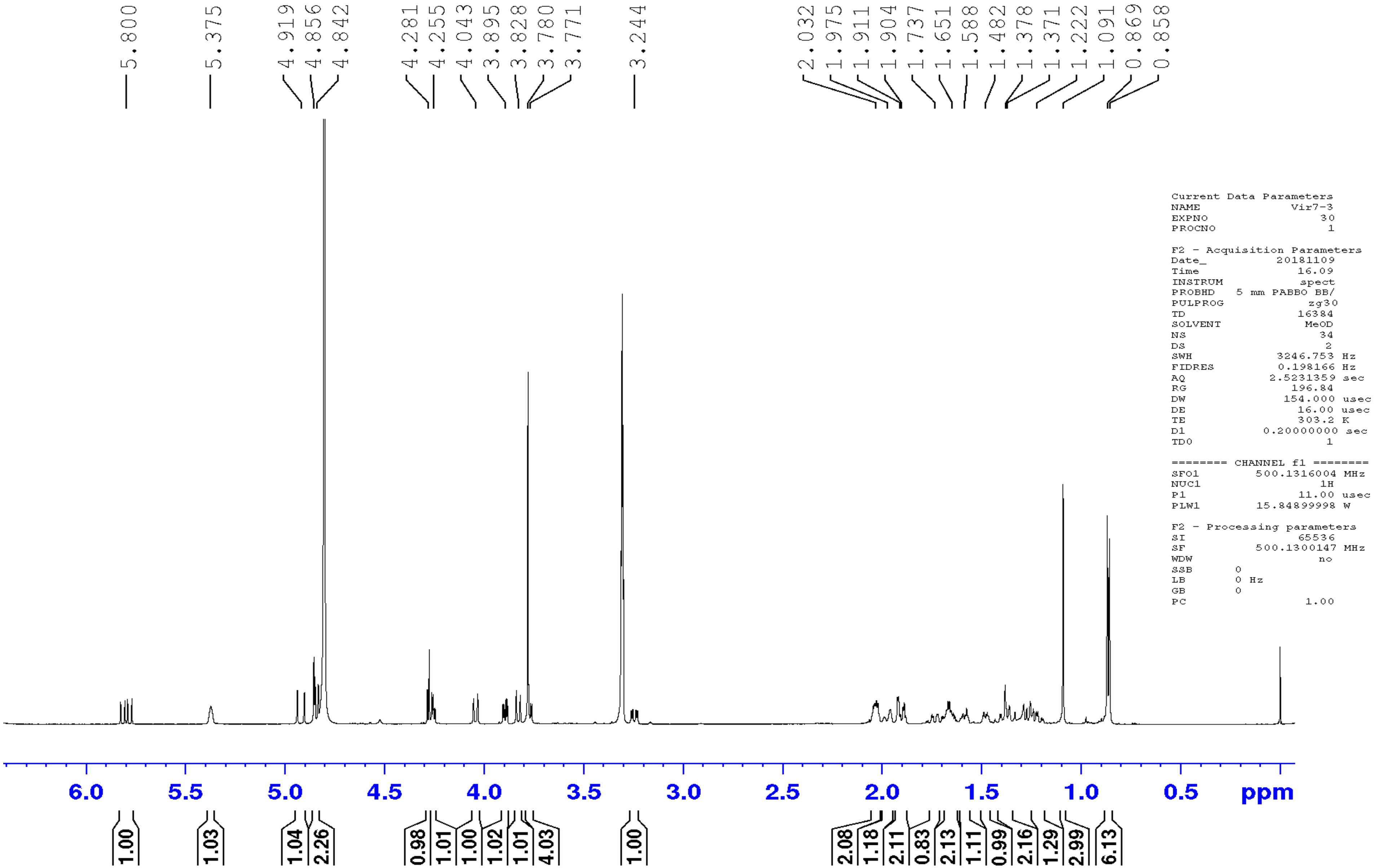

Figure S19. ^{1}H NMR spectrum (500 MHz, CD_3OD) of **4**

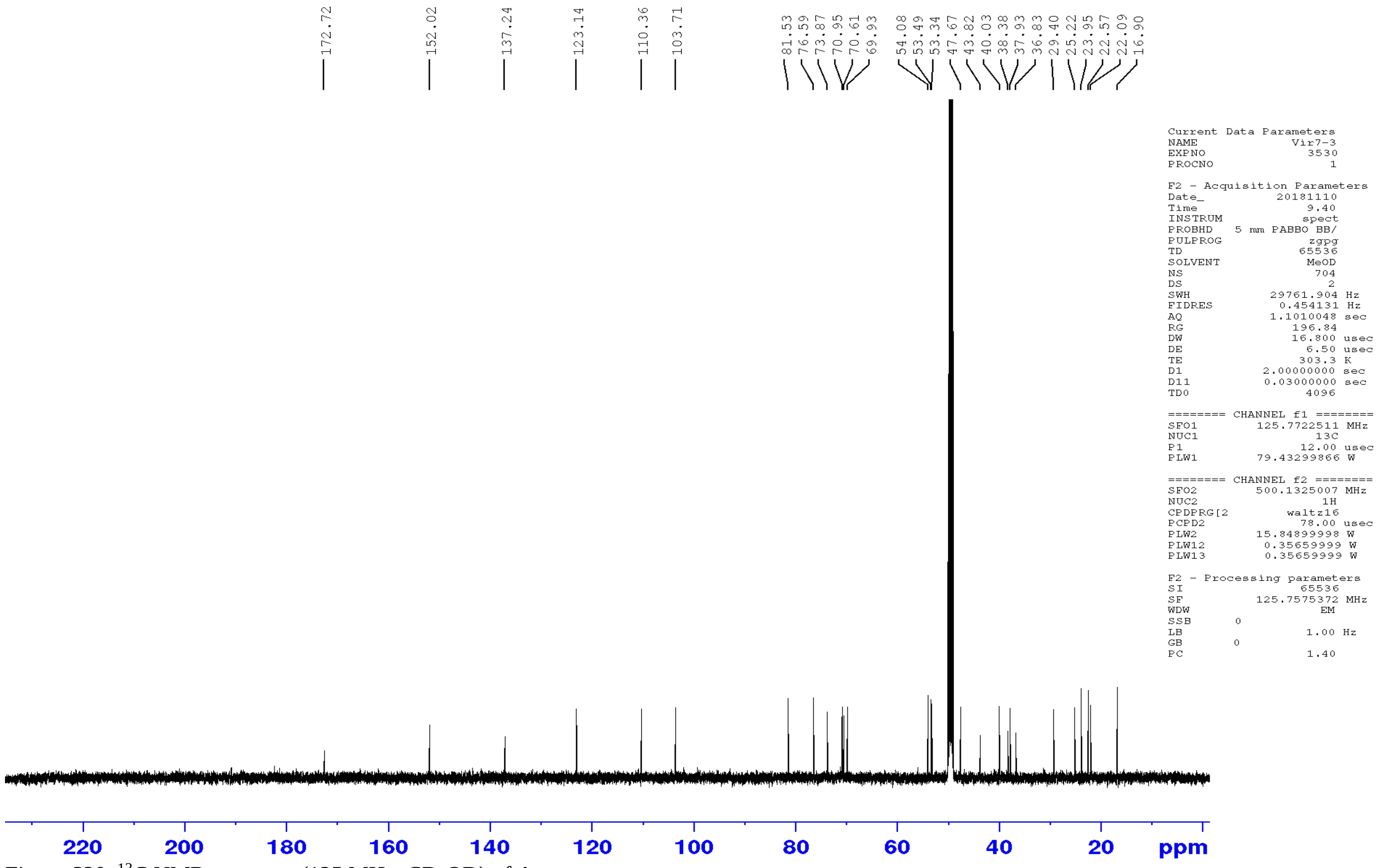

Figure S20. ^{13}C NMR spectrum (125 MHz, CD_3OD) of **4**

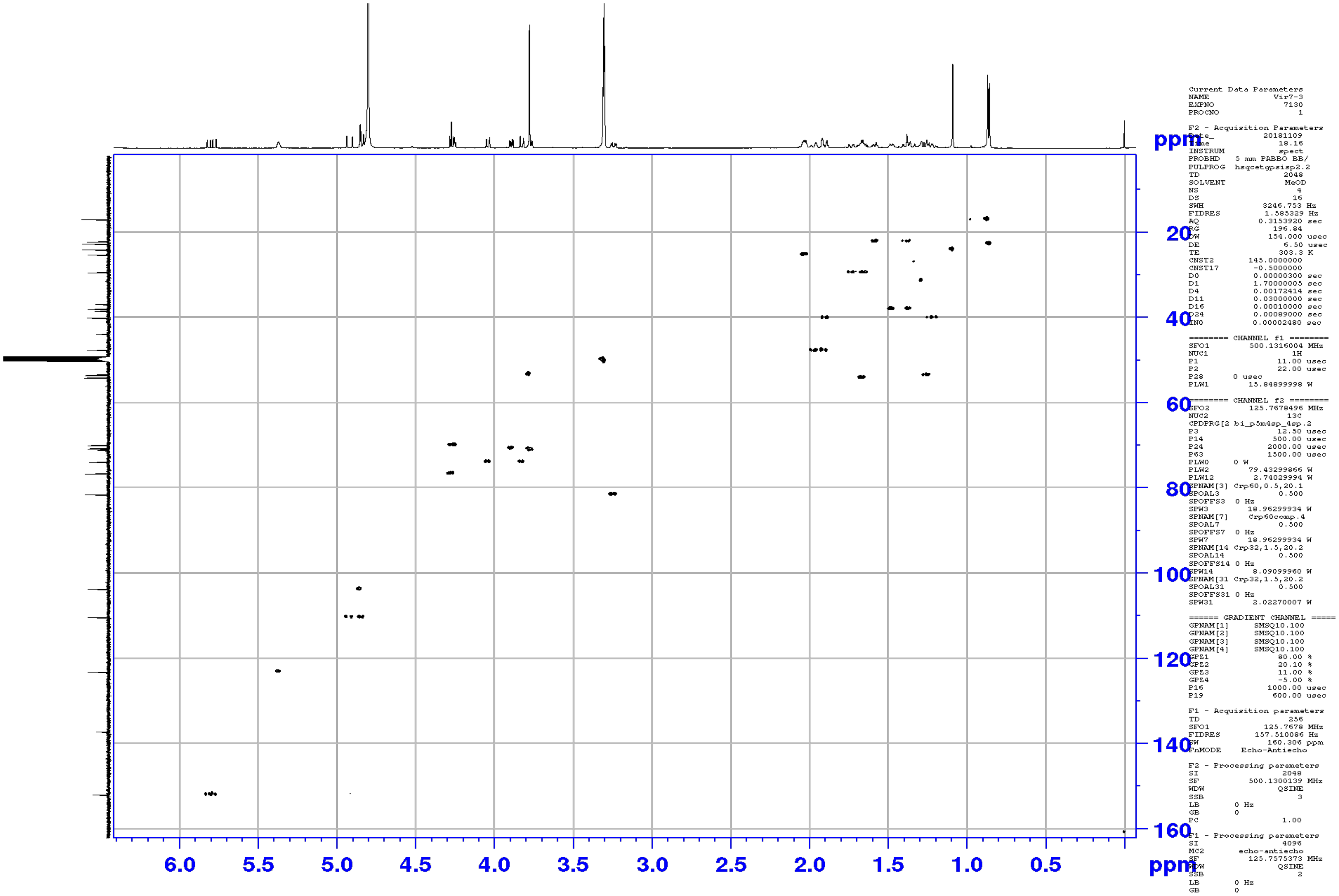

Figure S21. HSQC spectrum (500 MHz, CD_3OD) of **4**

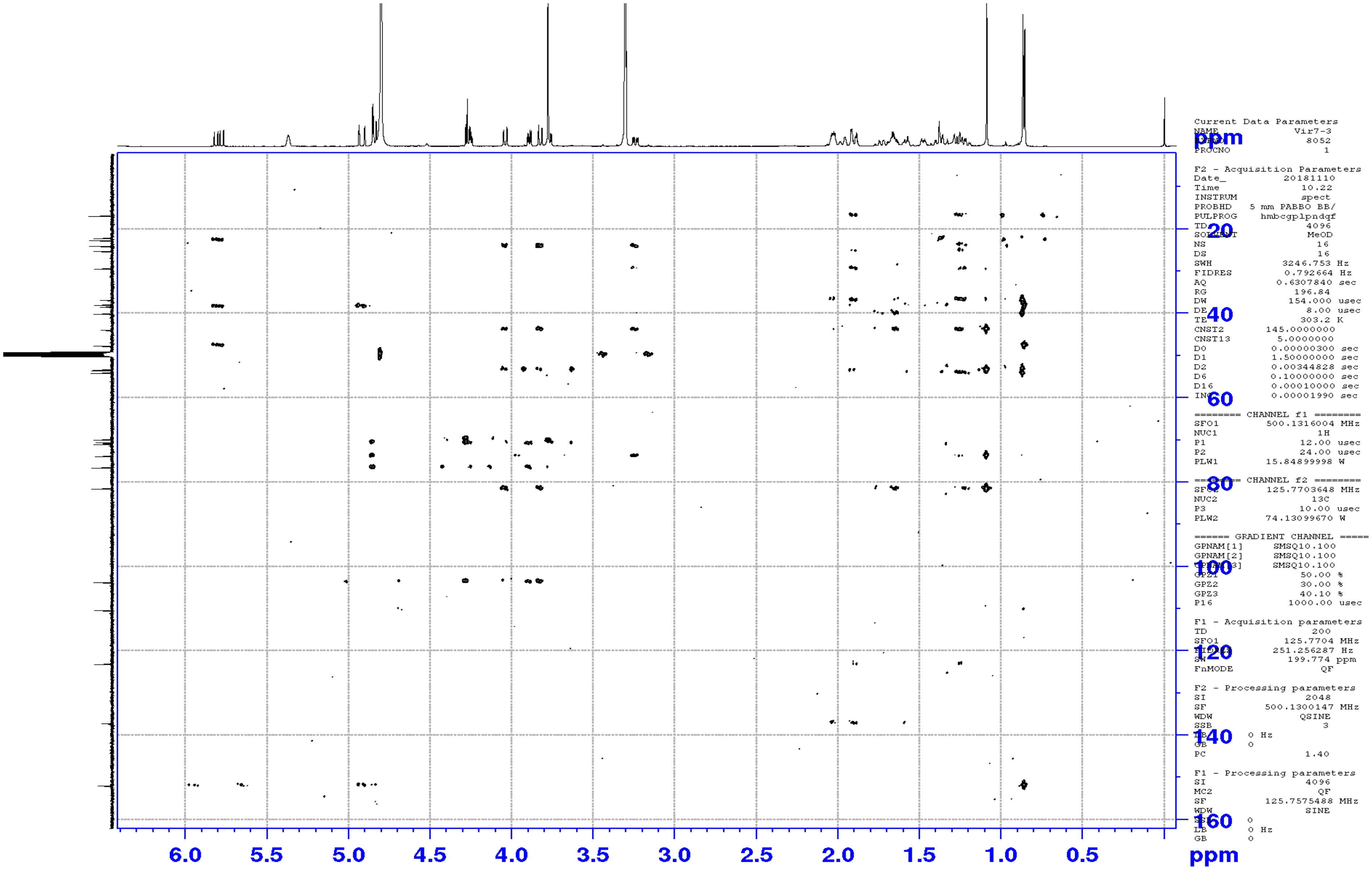

Figure S22. HMBC spectrum (500 MHz, CD_3OD) of **4**

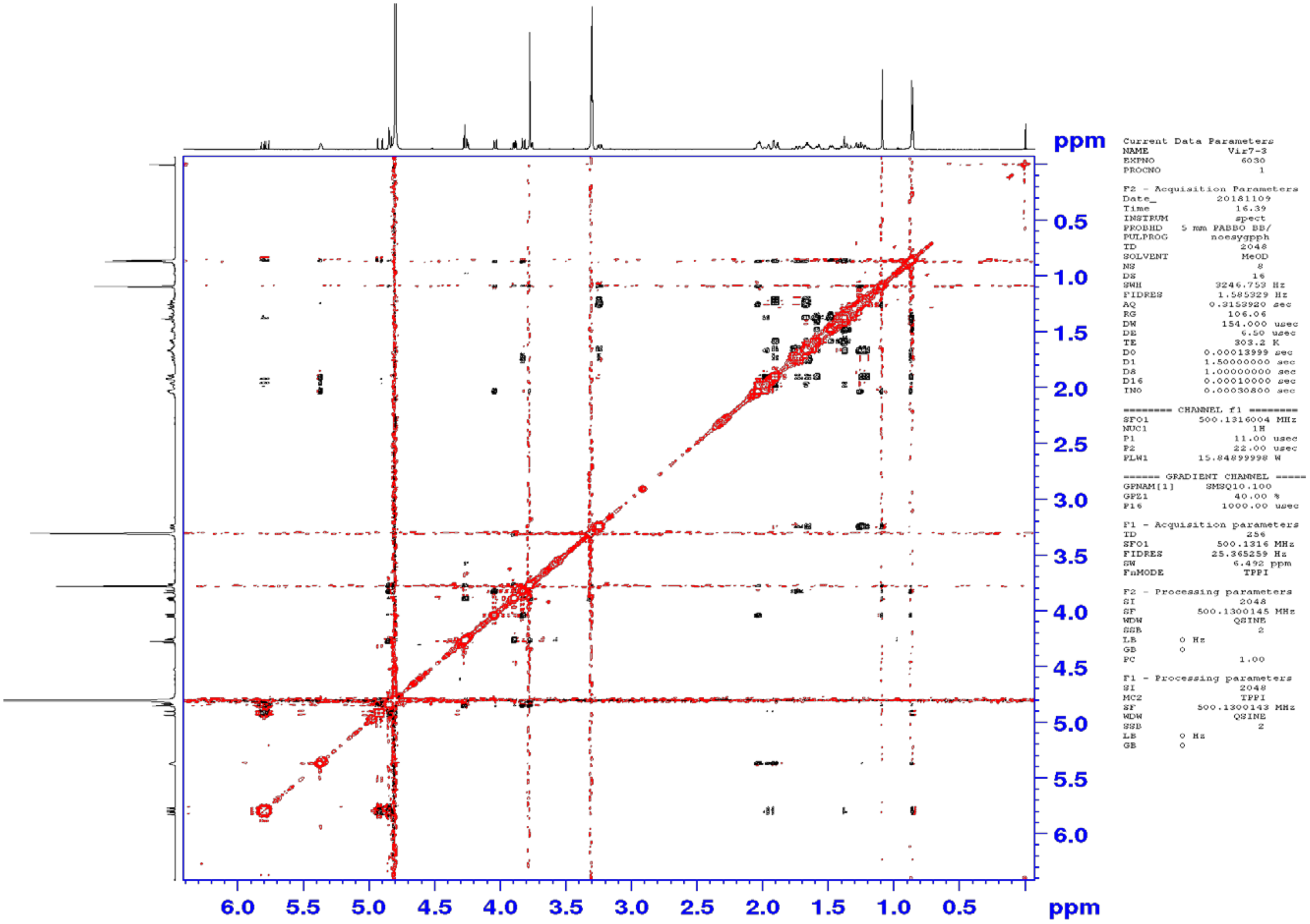

Figure S23. NOESY spectrum (500 MHz, CD_3OD) of **4**

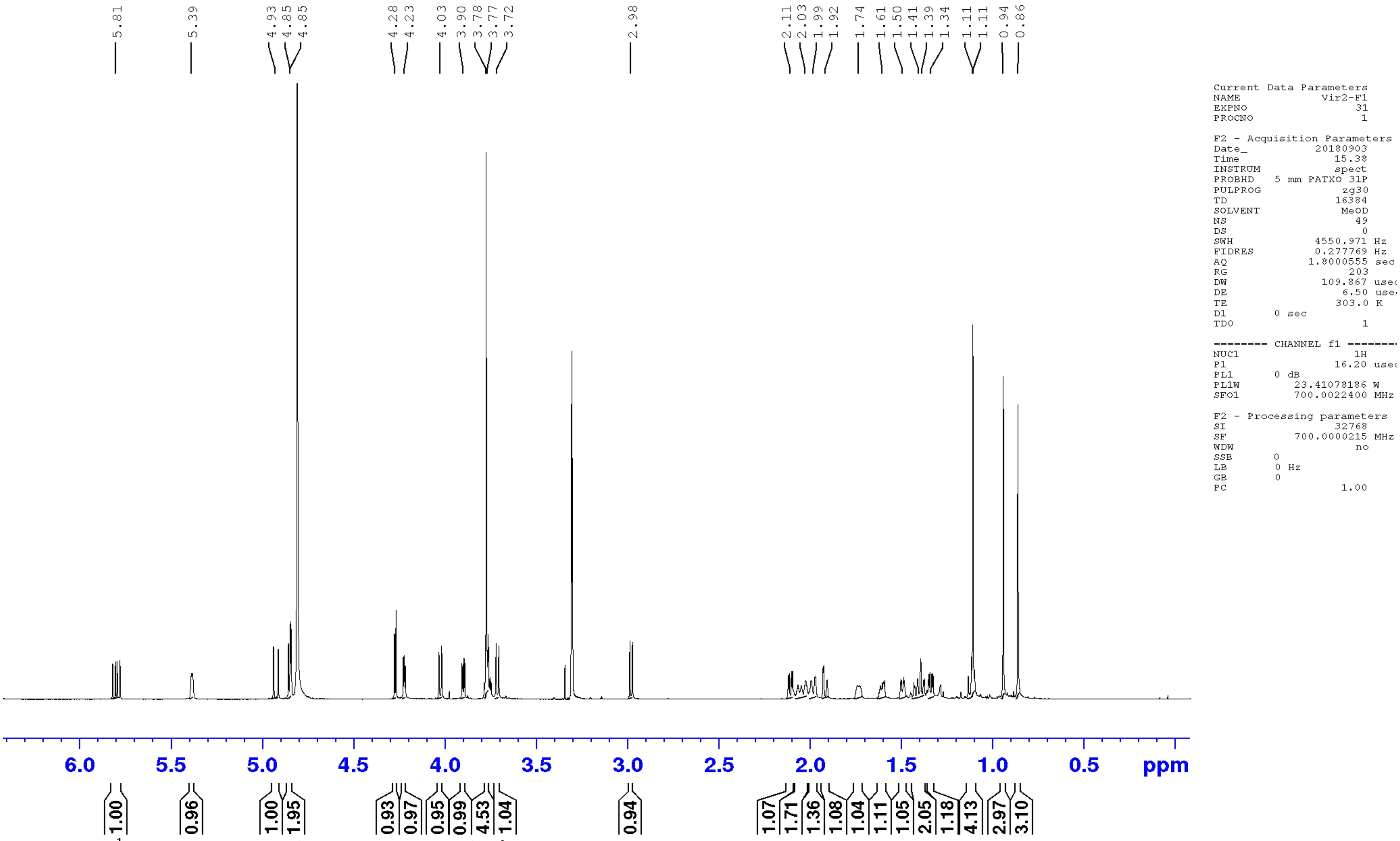

Figure S24. ^{1}H NMR spectrum (700 MHz, CD_3OD) of **5**

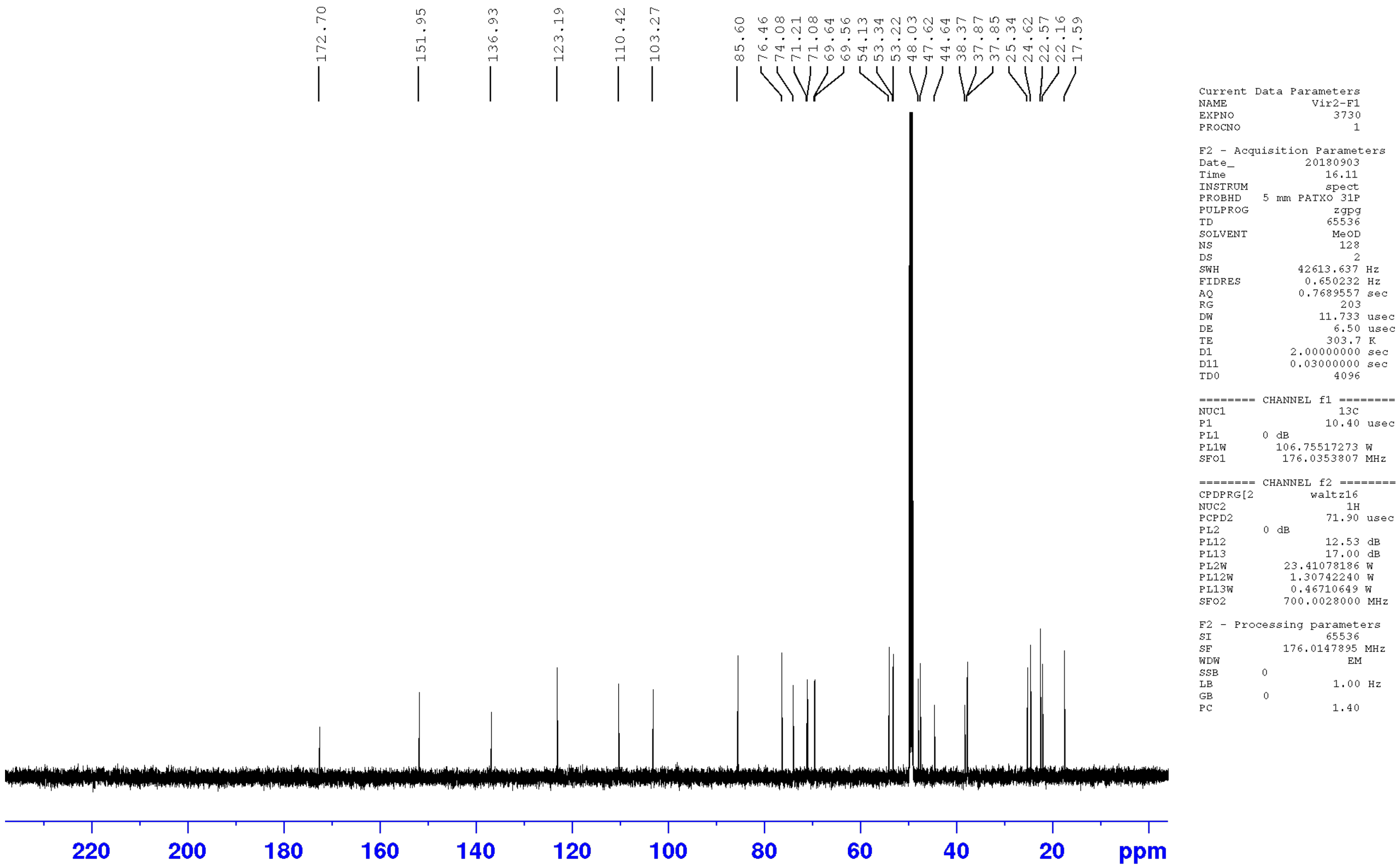

Figure S25. ^{13}C NMR spectrum (176 MHz, CD_3OD) of **5**

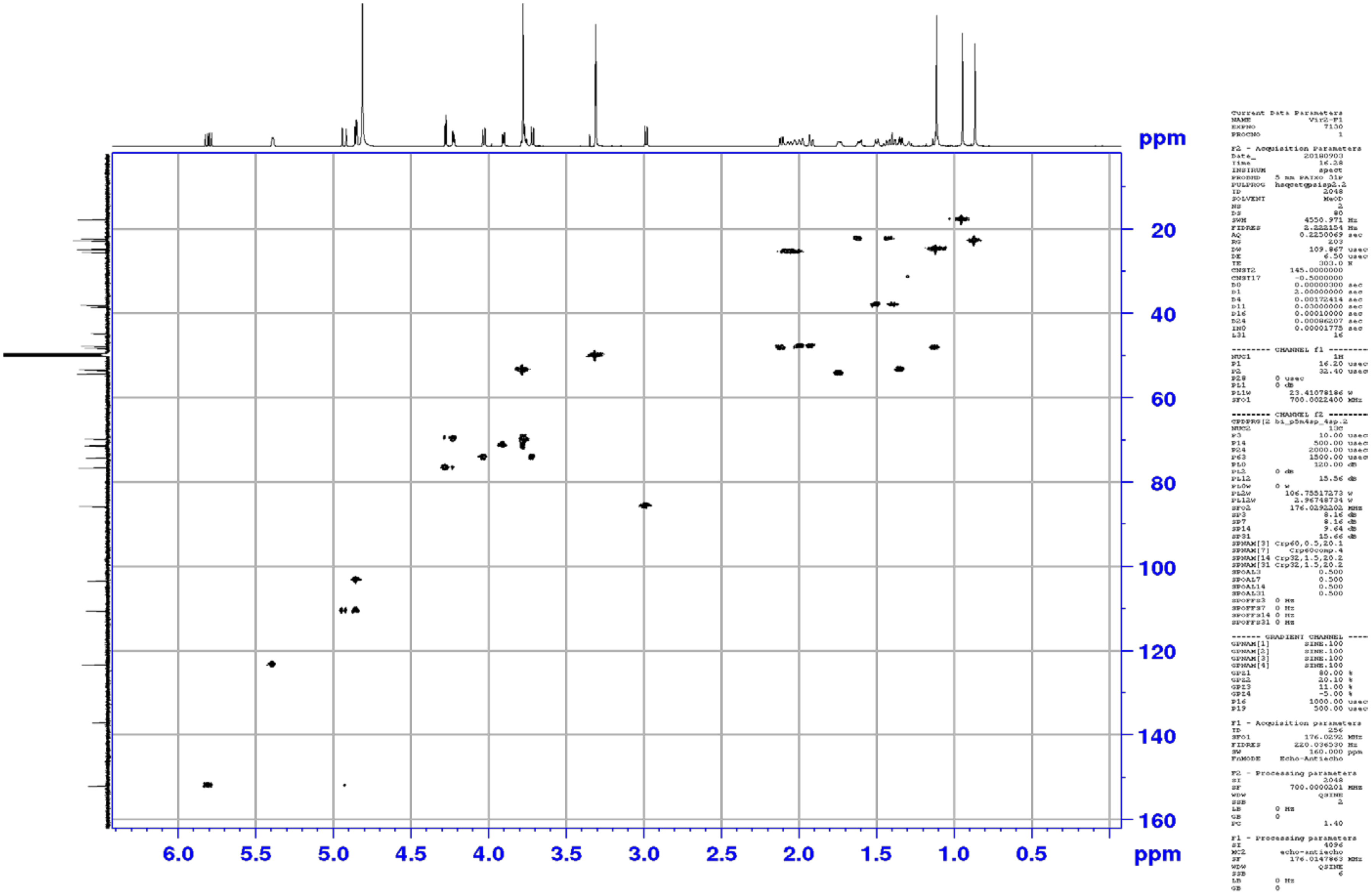

Figure S26. HSQC spectrum (700 MHz, CD_3OD) of **5**

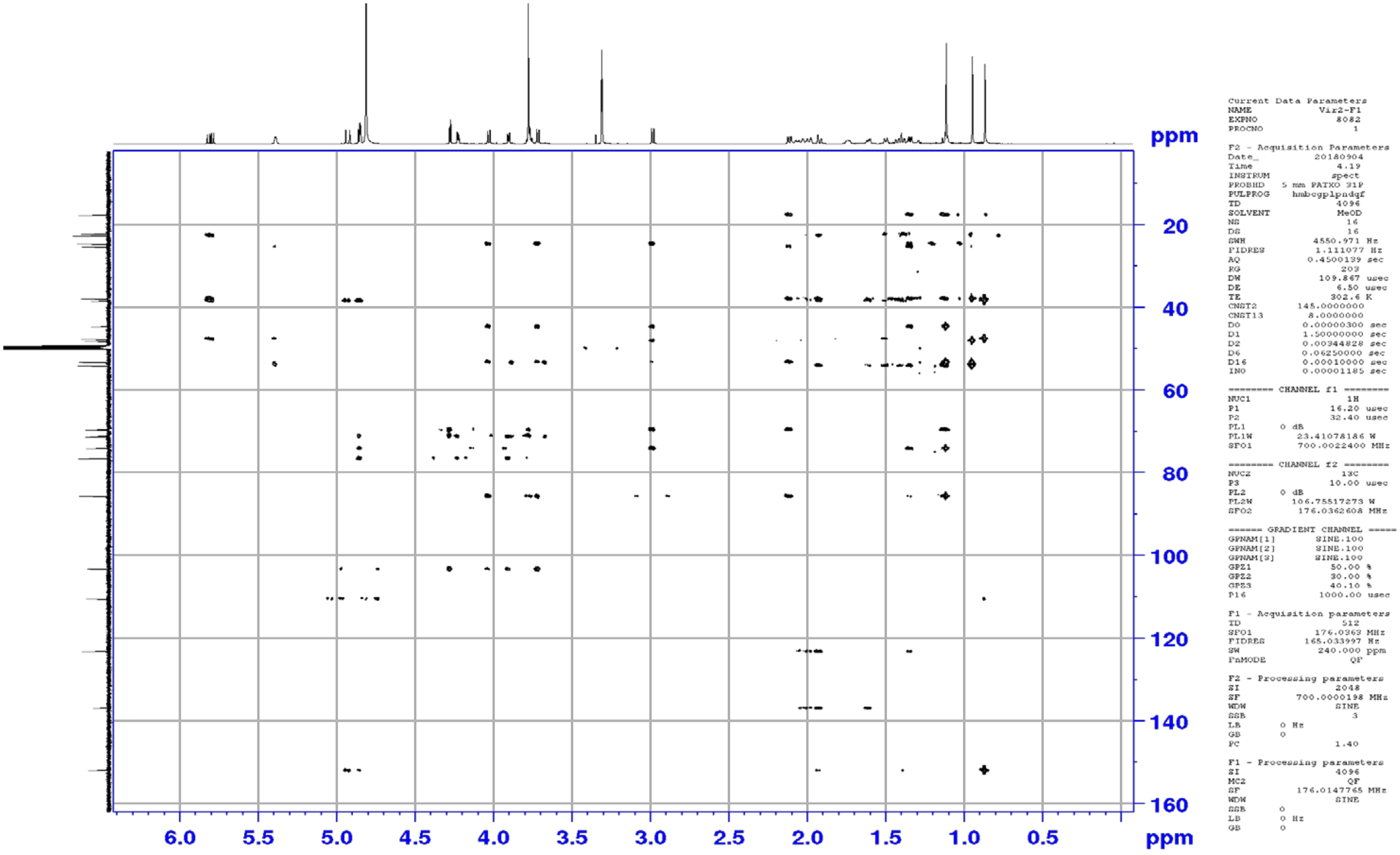

Figure S27. HMBC spectrum (700 MHz, CD_3OD) of **5**

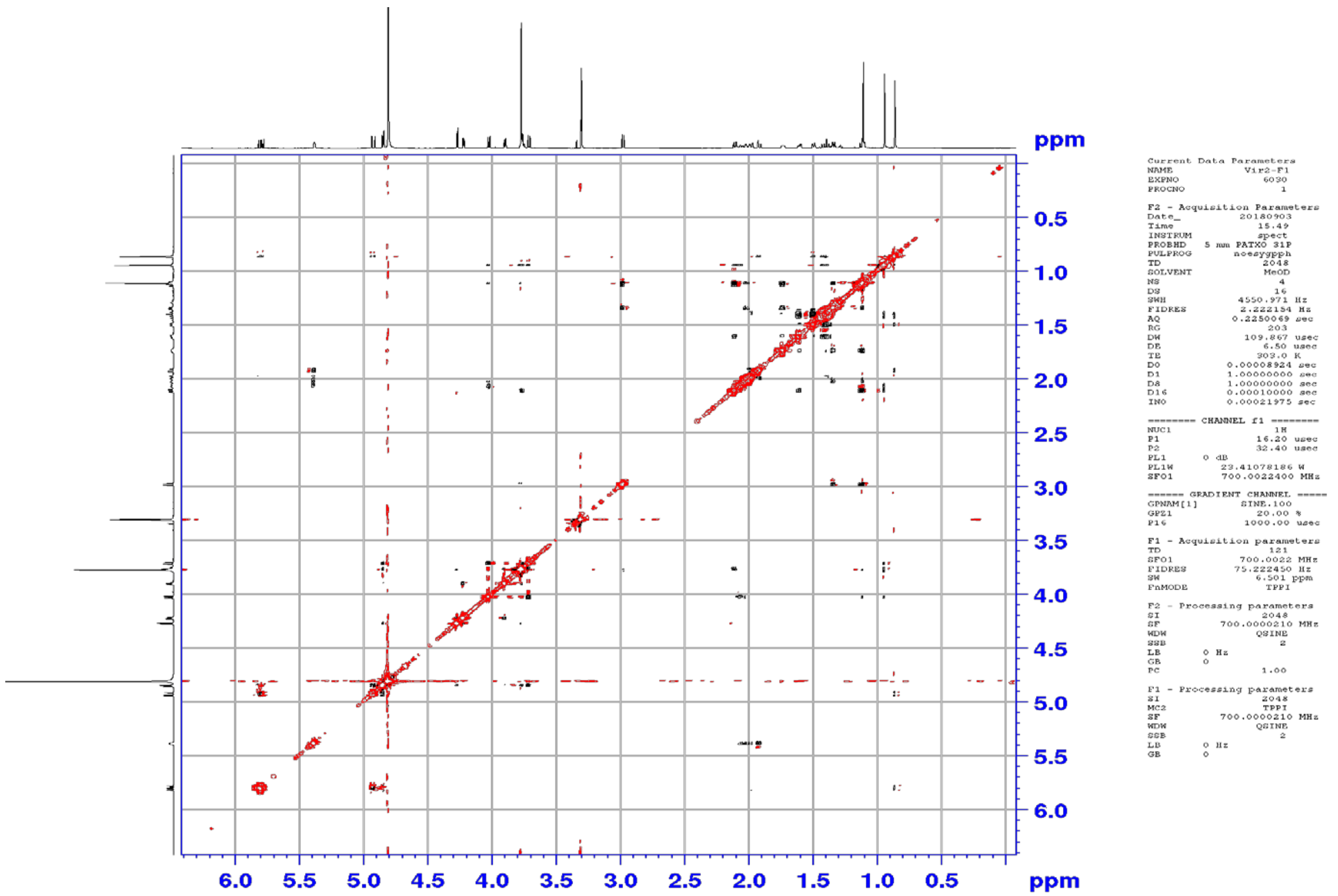

Figure S28. NOESY spectrum (700 MHz, CD_3OD) of **5**

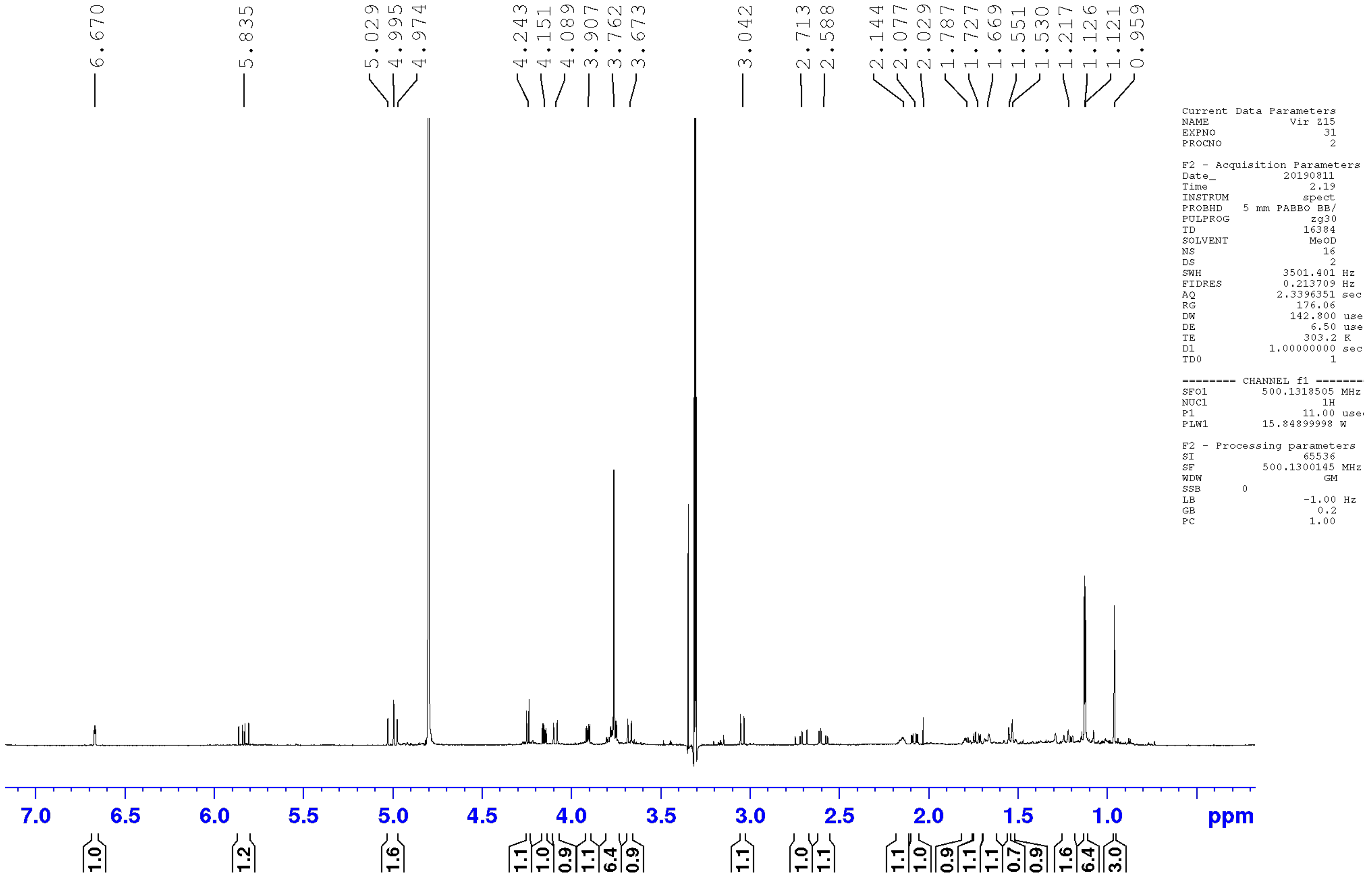

Figure S29. ^{1}H NMR spectrum (500 MHz, CD_3OD) of **6**

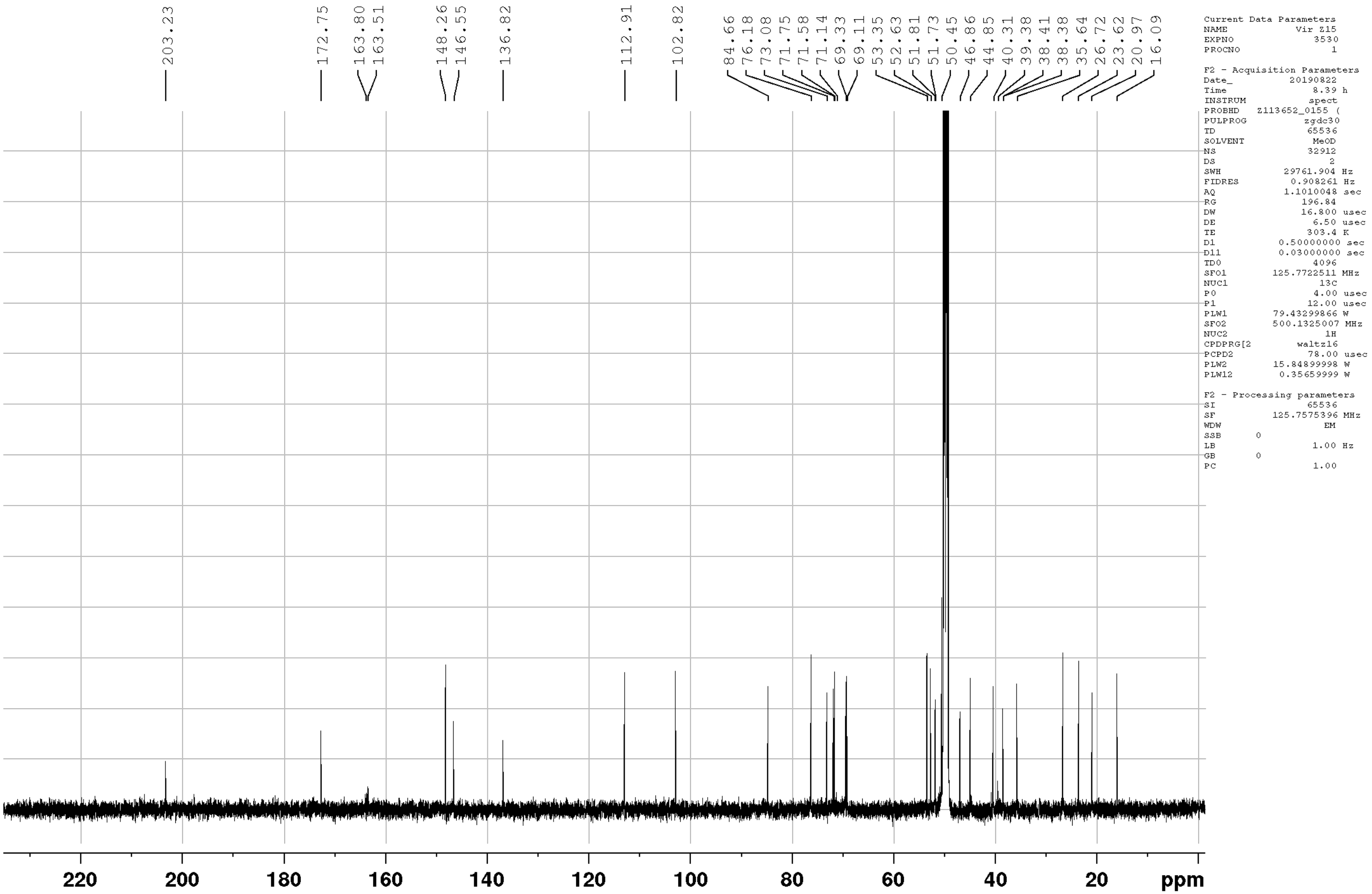

Figure S30. ^{13}C NMR spectrum (125 MHz, CD_3OD) of **6**

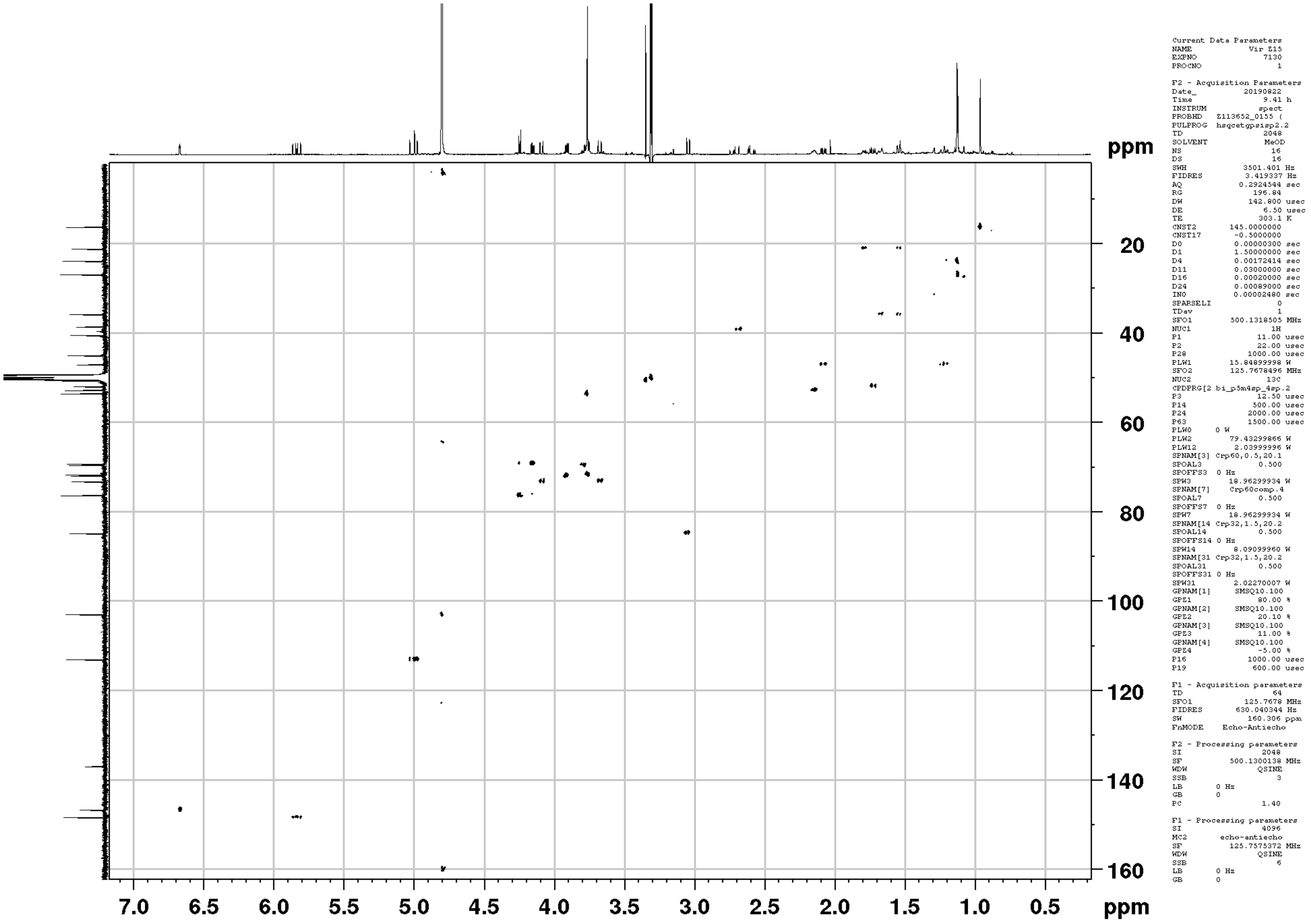

Figure S31. HSQC spectrum (500 MHz, CD_3OD) of **6**

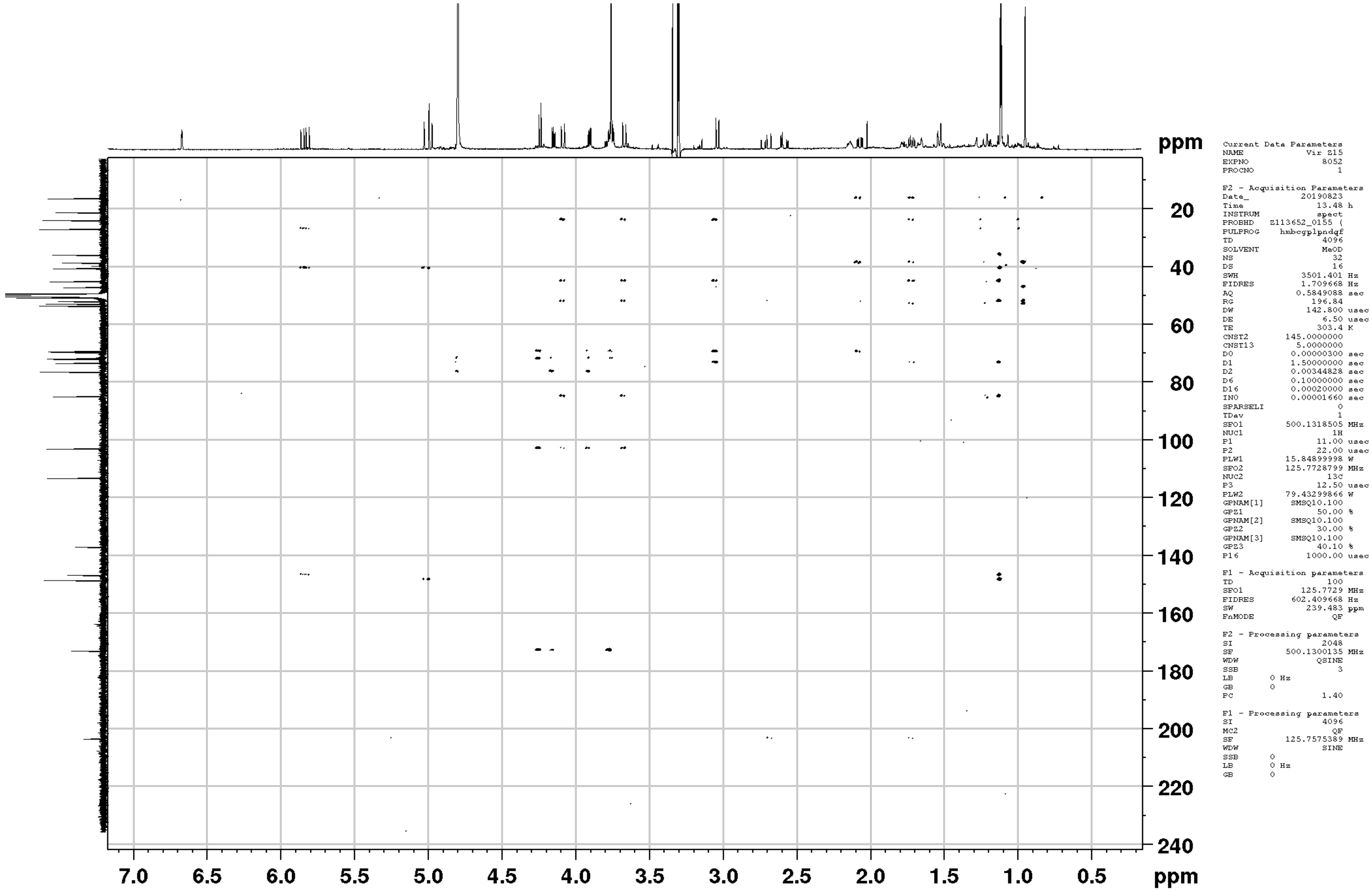

Figure S32. HMBC spectrum (500 MHz, CD_3OD) of **6**

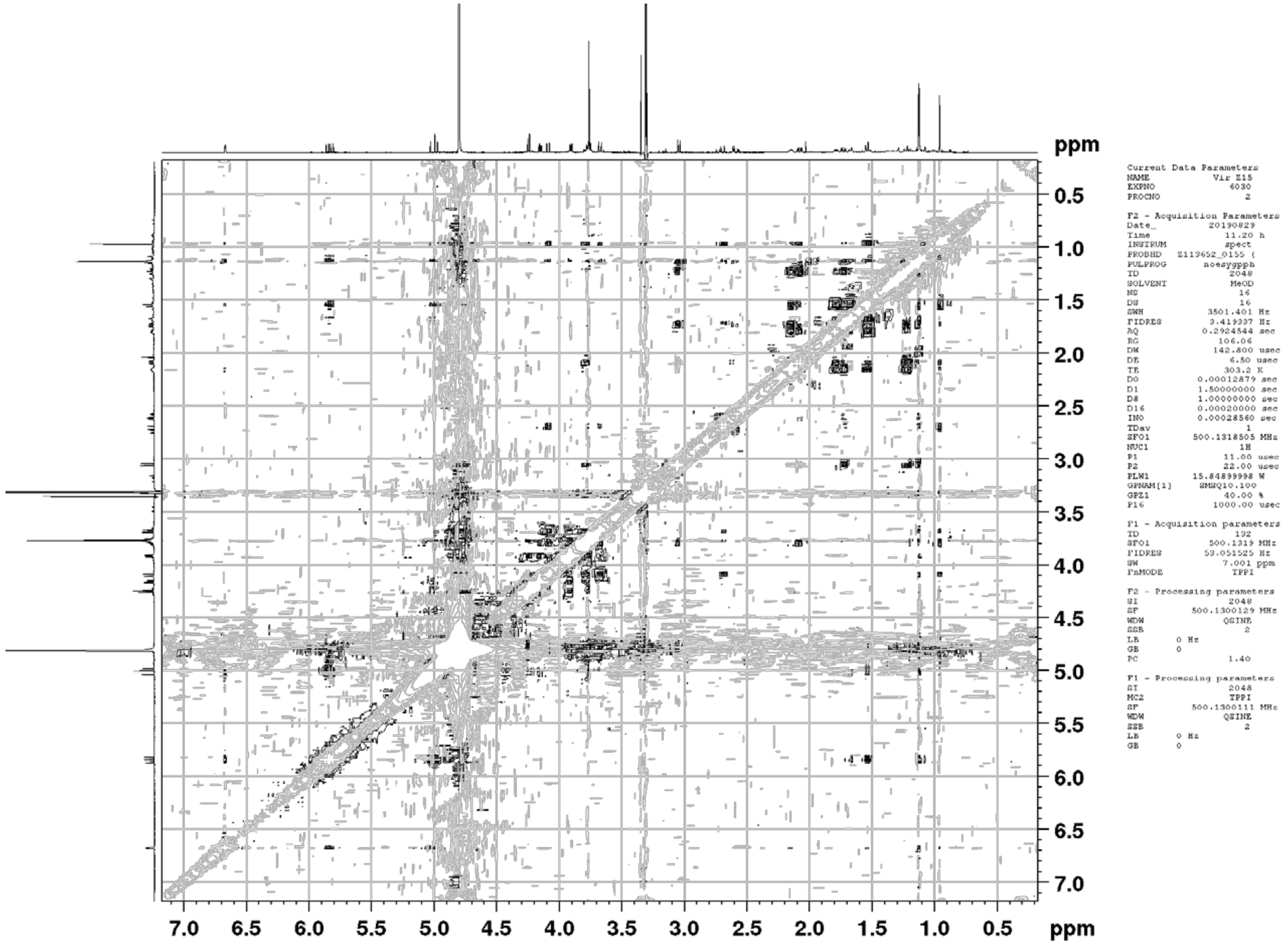

Figure S33. NOESY spectrum (500 MHz, CD_3OD) of **6**

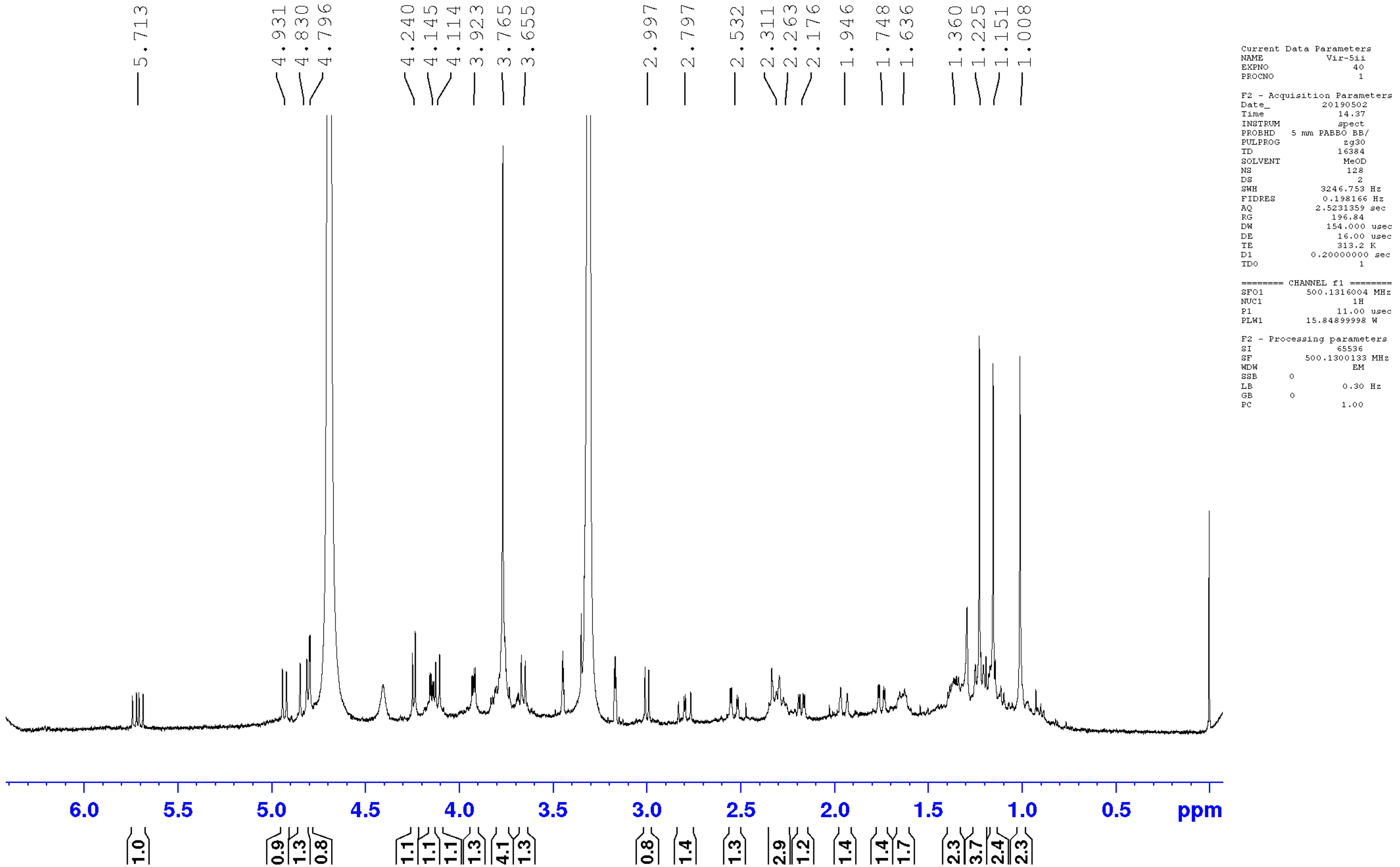

Figure S34. ^{1}H NMR spectrum (700 MHz, CD_3OD) of **7**

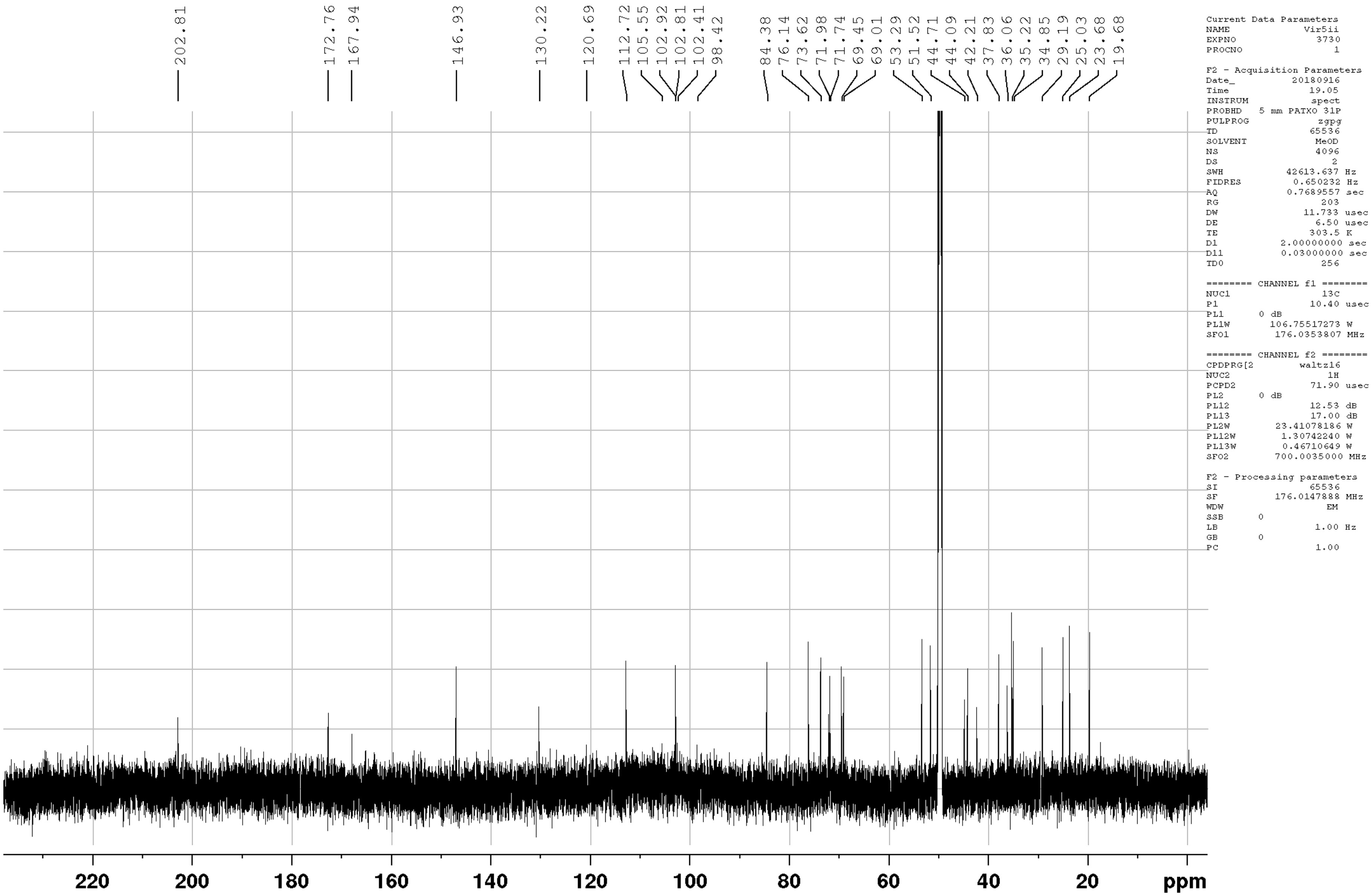

Figure S35. ^{13}C NMR spectrum (176 MHz, CD_3OD) of **7**

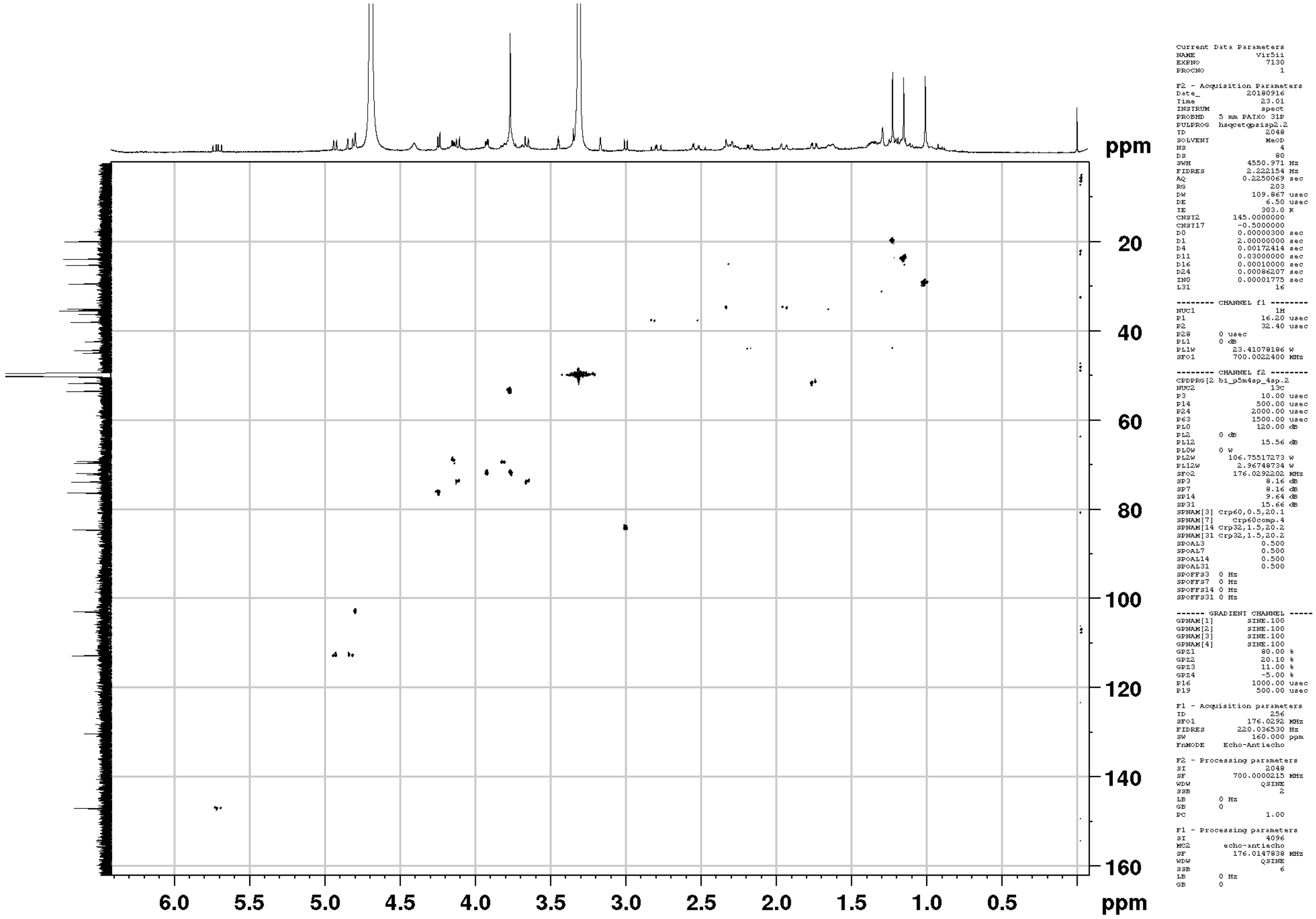

Figure S36. HSQC spectrum (700 MHz, CD_3OD) of **7**

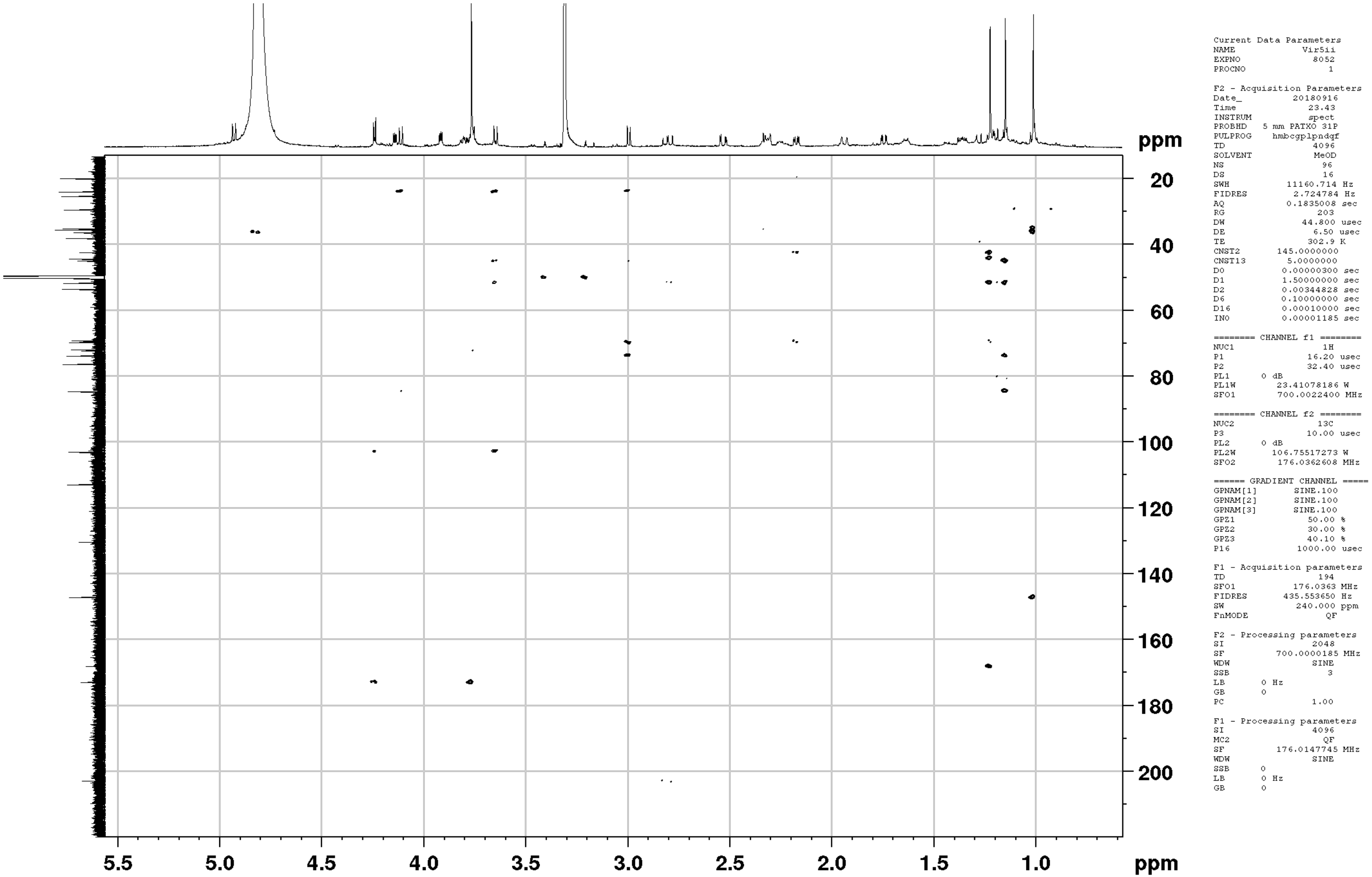

Figure S37. HMBC spectrum (700 MHz, CD_3OD) of **7**

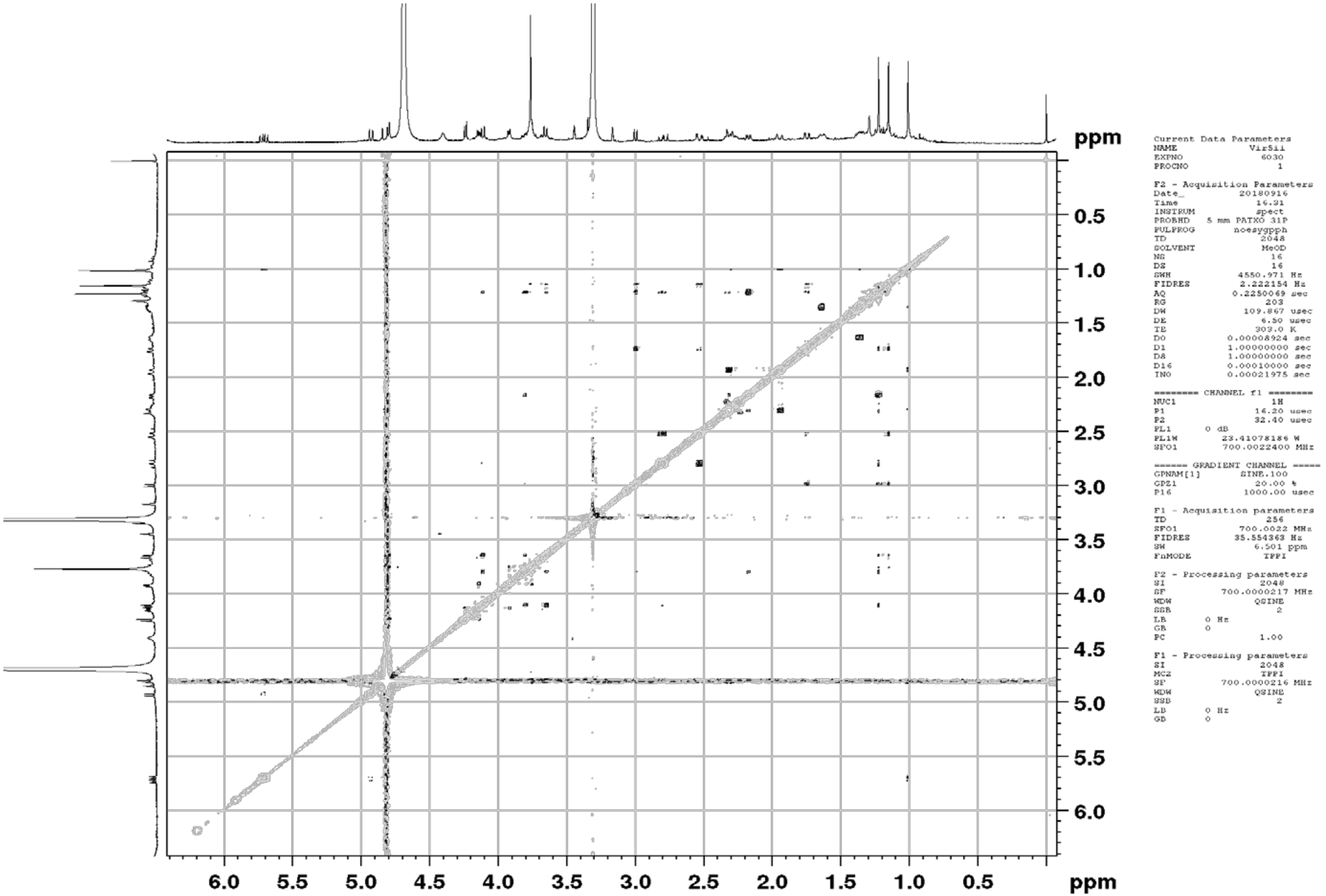

Figure S38. NOESY spectrum (700 MHz, CD_3OD) of **7**

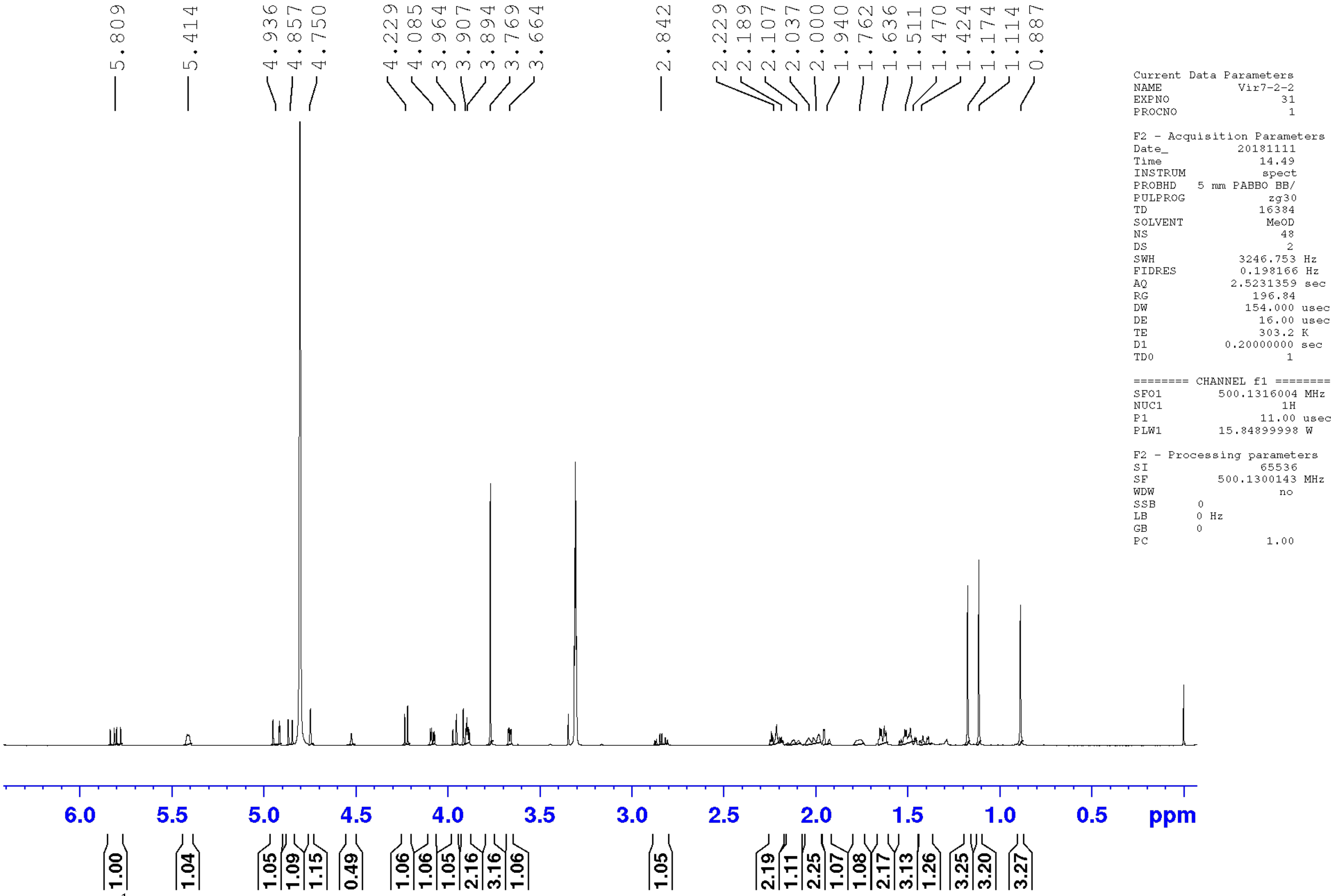

Figure S39. ^{1}H NMR spectrum (500 MHz, CD_3OD) of **8**

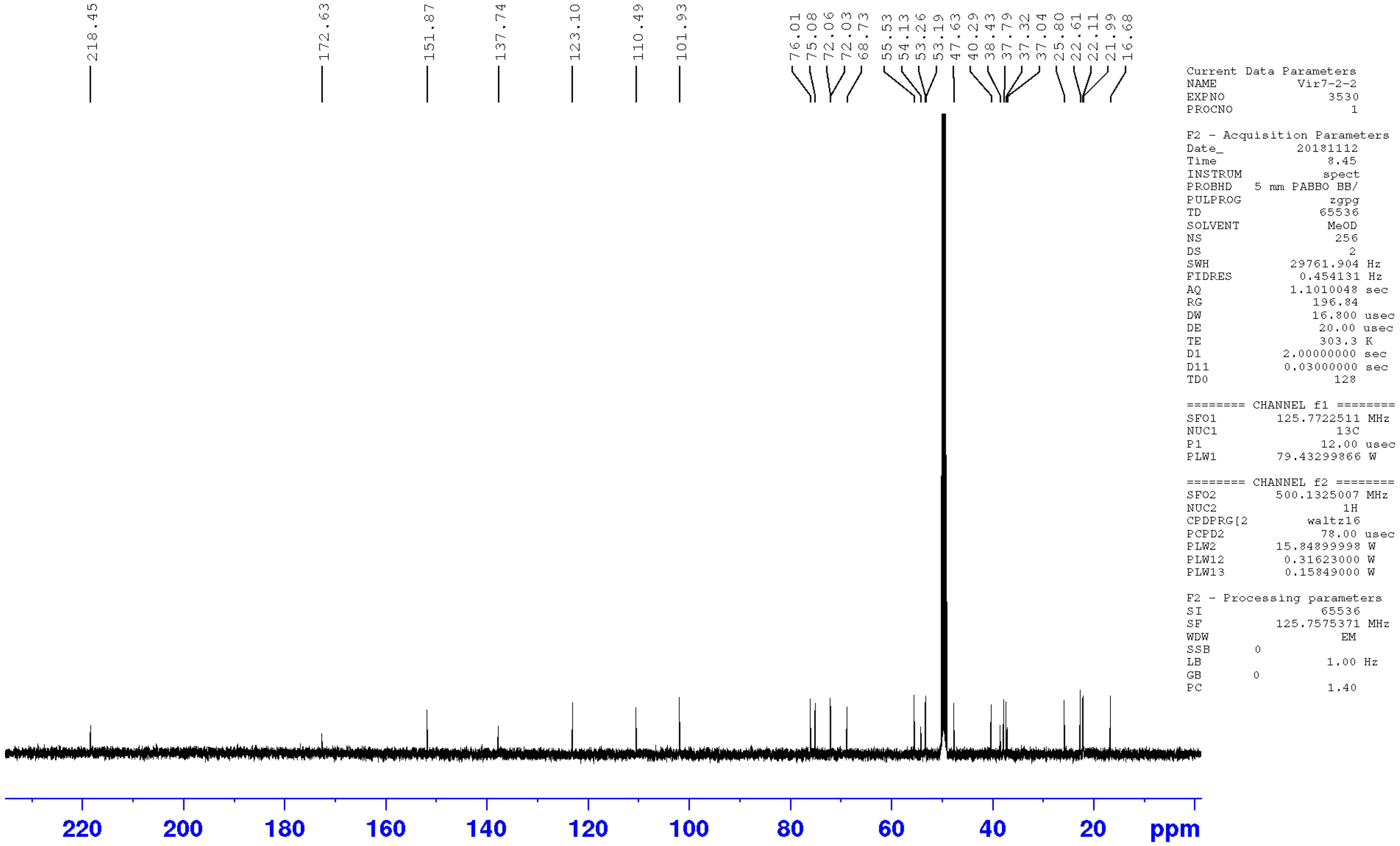

Figure S40. ^{13}C NMR spectrum (125 MHz, CD_3OD) of **8**

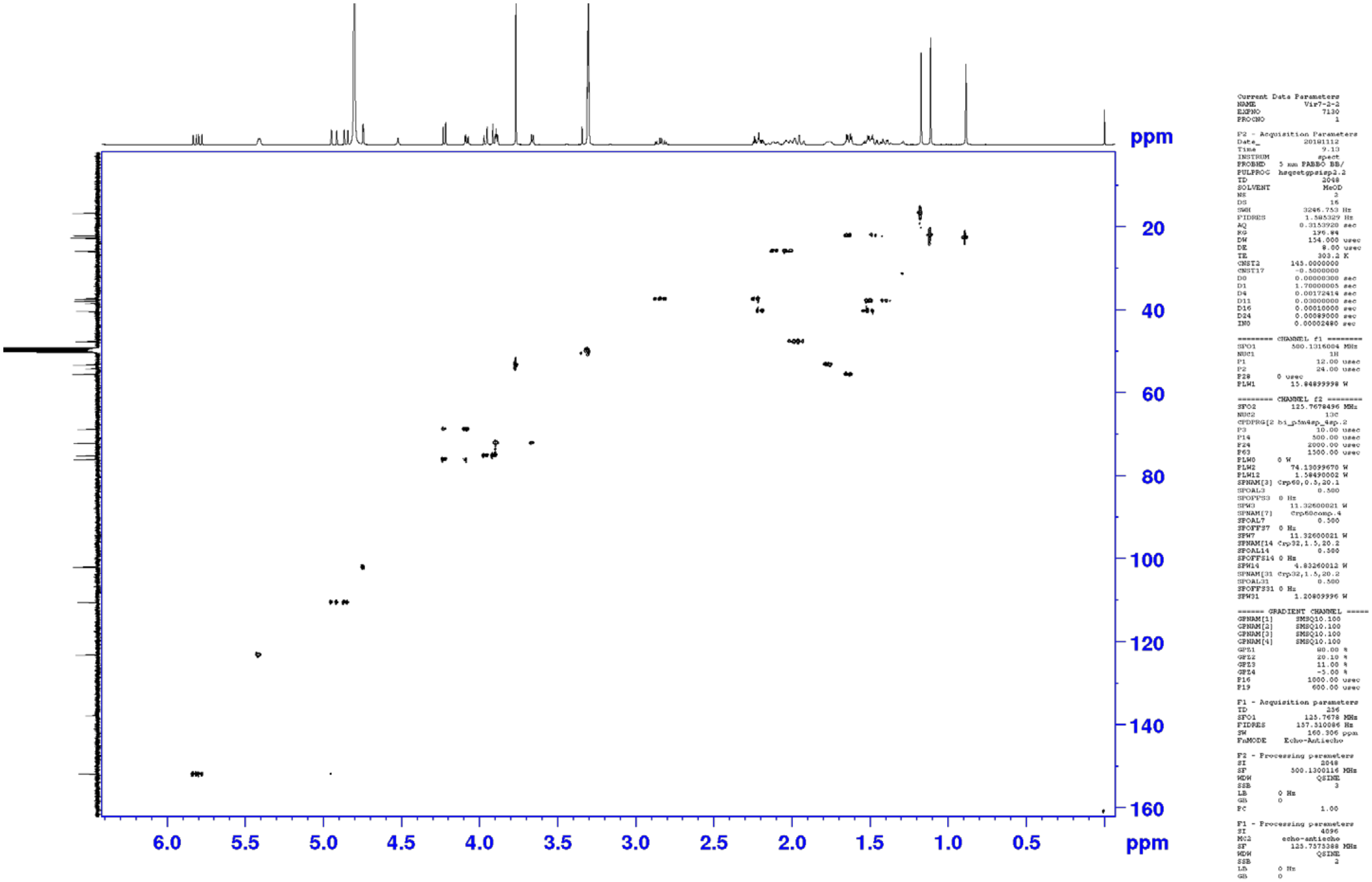

Figure S41. HSQC spectrum (500 MHz, CD_3OD) of **8**

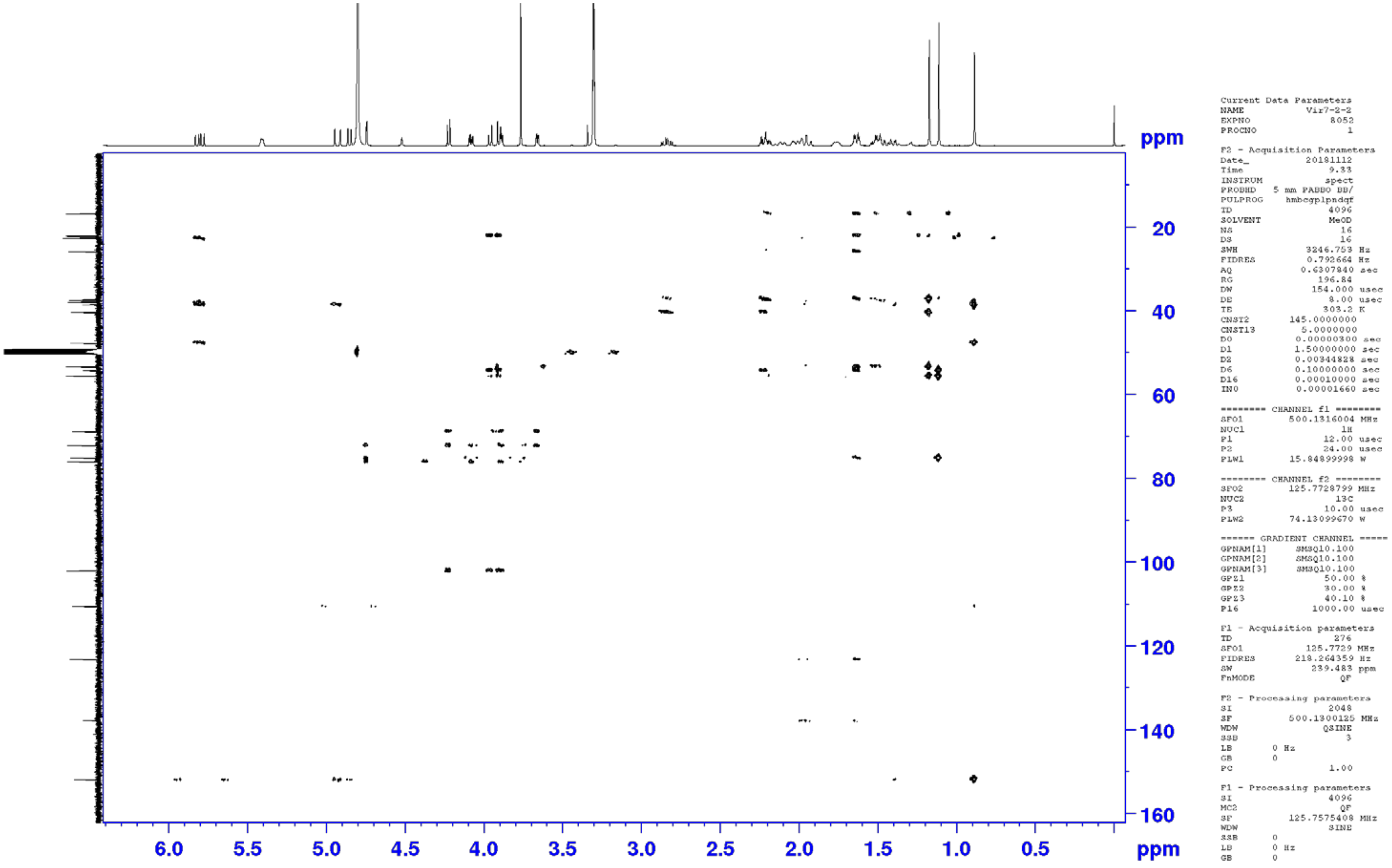

Figure S42. HMBC spectrum (500 MHz, CD_3OD) of **8**

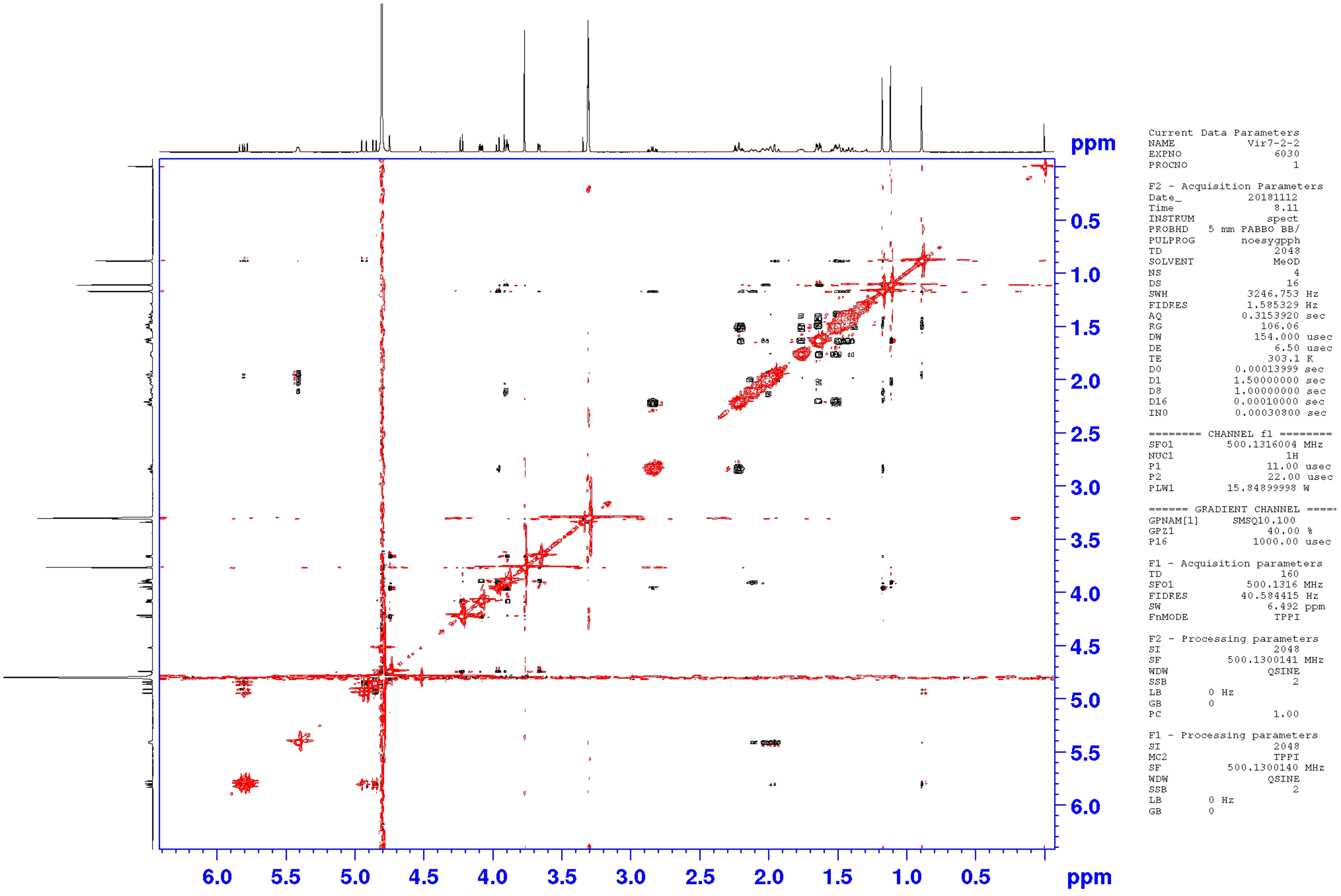

Figure S43. NOESY spectrum (500 MHz, CD_3OD) of **8**

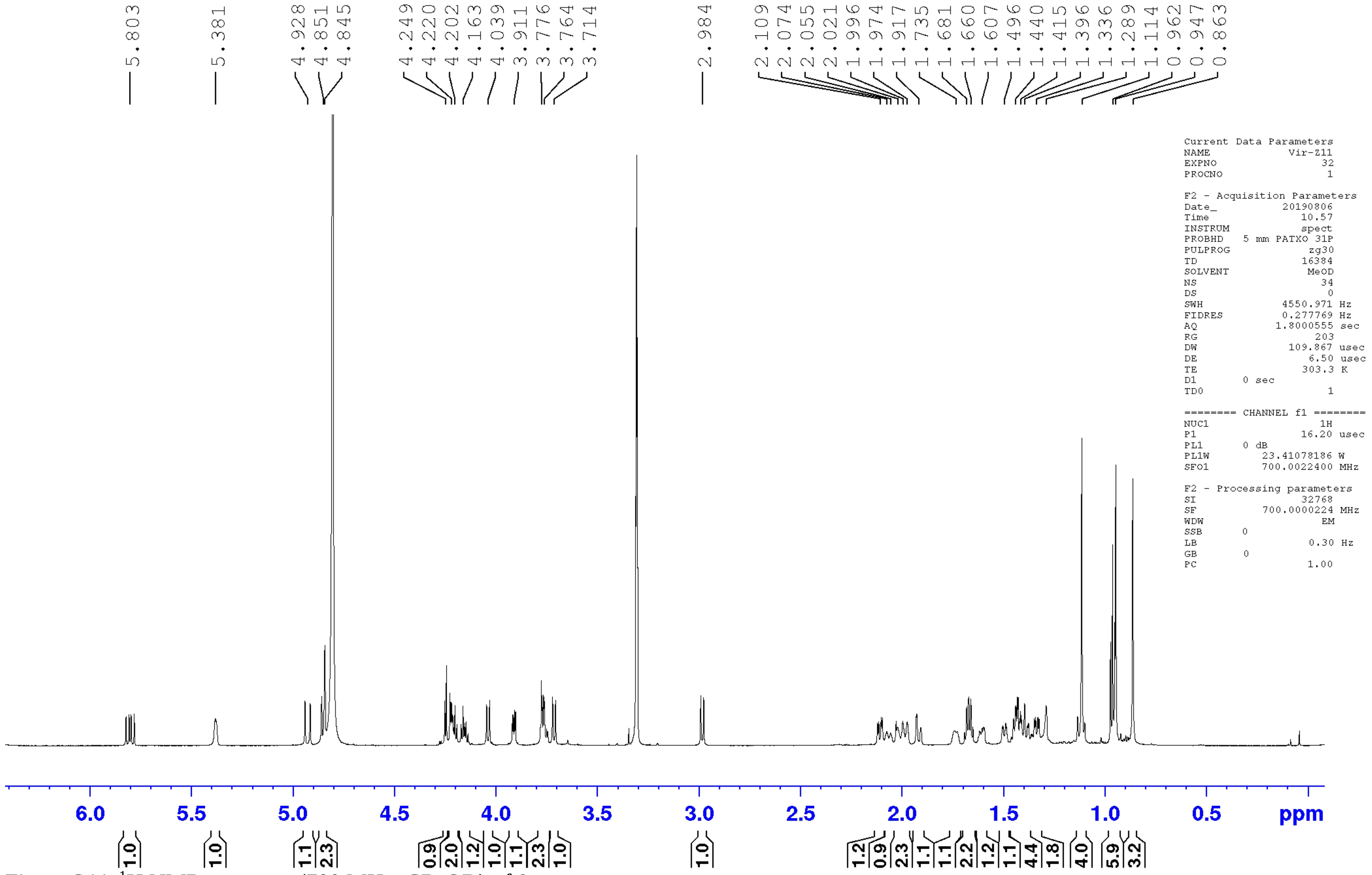

Figure S44. ^{1}H NMR spectrum (700 MHz, CD_3OD) of **9**

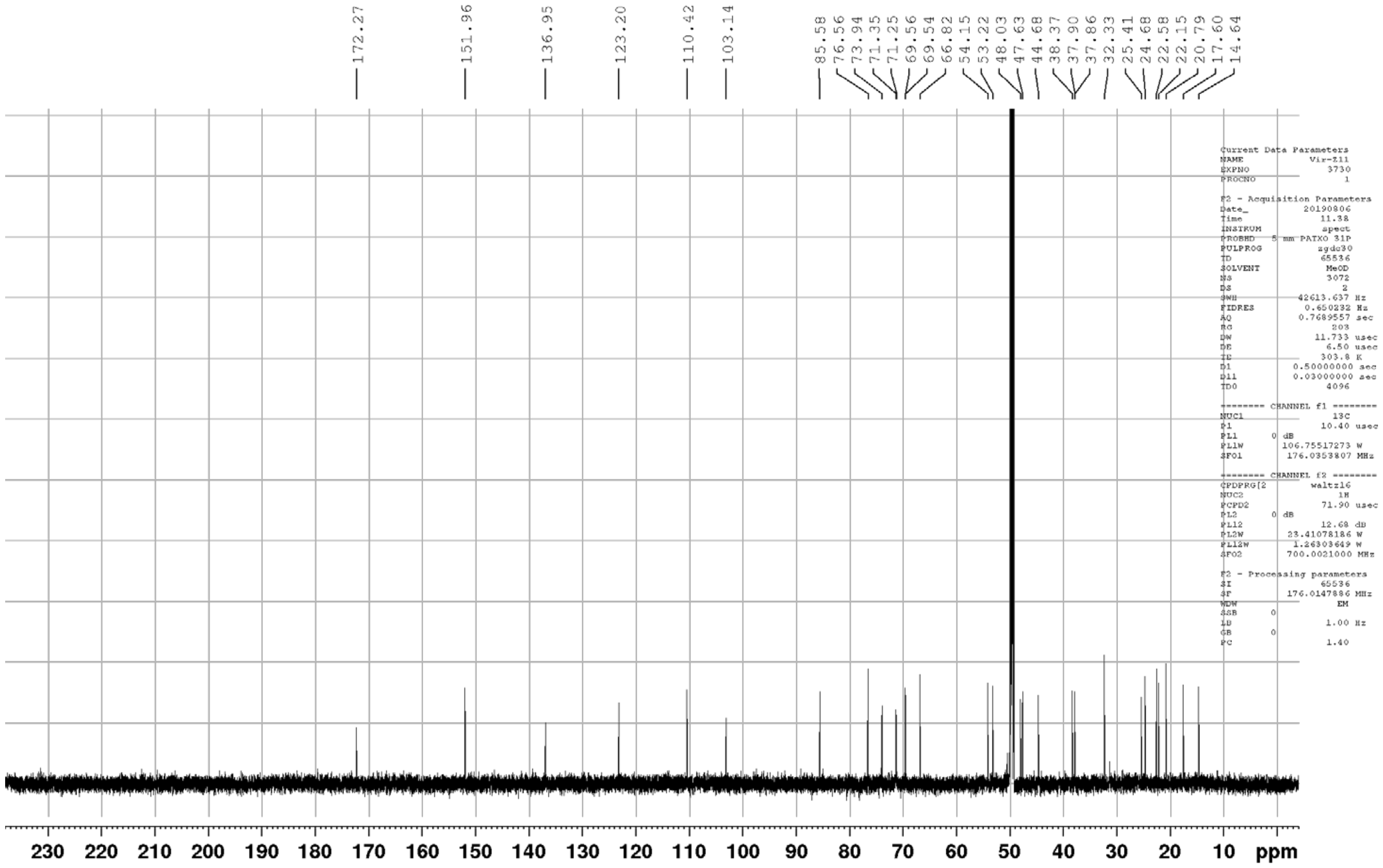

Figure S45. ^{13}C NMR spectrum (176 MHz, CD_3OD) of **9**

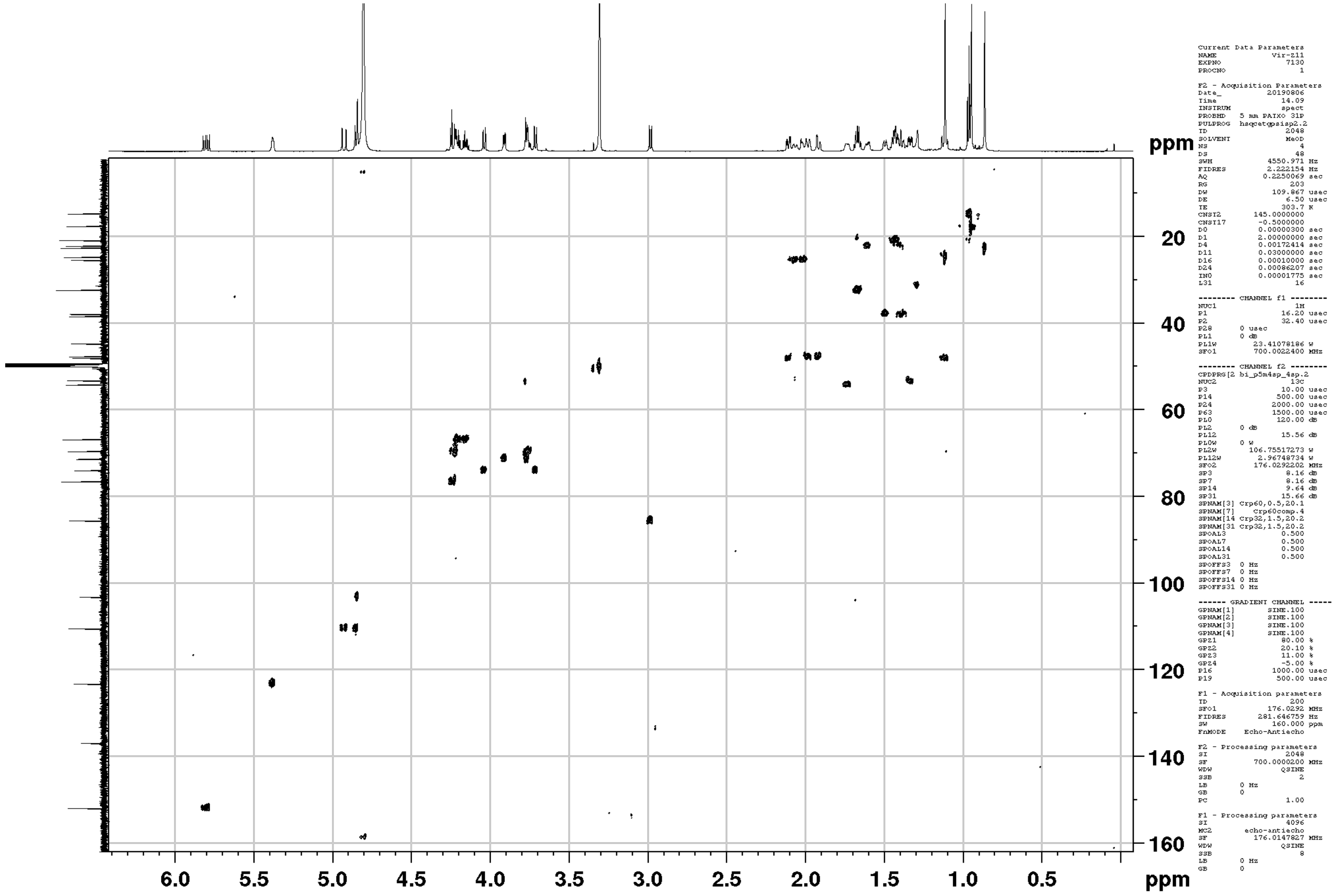

Figure S46. HSQC spectrum (700 MHz, CD_3OD) of **9**

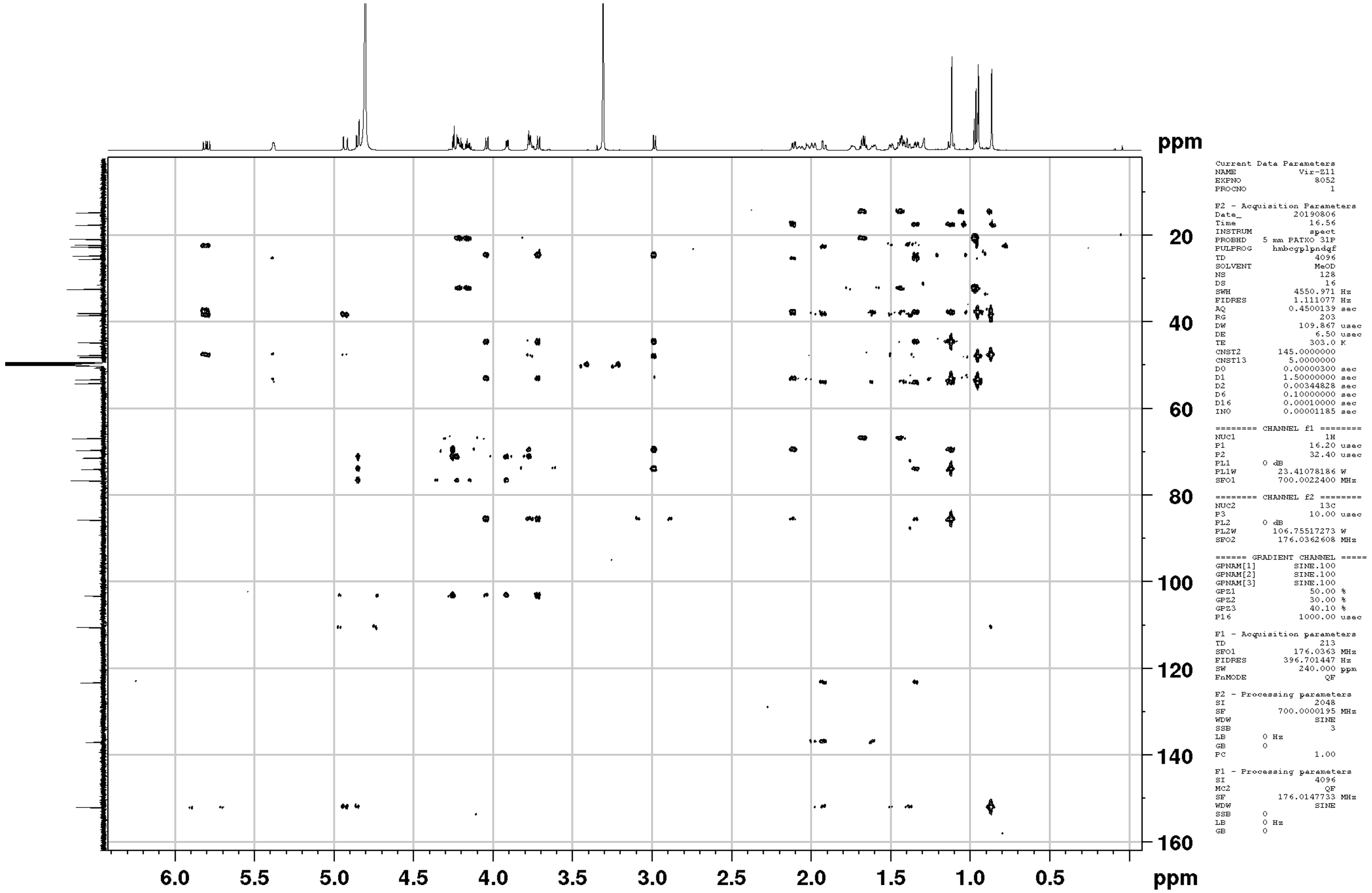

Figure S47. HMBC spectrum (700 MHz, CD_3OD) of **9**

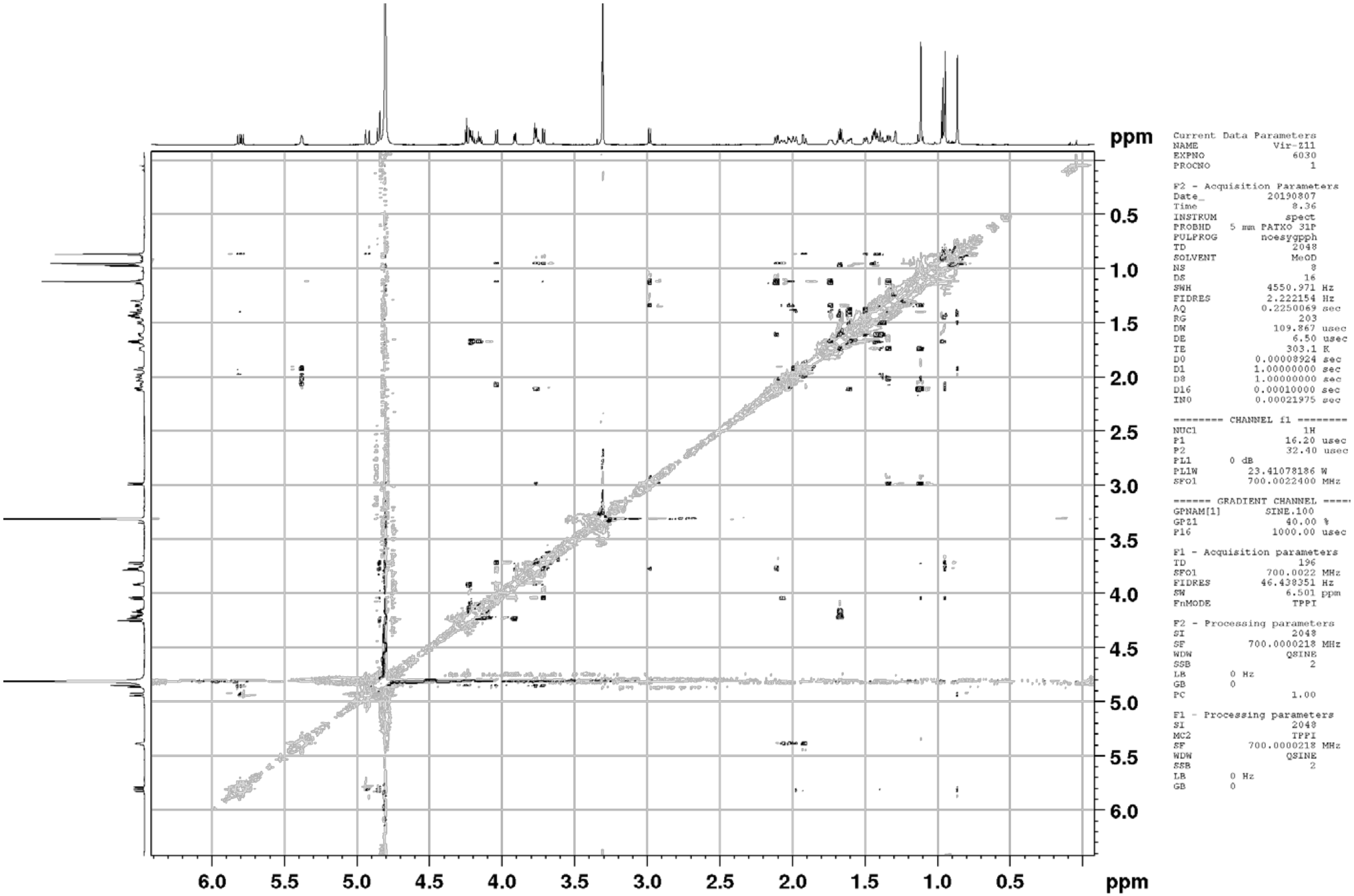

Figure S48. NOESY spectrum (700 MHz, CD_3OD) of **9**

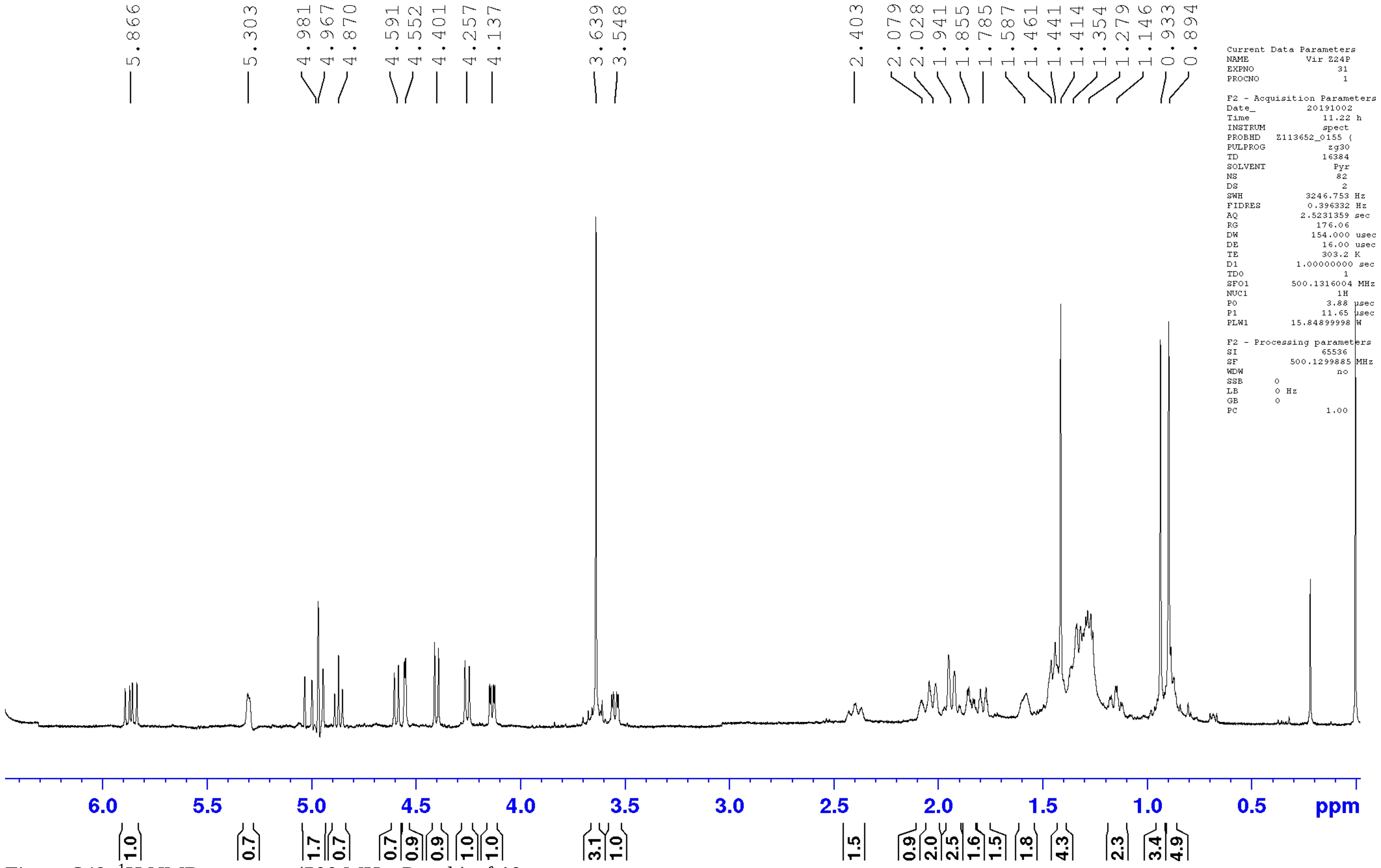

Figure S49. ^{1}H NMR spectrum (500 MHz, Pyr-d_5) of **10**

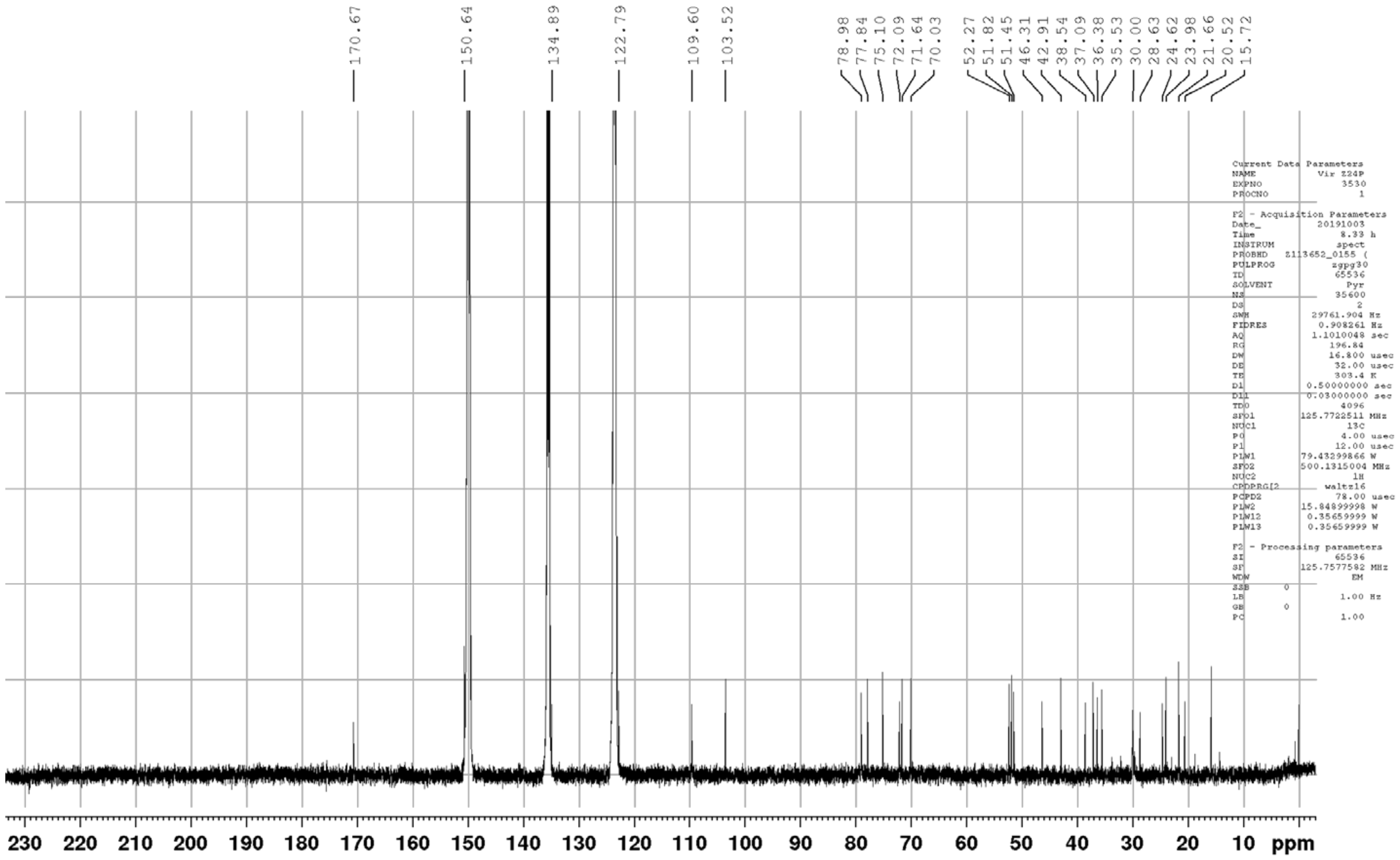

Figure S50. ^{13}C NMR spectrum (125 MHz, Pyr-d_5) of **10**

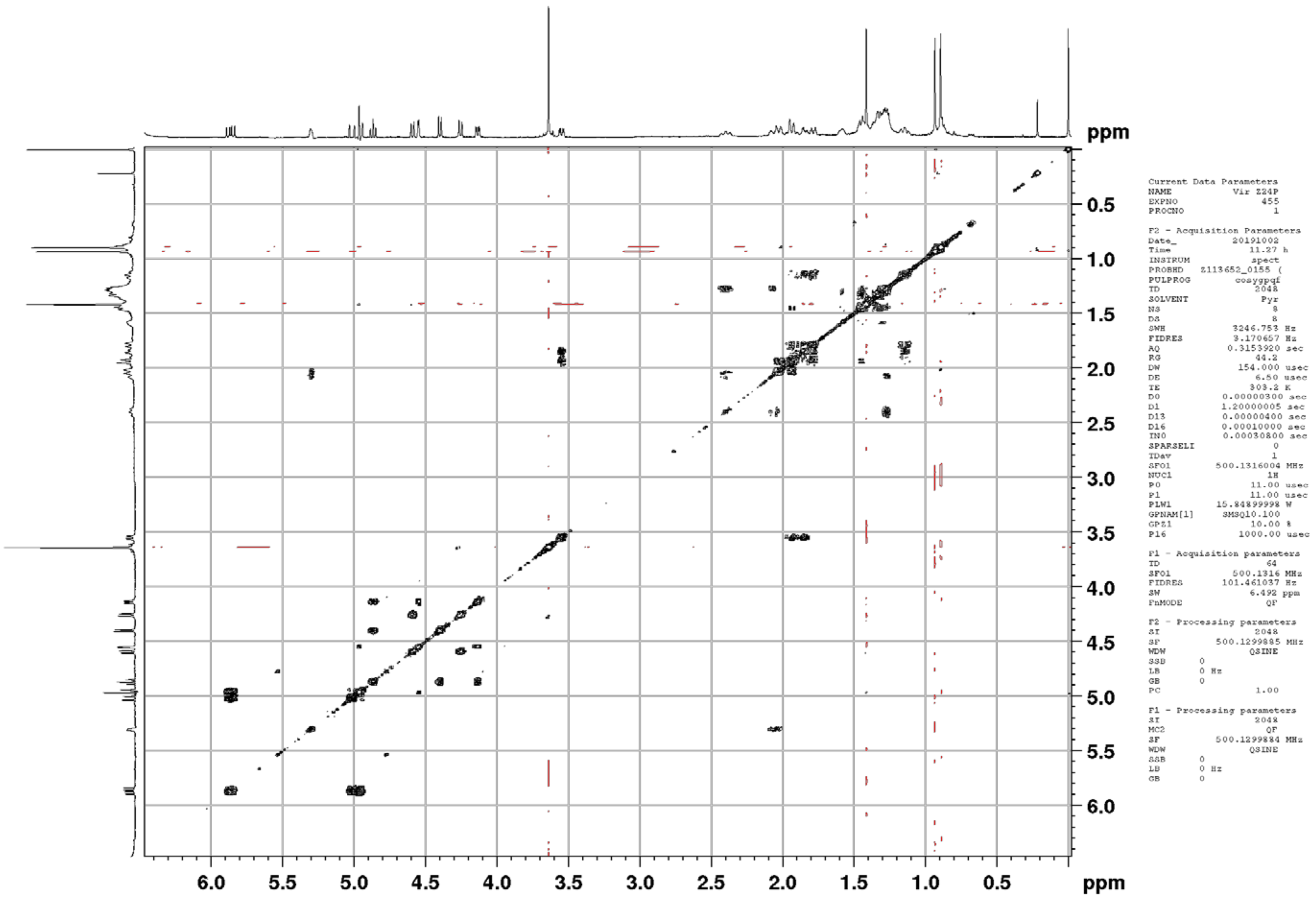

Figure S51. COSY-45 spectrum (500 MHz, Pyr-d_5) of **10**

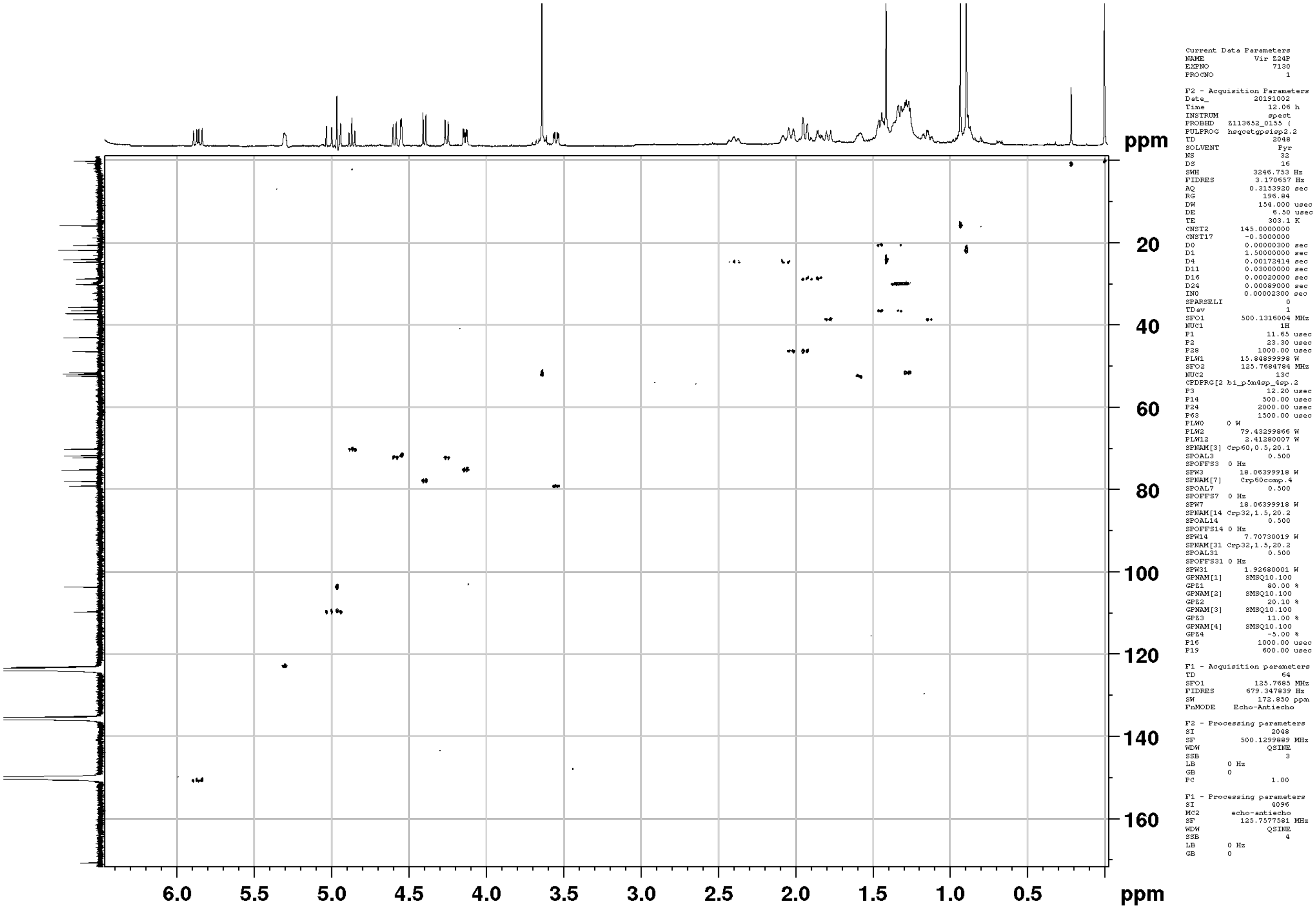

Figure S52. HSQC spectrum (500 MHz, Pyr-d_5) of **10**

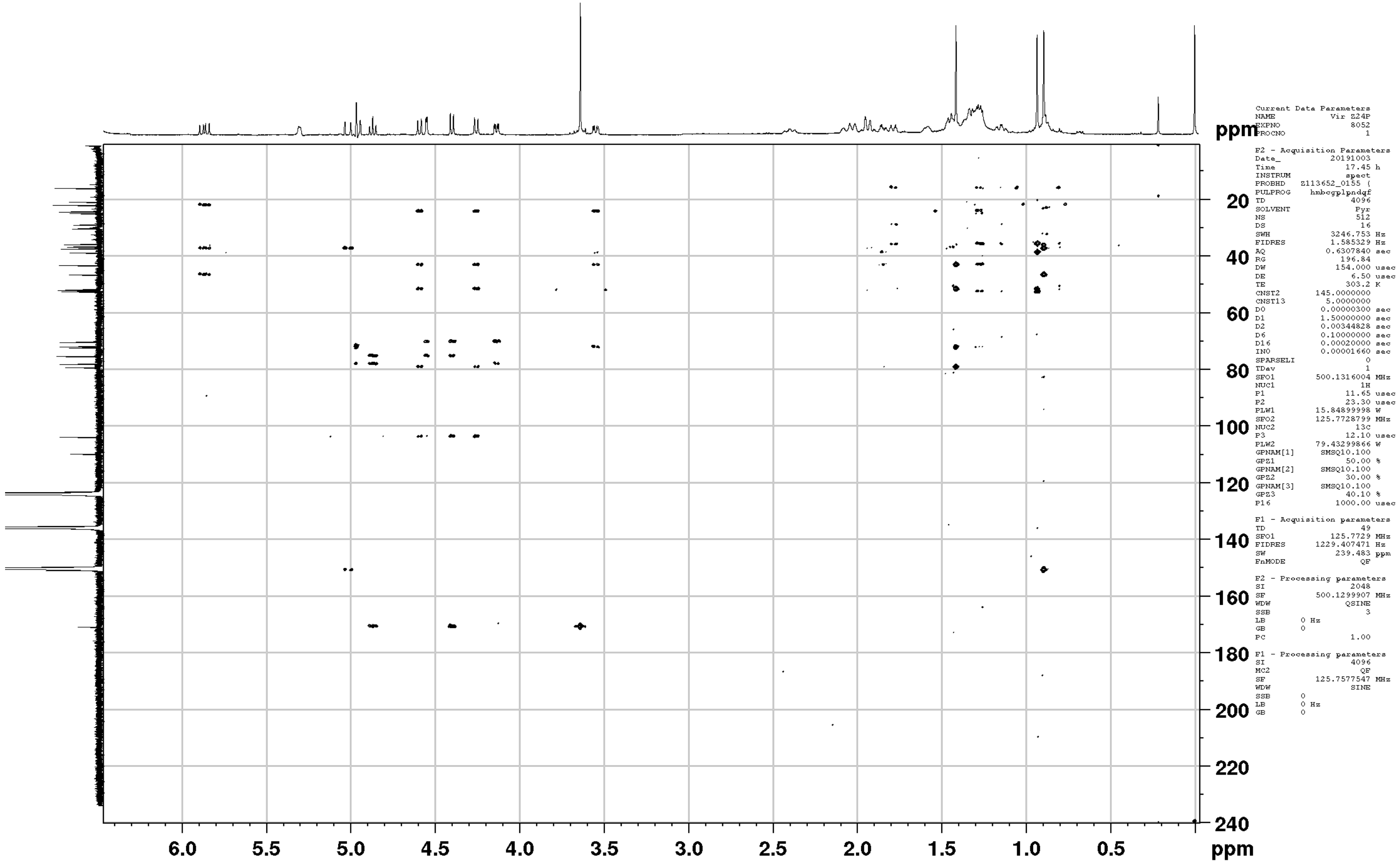

Figure S53. HMBC spectrum (500 MHz, Pyr-d_5) of **10**

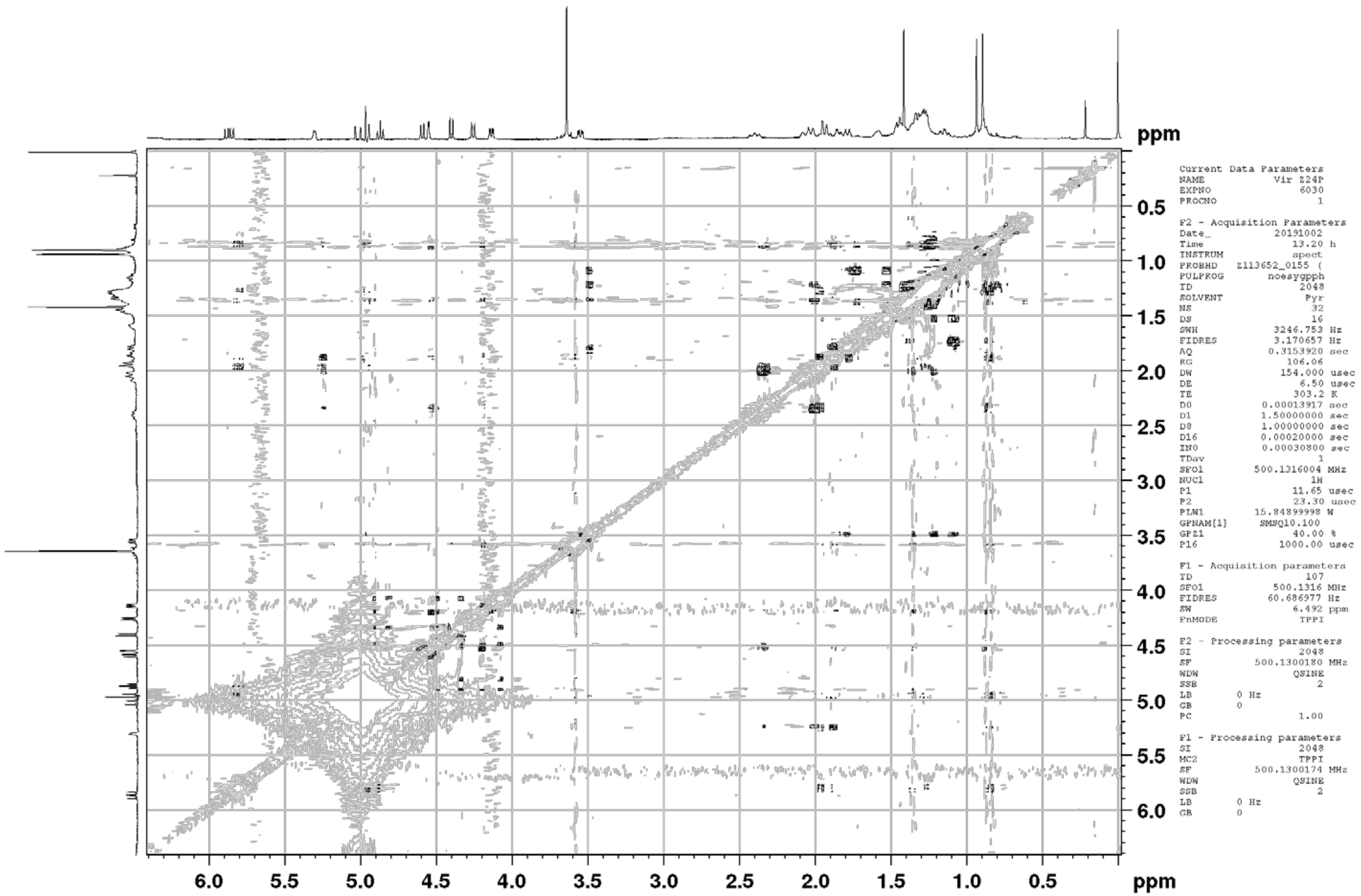

Figure S54. NOESY spectrum (500 MHz, Pyr-d_5) of **10**

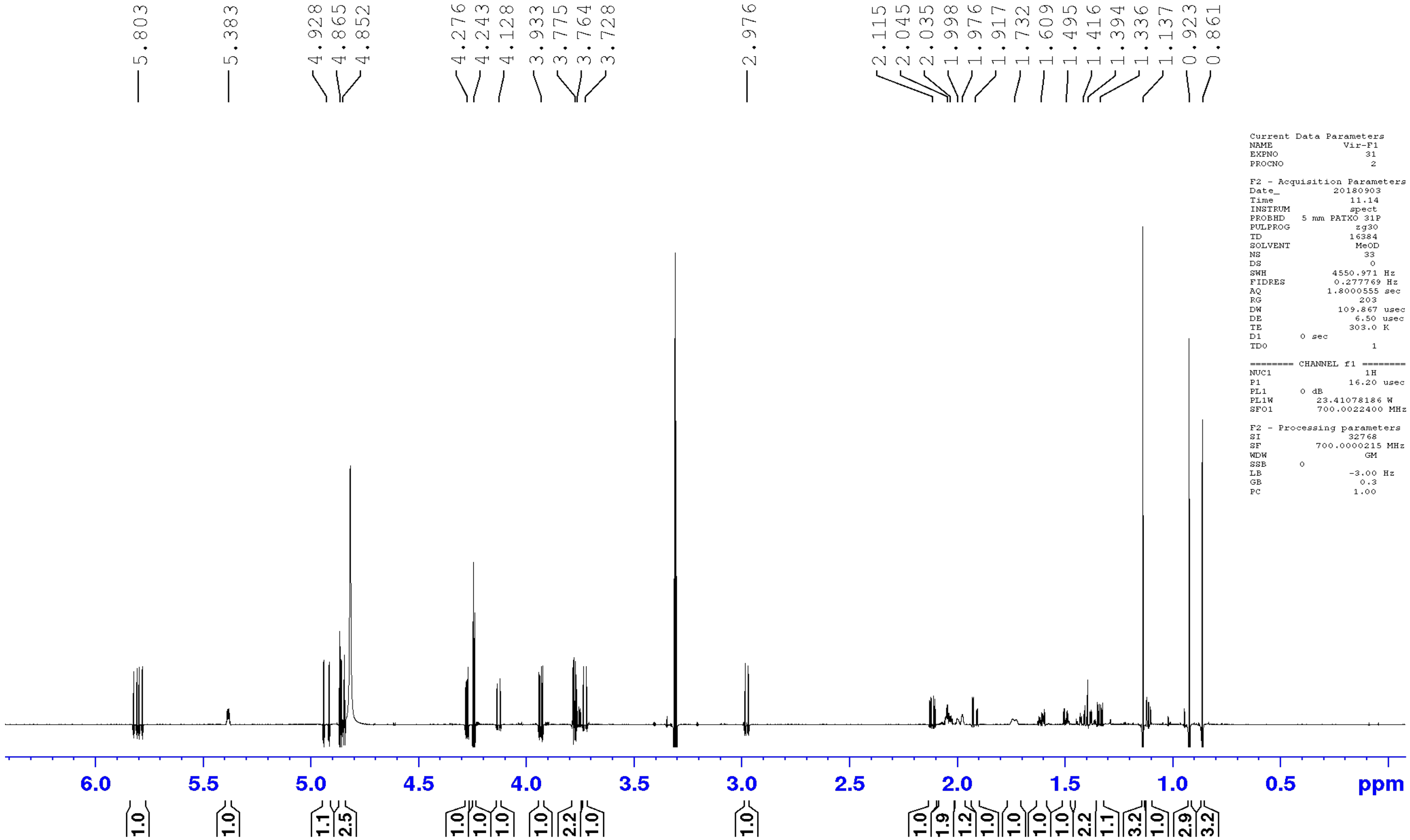

Figure S55. ^{1}H NMR spectrum (700 MHz, CD_3OD) of **11**

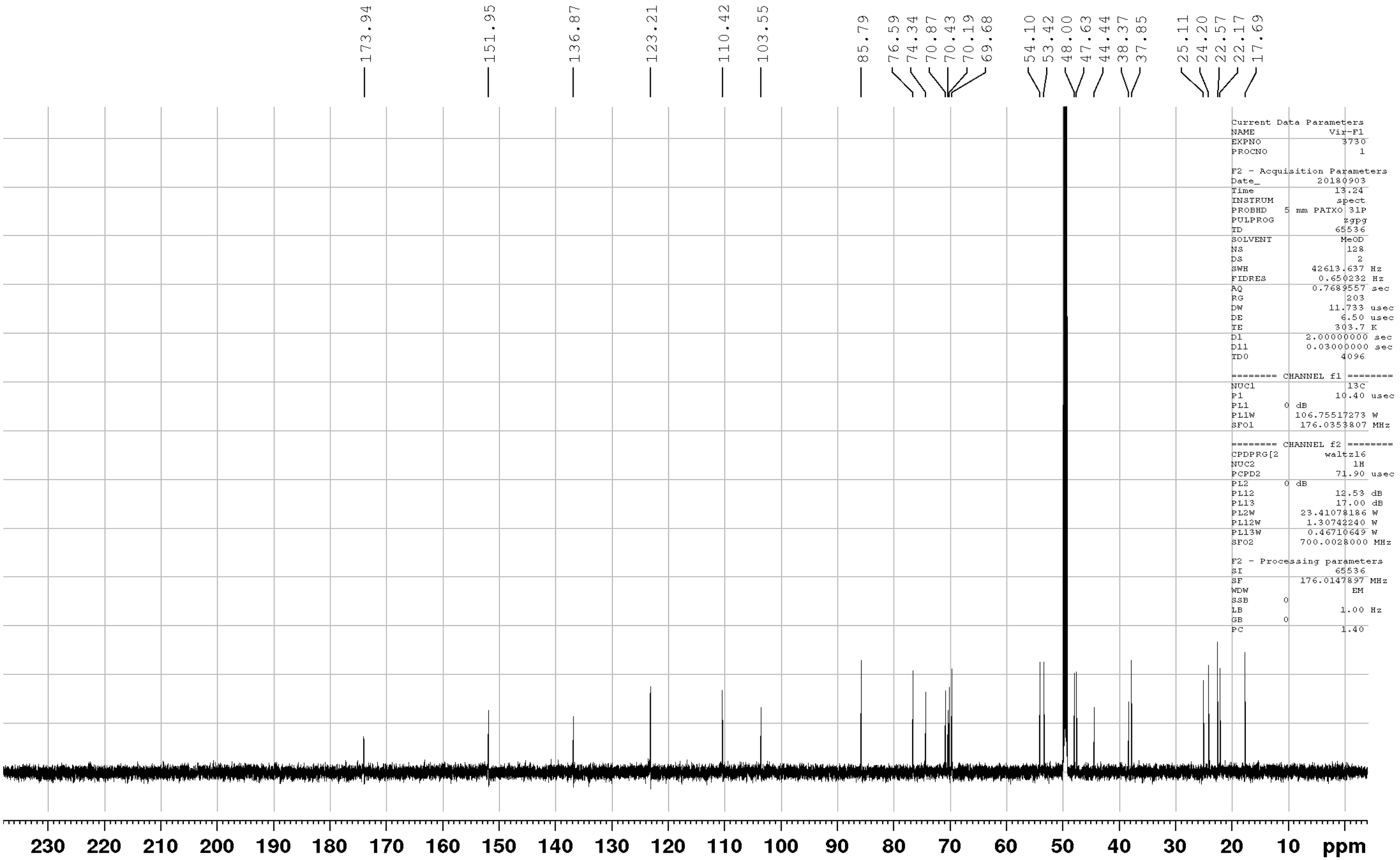

Figure S56. ^{13}C NMR spectrum (176 MHz, CD_3OD) of **11**

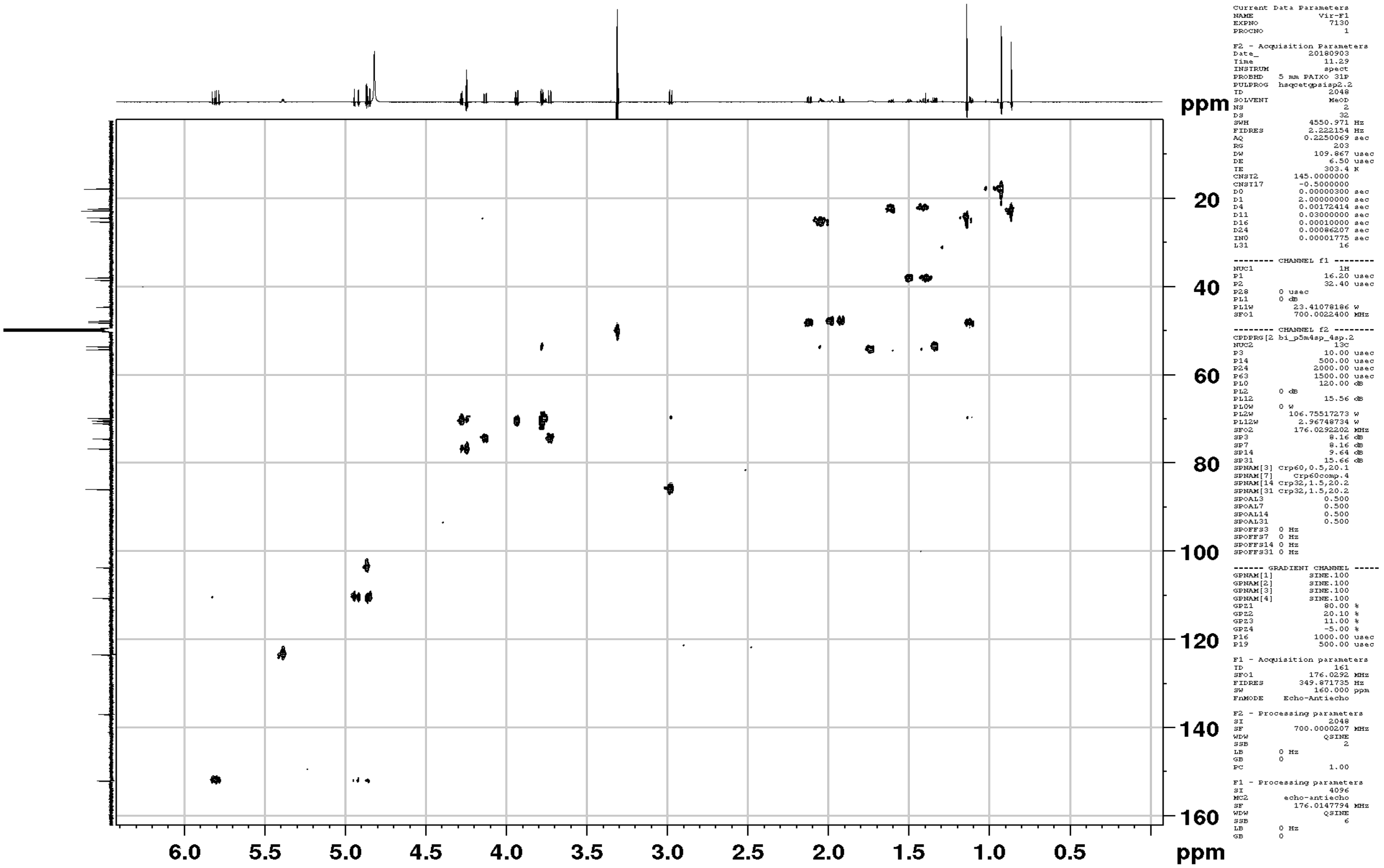

Figure S57. HSQC spectrum (700 MHz, CD_3OD) of **11**

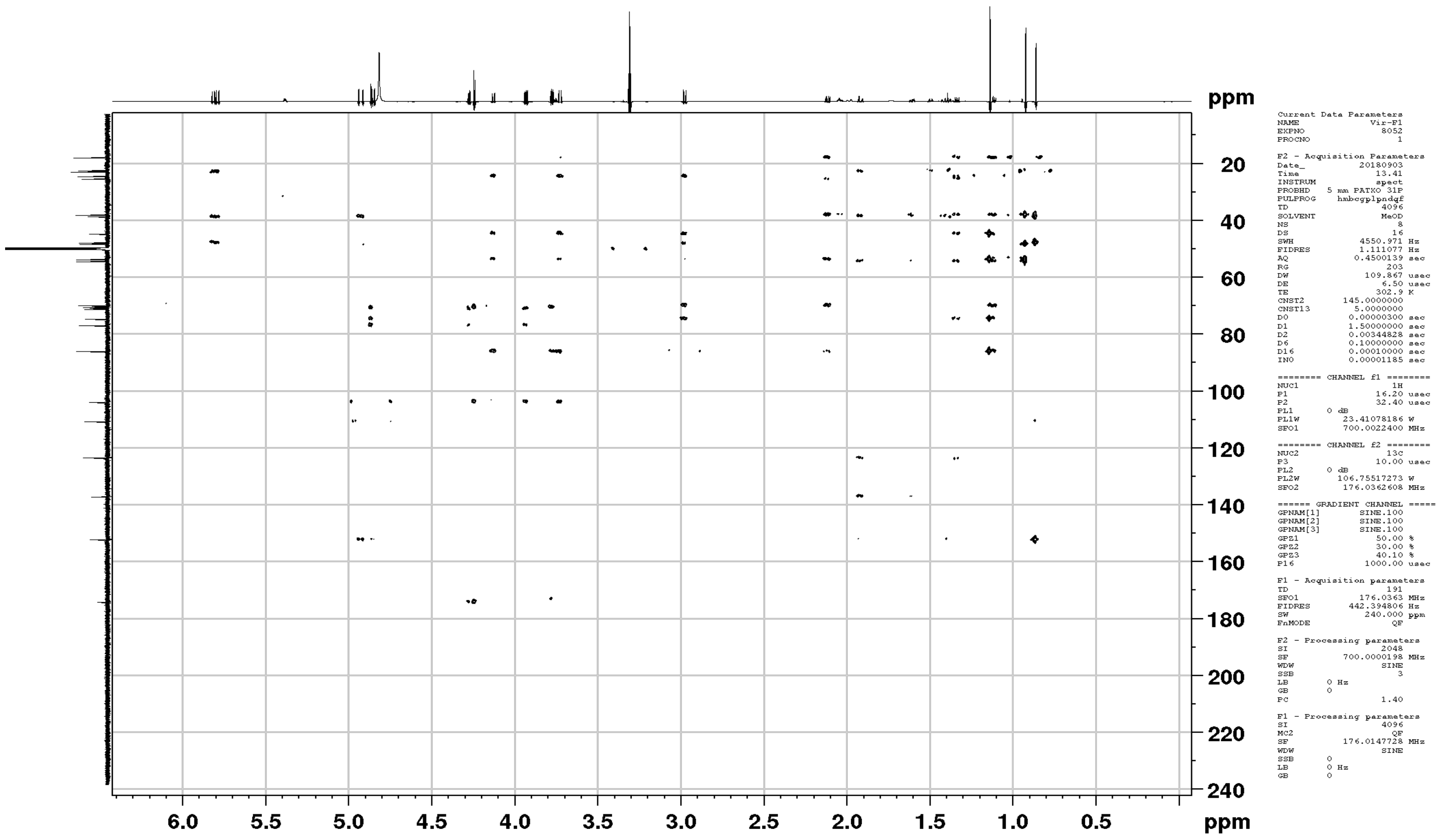

Figure S58. HMBC spectrum (700 MHz, CD_3OD) of **11**

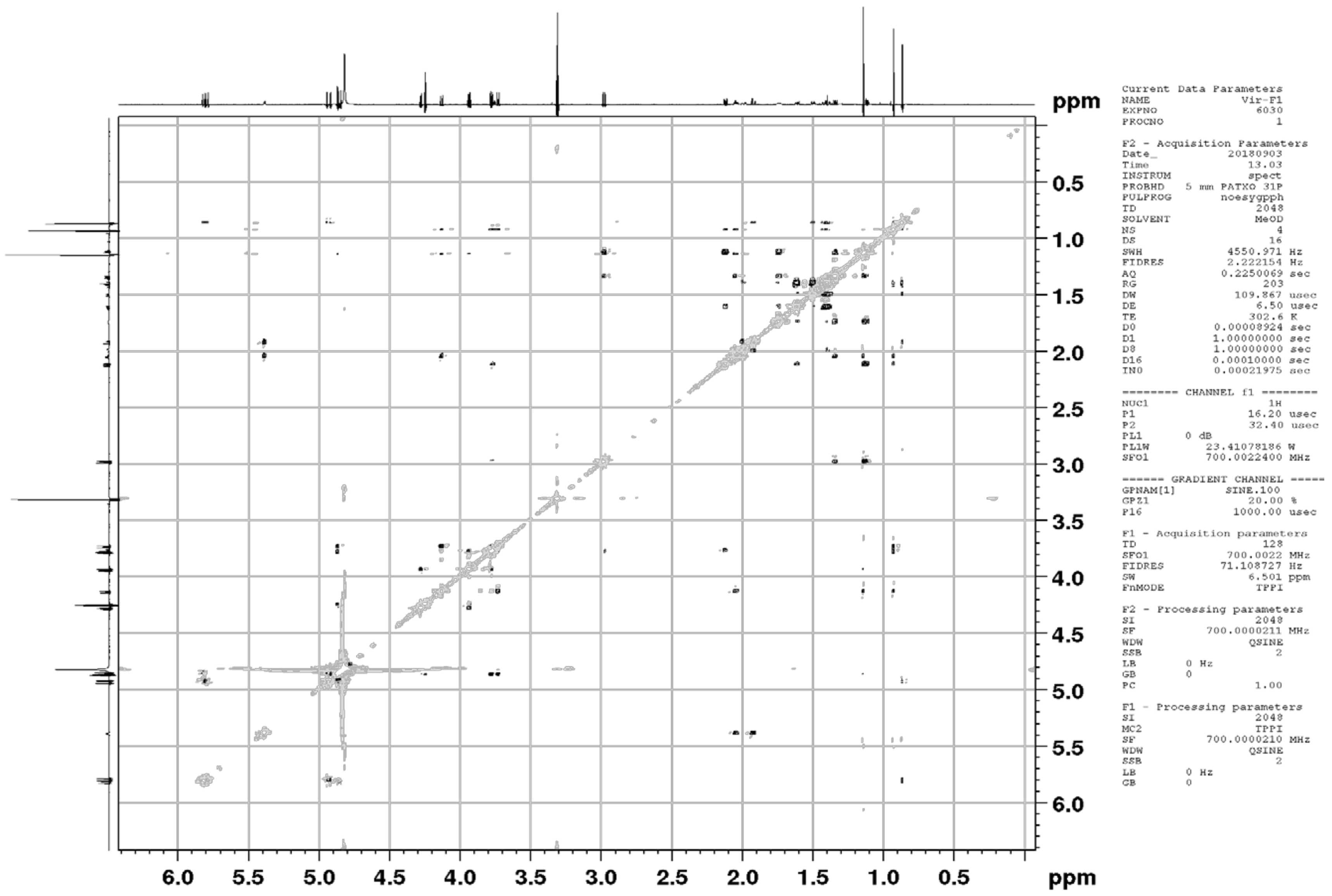

Figure S59. NOESY spectrum (700 MHz, CD_3OD) of **11**

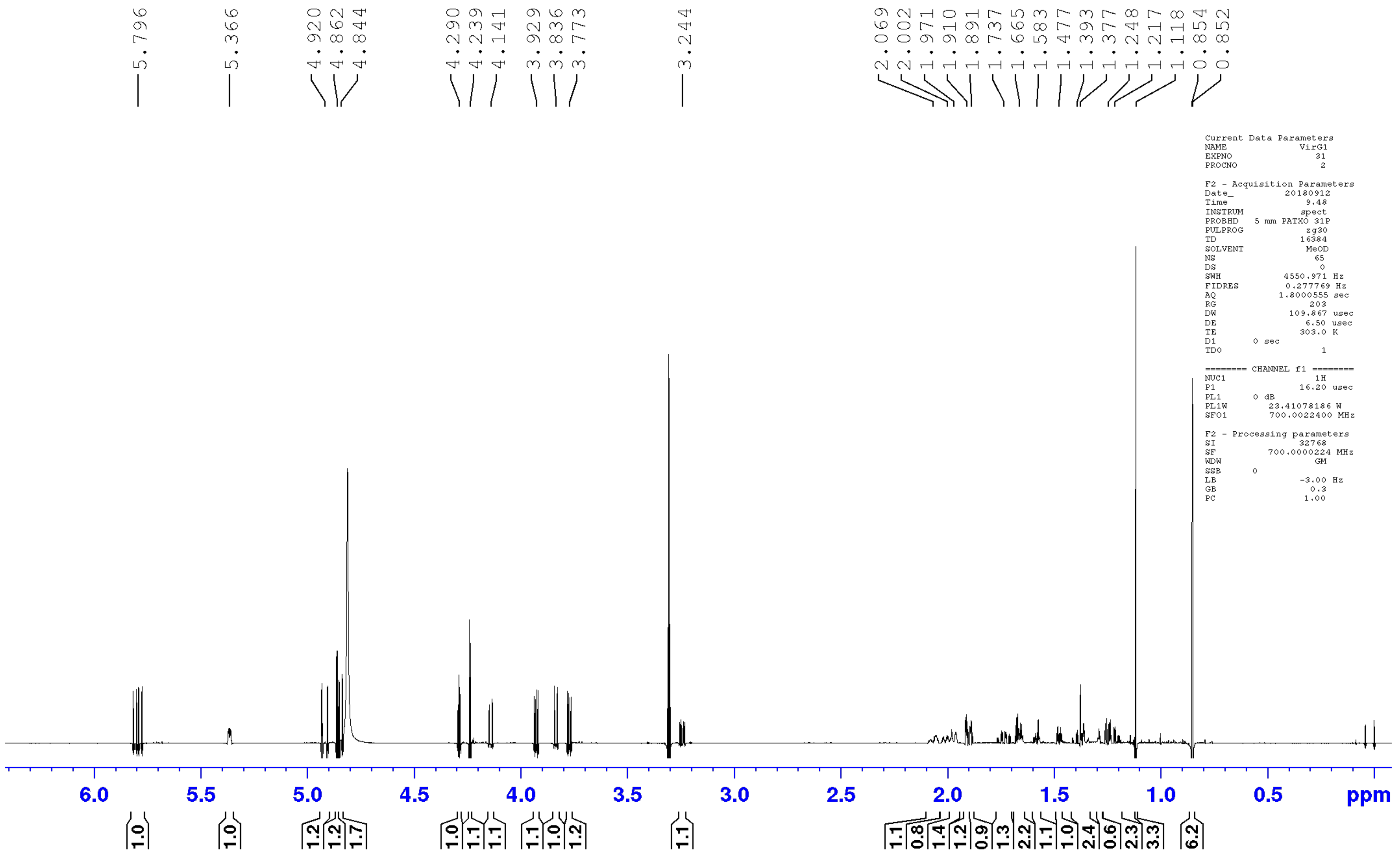

Figure S60. ^{1}H NMR spectrum (700 MHz, CD_3OD) of **12**

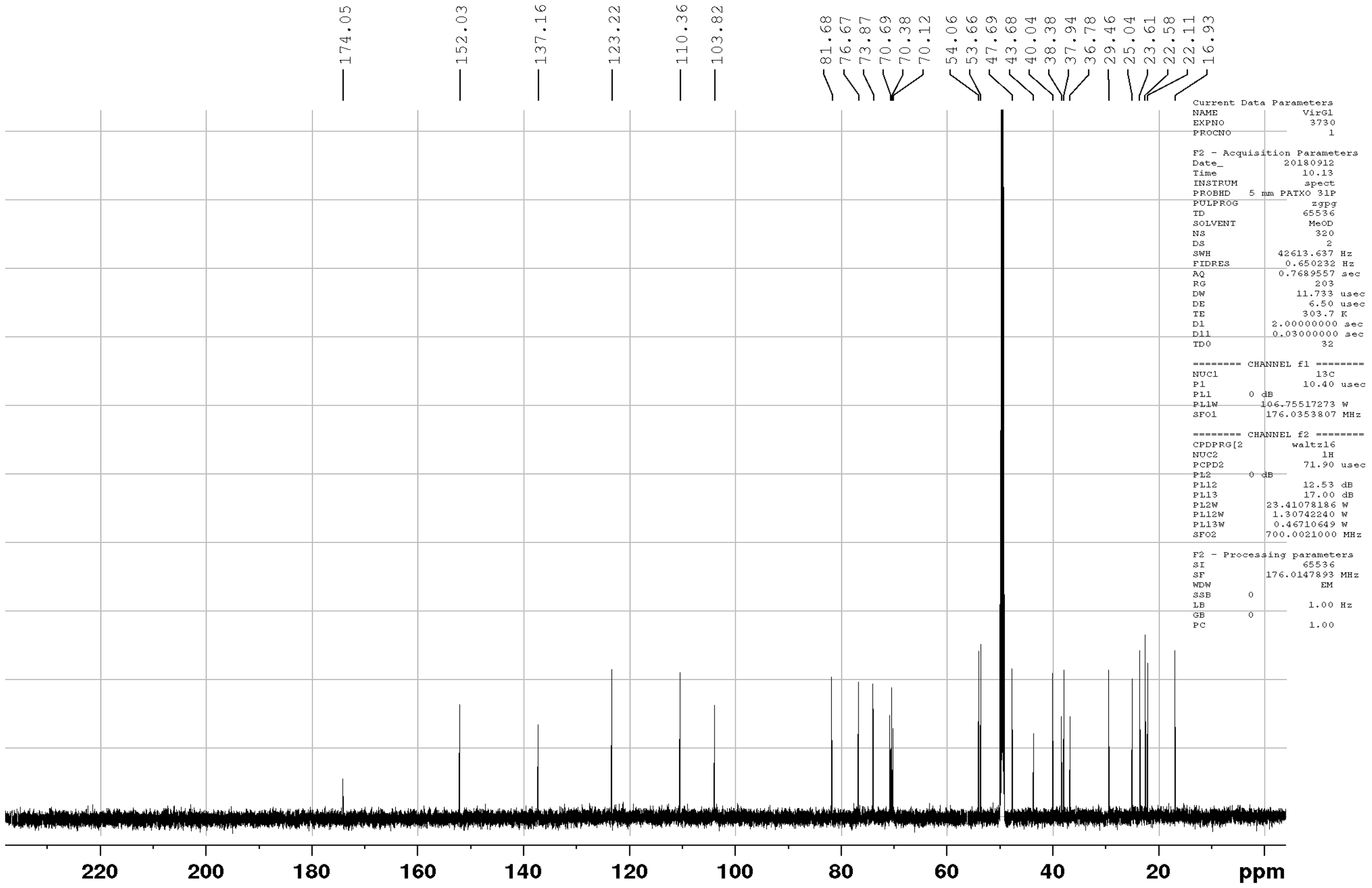

Figure S61. ^{13}C NMR spectrum (176 MHz, CD_3OD) of **12**

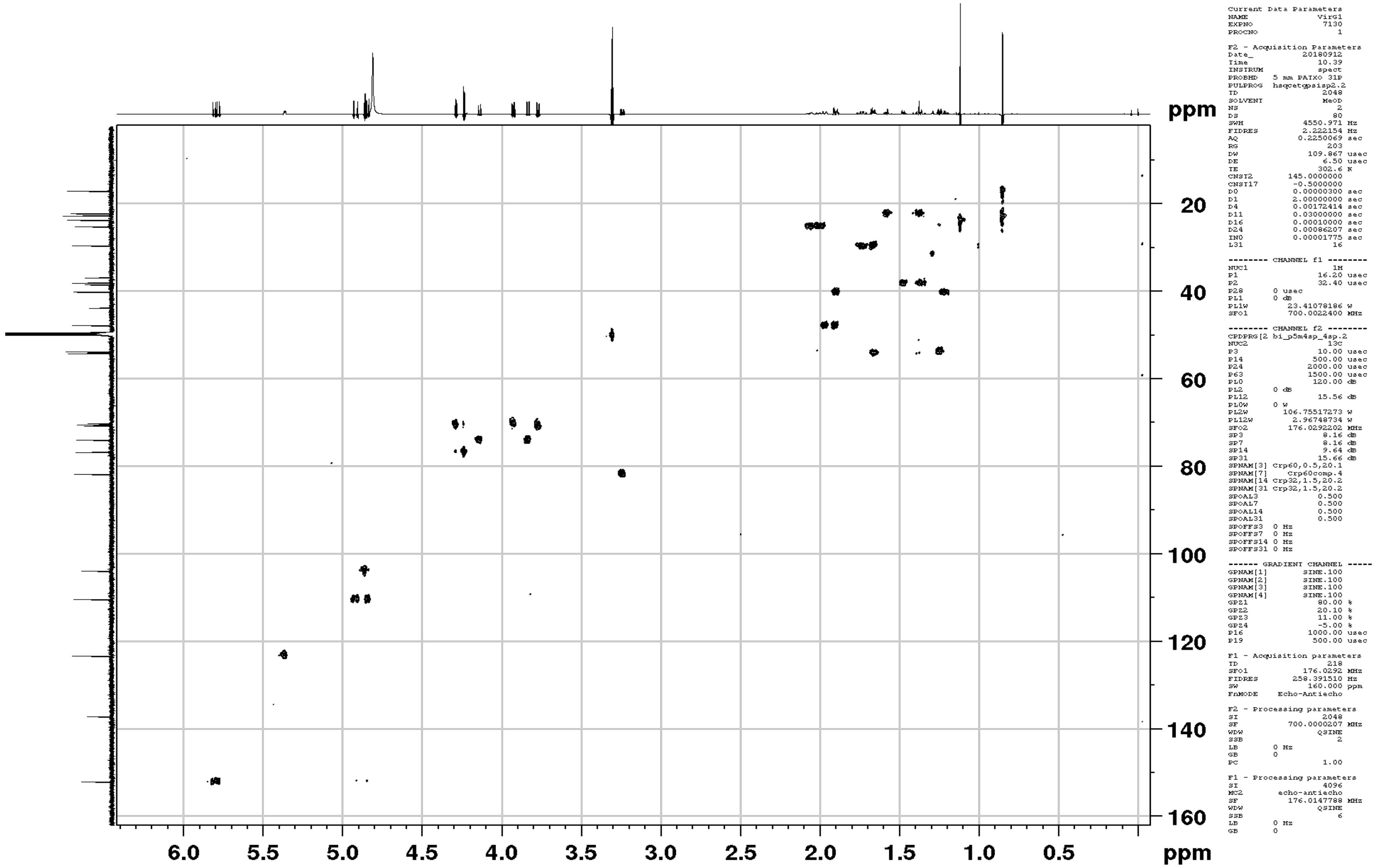

Figure S62. HSQC spectrum (700 MHz, CD_3OD) of **12**

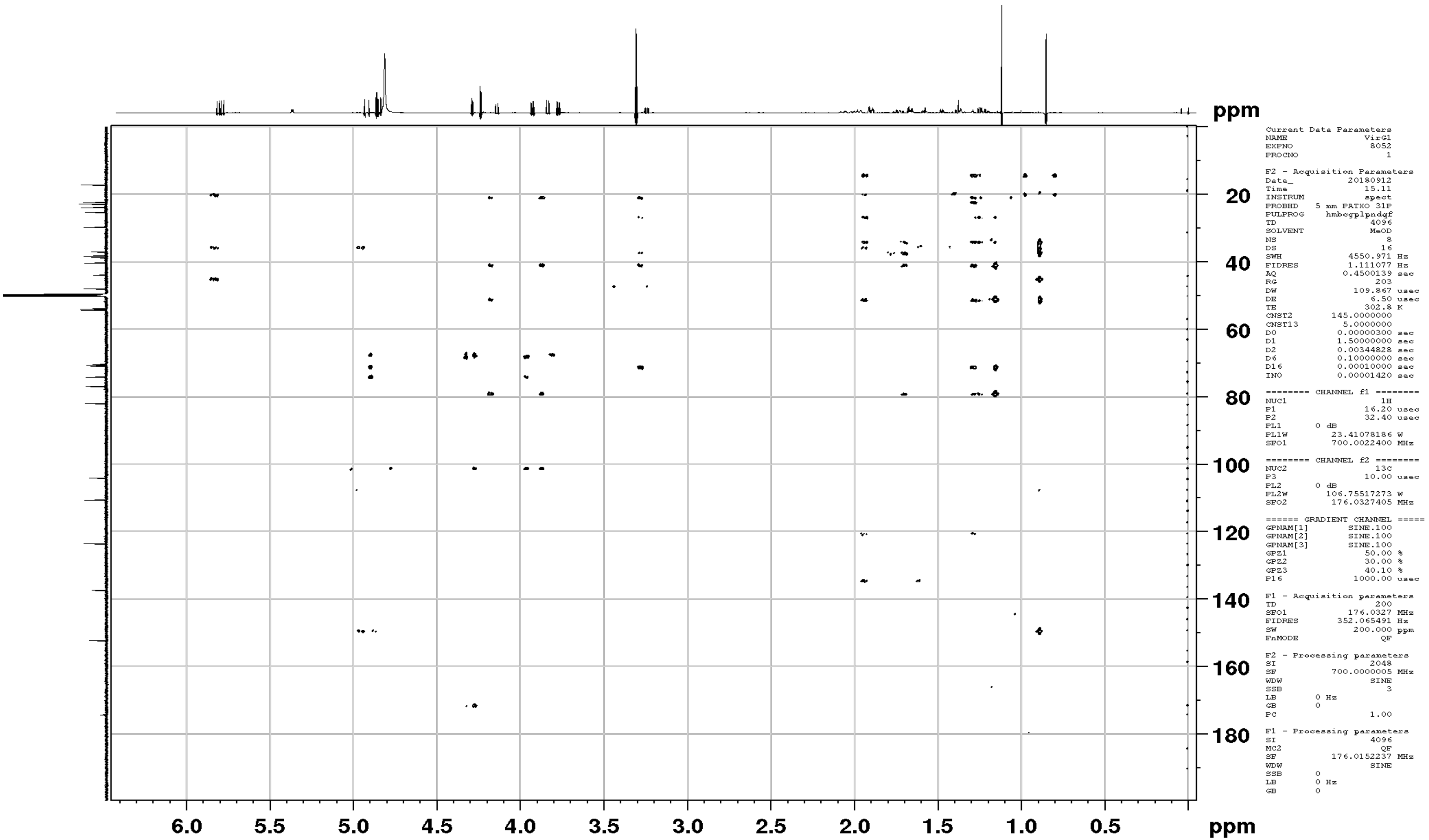

Figure S63. HMBC spectrum (700 MHz, CD_3OD) of **12**

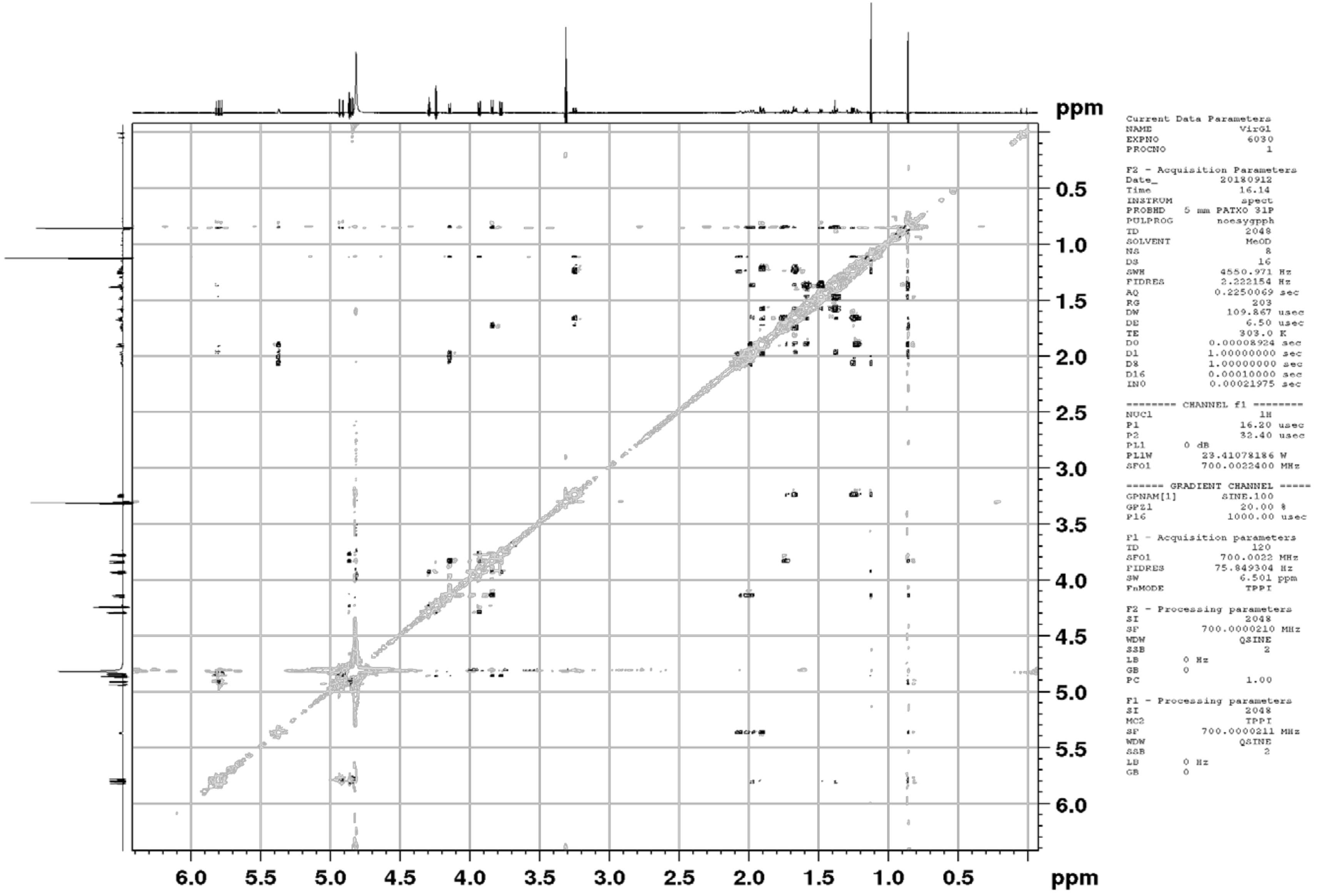

Figure S64. NOESY spectrum (700 MHz, CD_3OD) of **12**

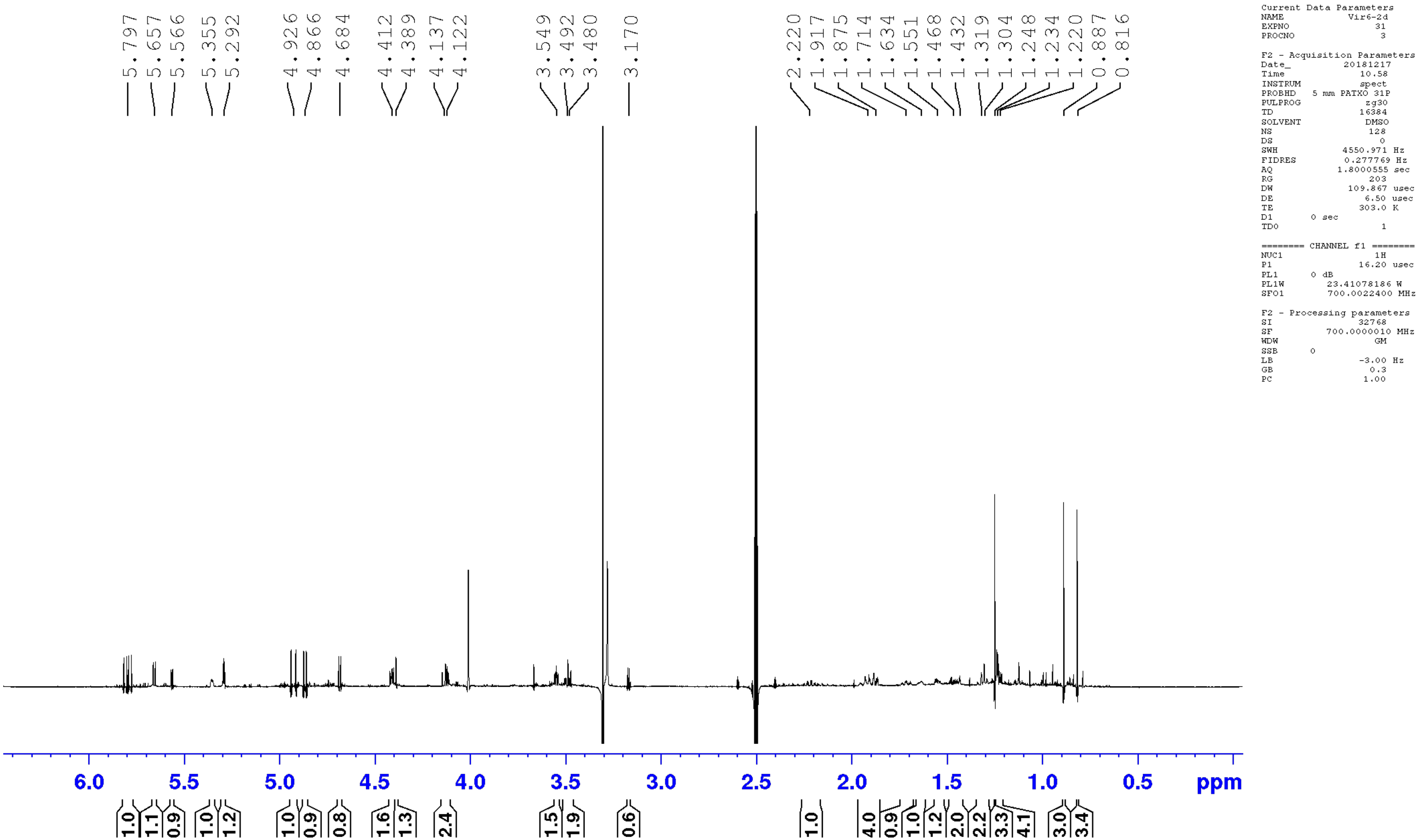

Figure S65. ^{1}H NMR spectrum (700 MHz, DMSO-d_6) of **13**

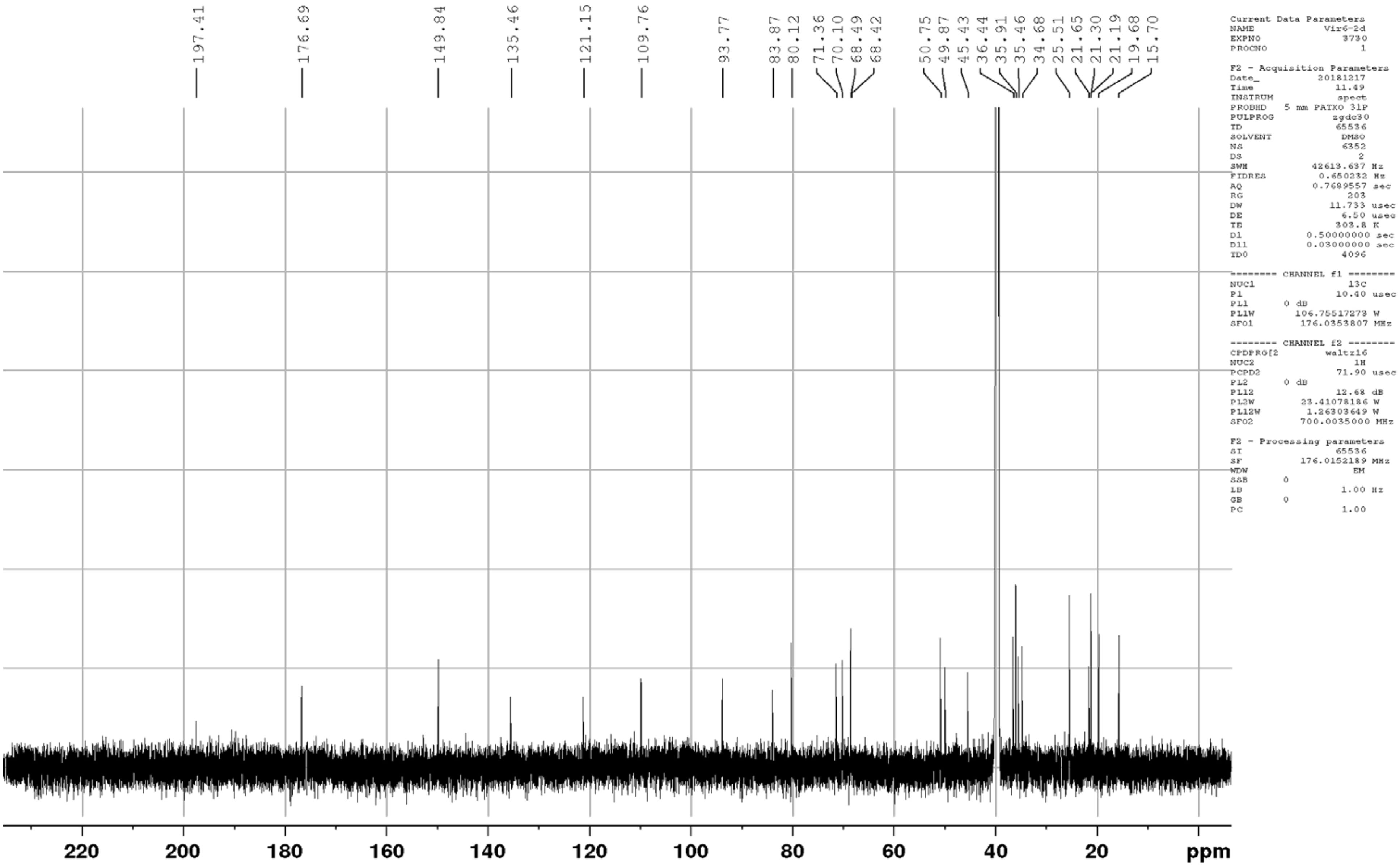

Figure S66. ^{13}C NMR spectrum (176 MHz, DMSO-d$_6$) of **13**

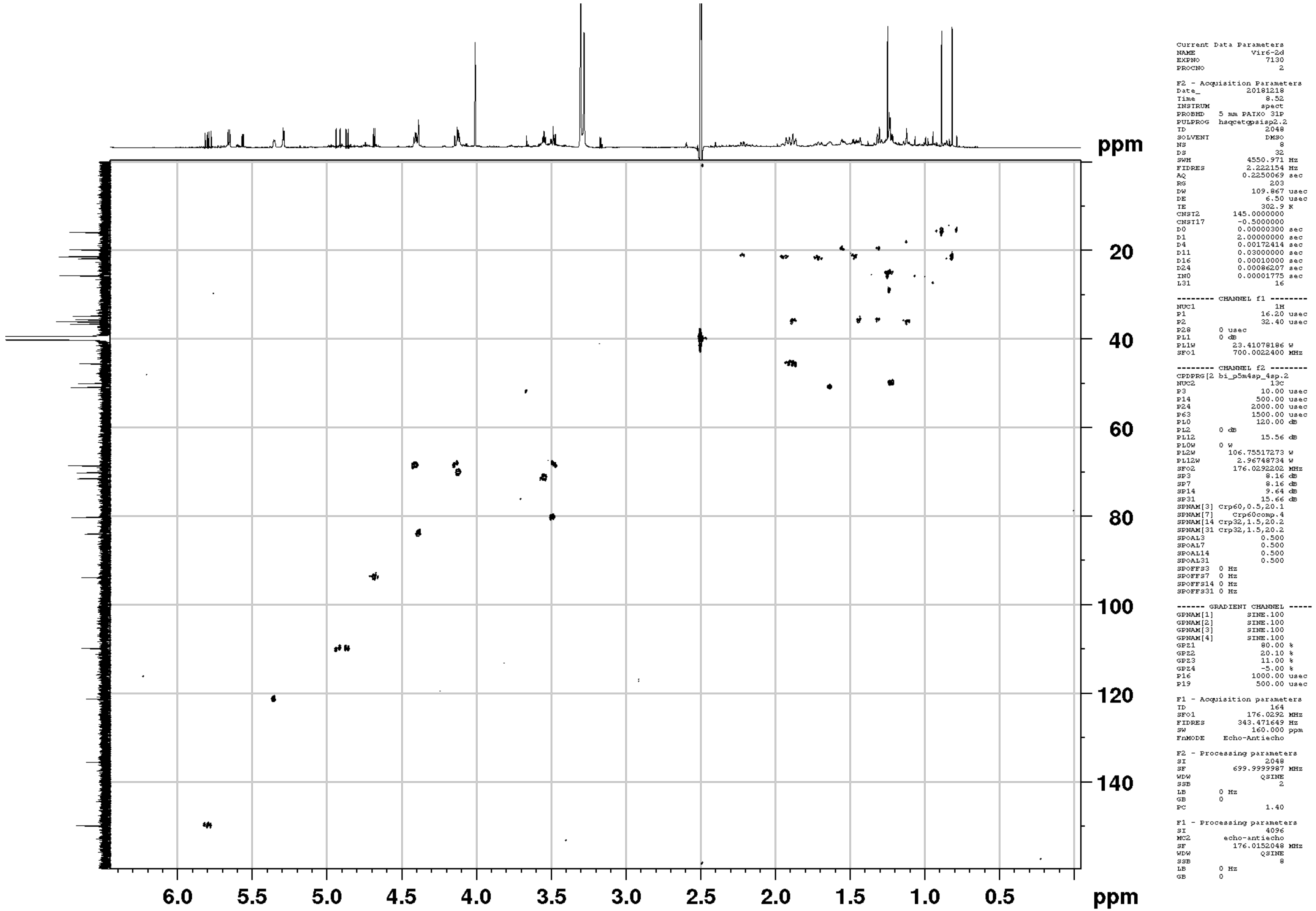

Figure S67. HSQC spectrum (700 MHz, DMSO-d$_6$) of **13**

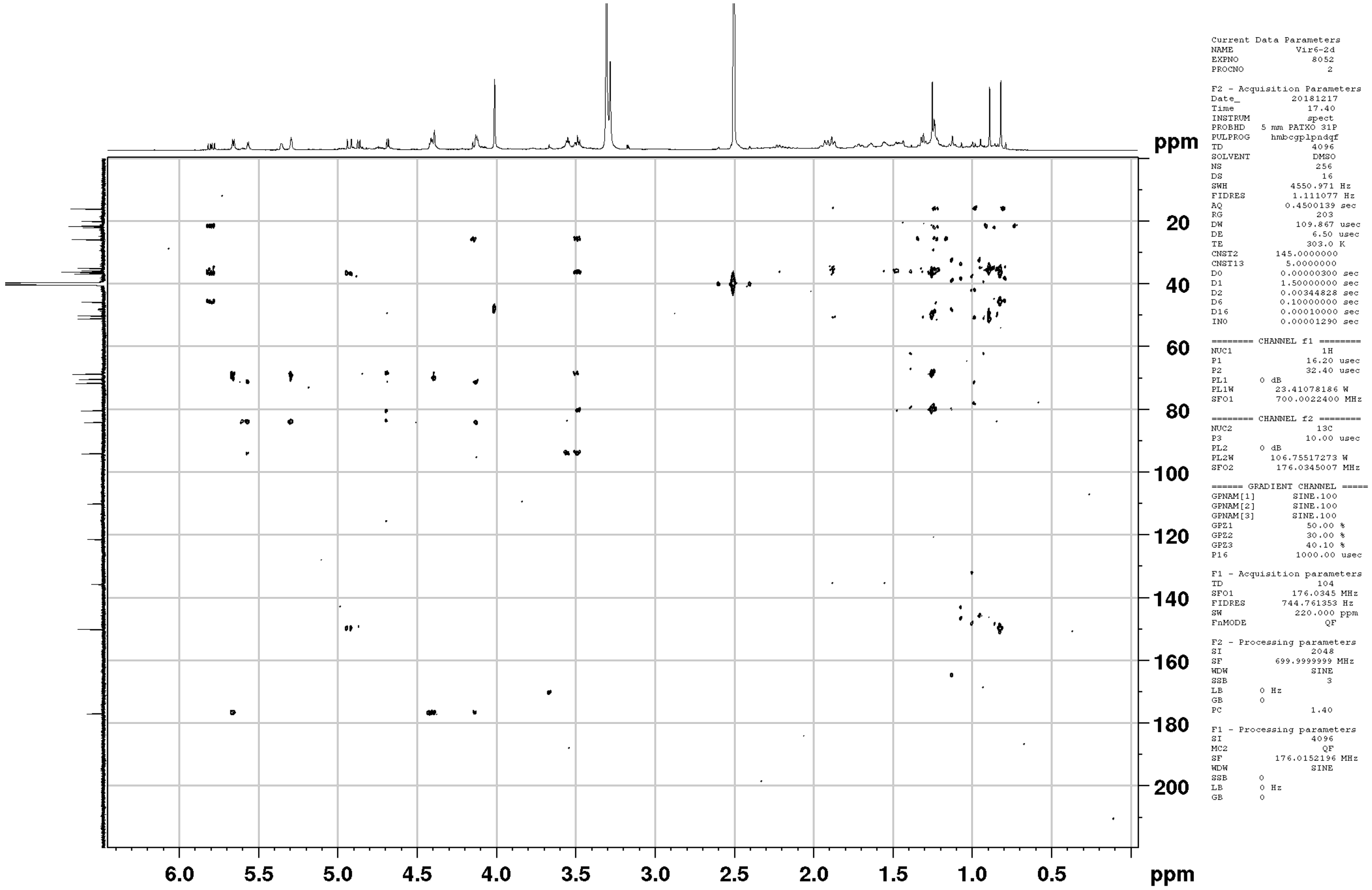

Figure S68. HMBC spectrum (700 MHz, DMSO-d_6) of **13**

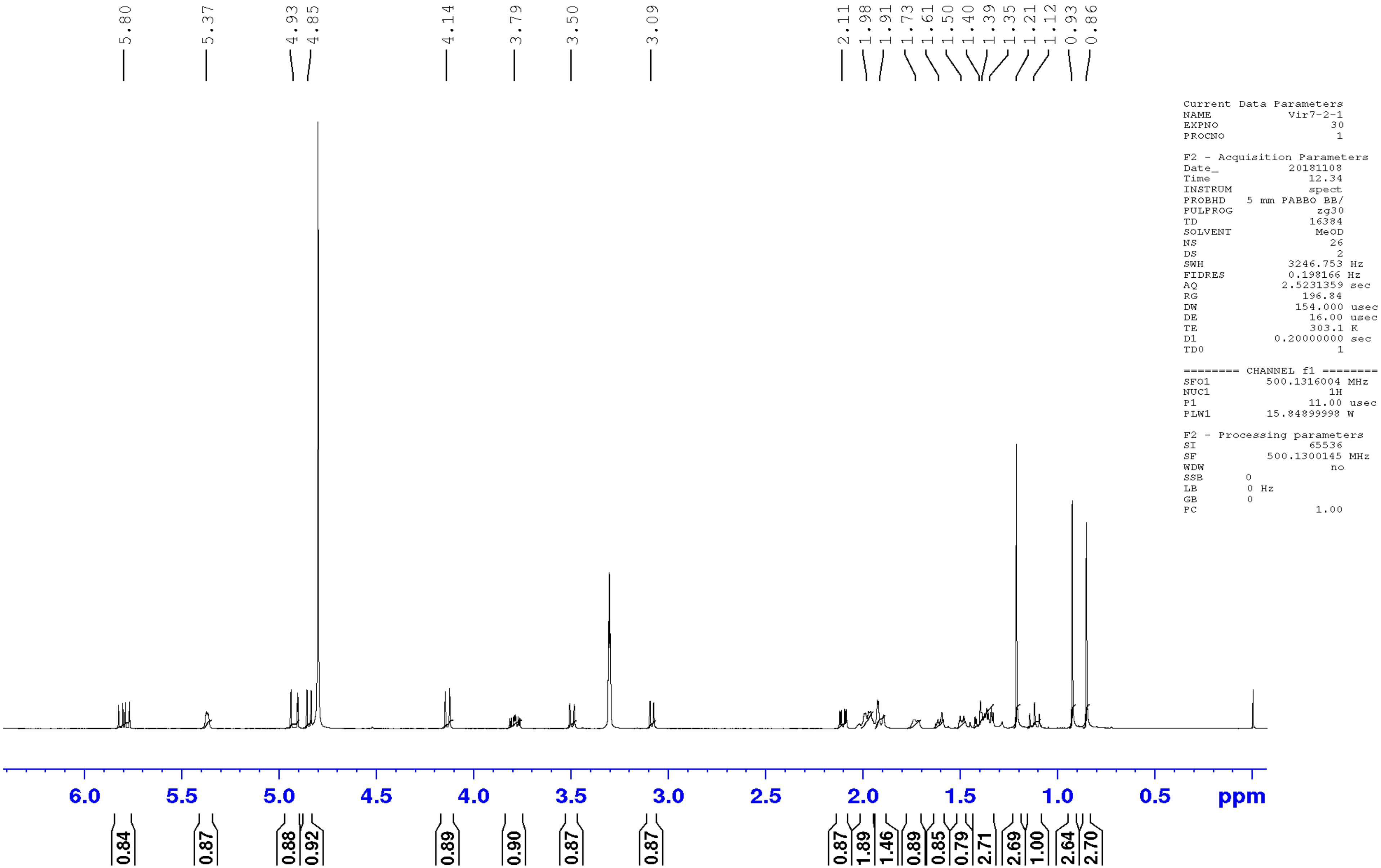

Figure S69. ^{1}H NMR spectrum (500 MHz, CD_3OD) of **14**

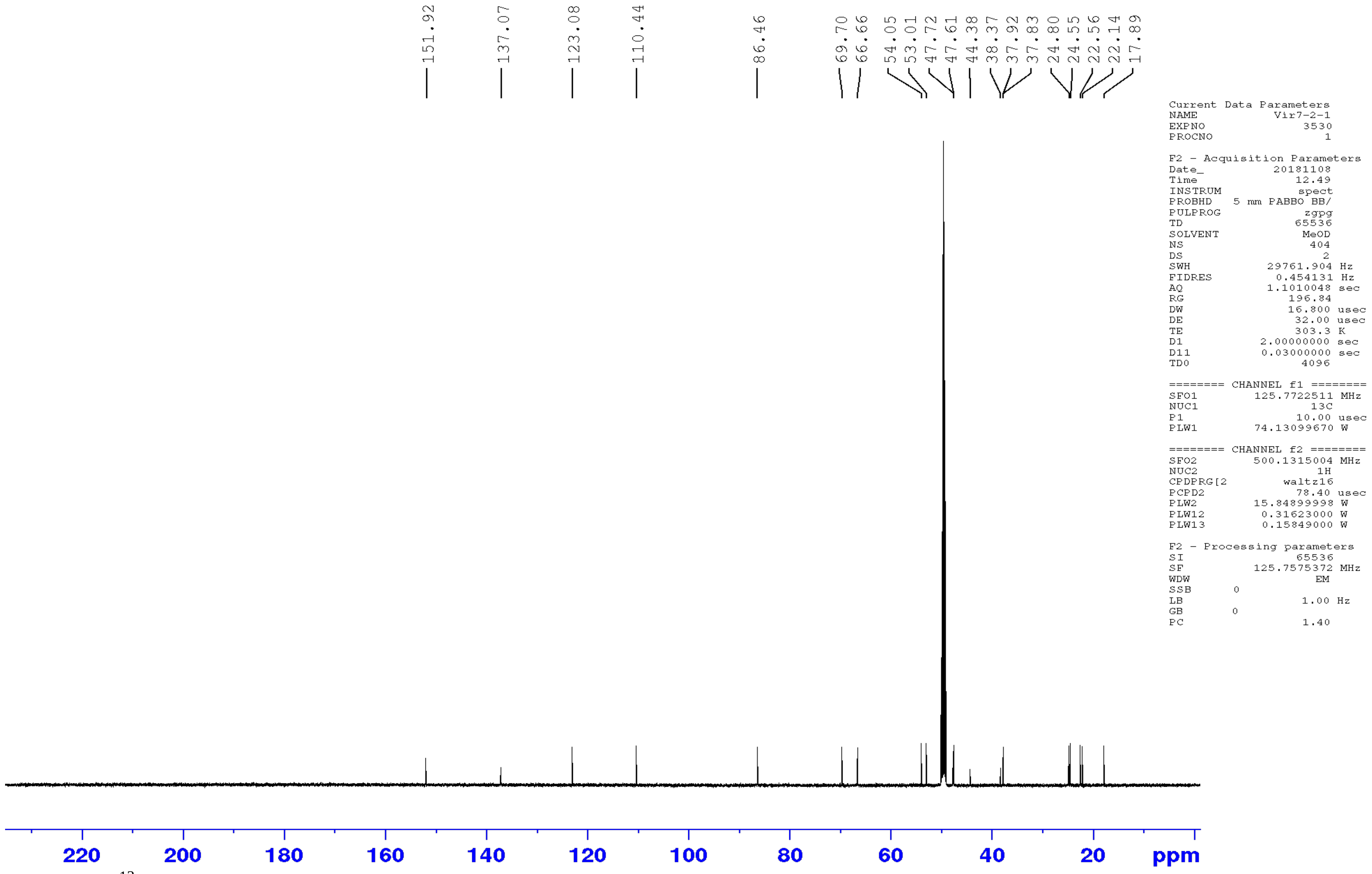

Figure S70. ^{13}C NMR spectrum (125 MHz, CD_3OD) of **14**

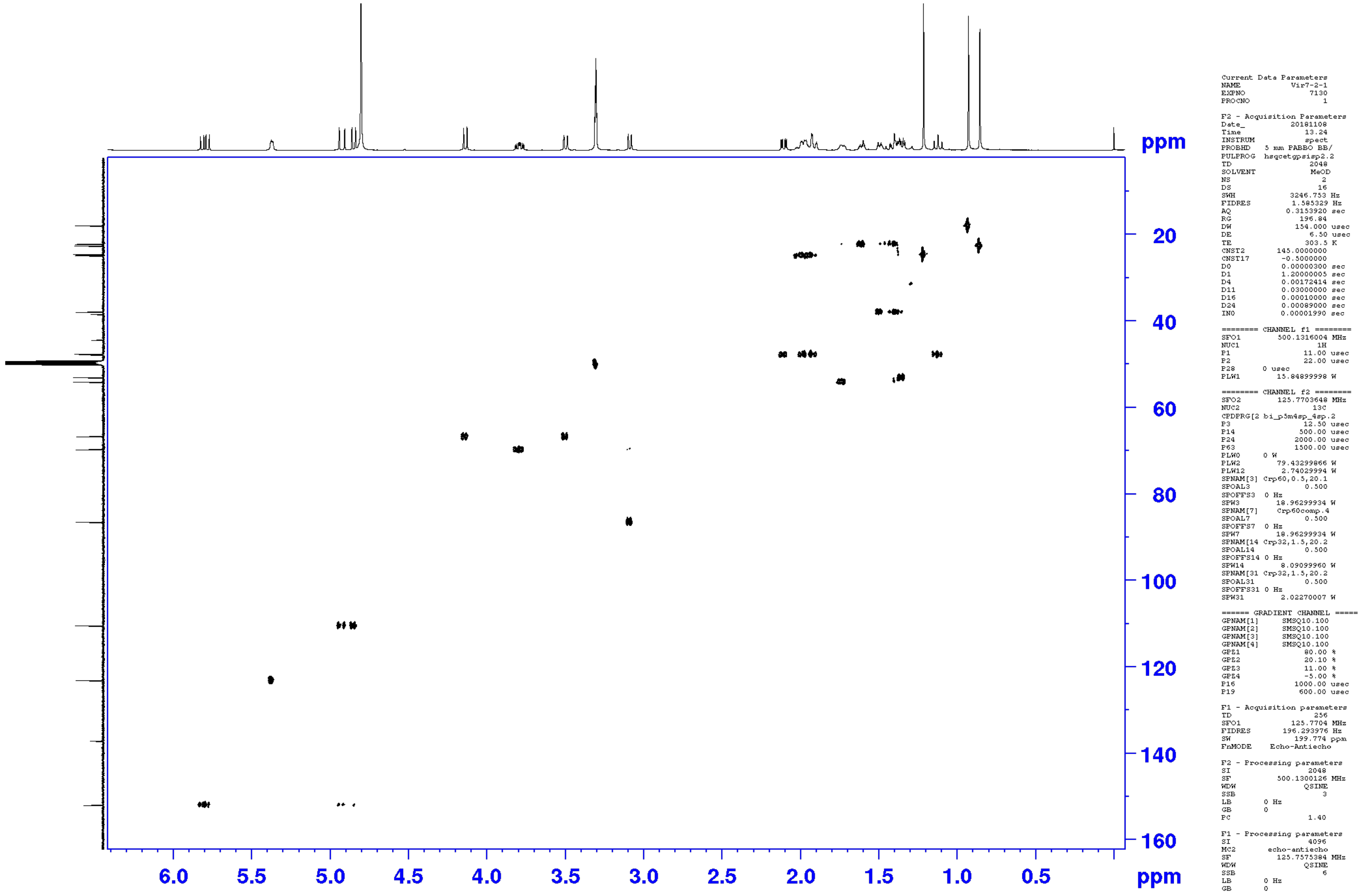

Figure S71. HSQC spectrum (500 MHz, CD_3OD) of **14**

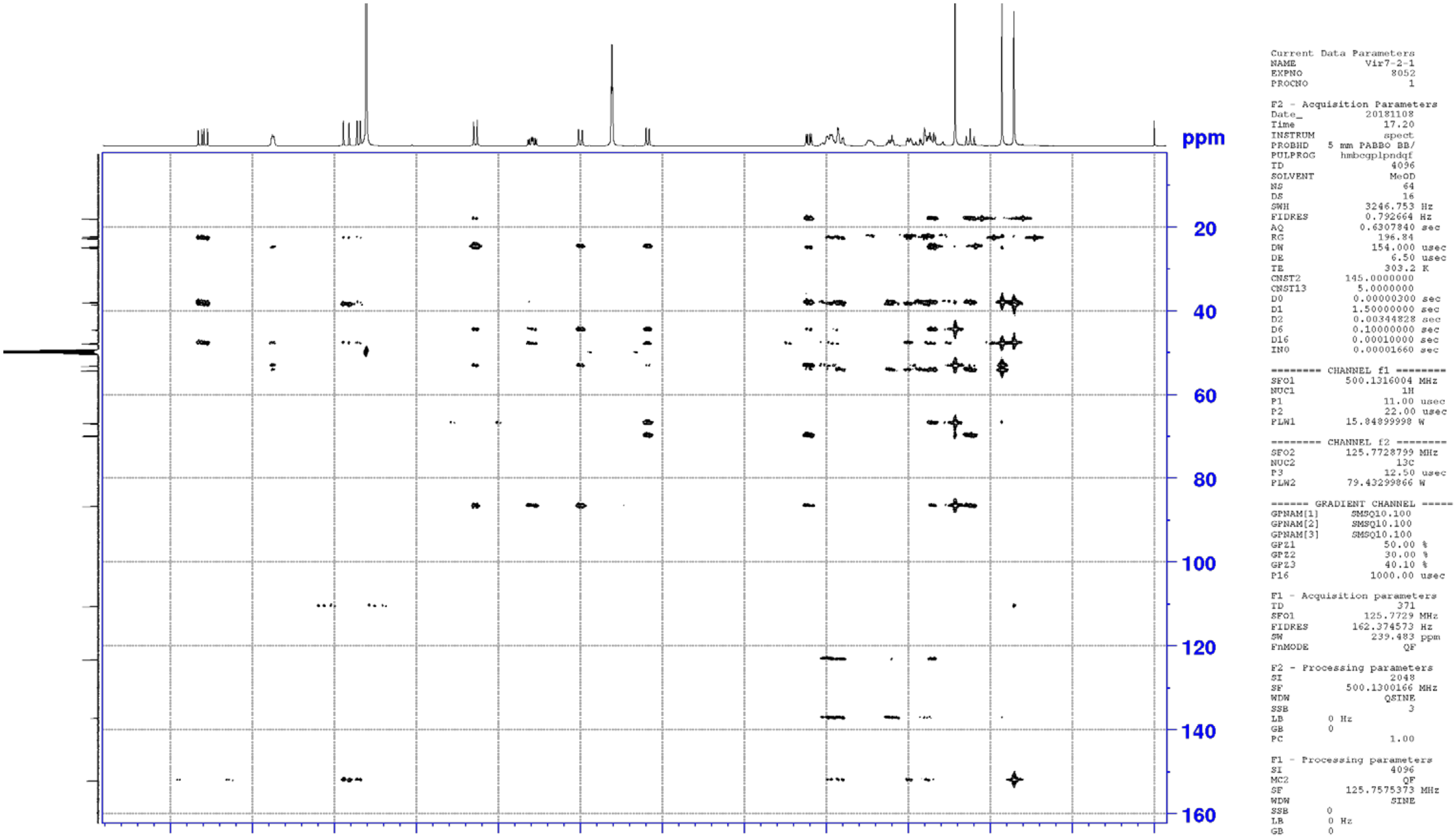

Figure S72. HMBC spectrum (500 MHz, CD_3OD) of **14**

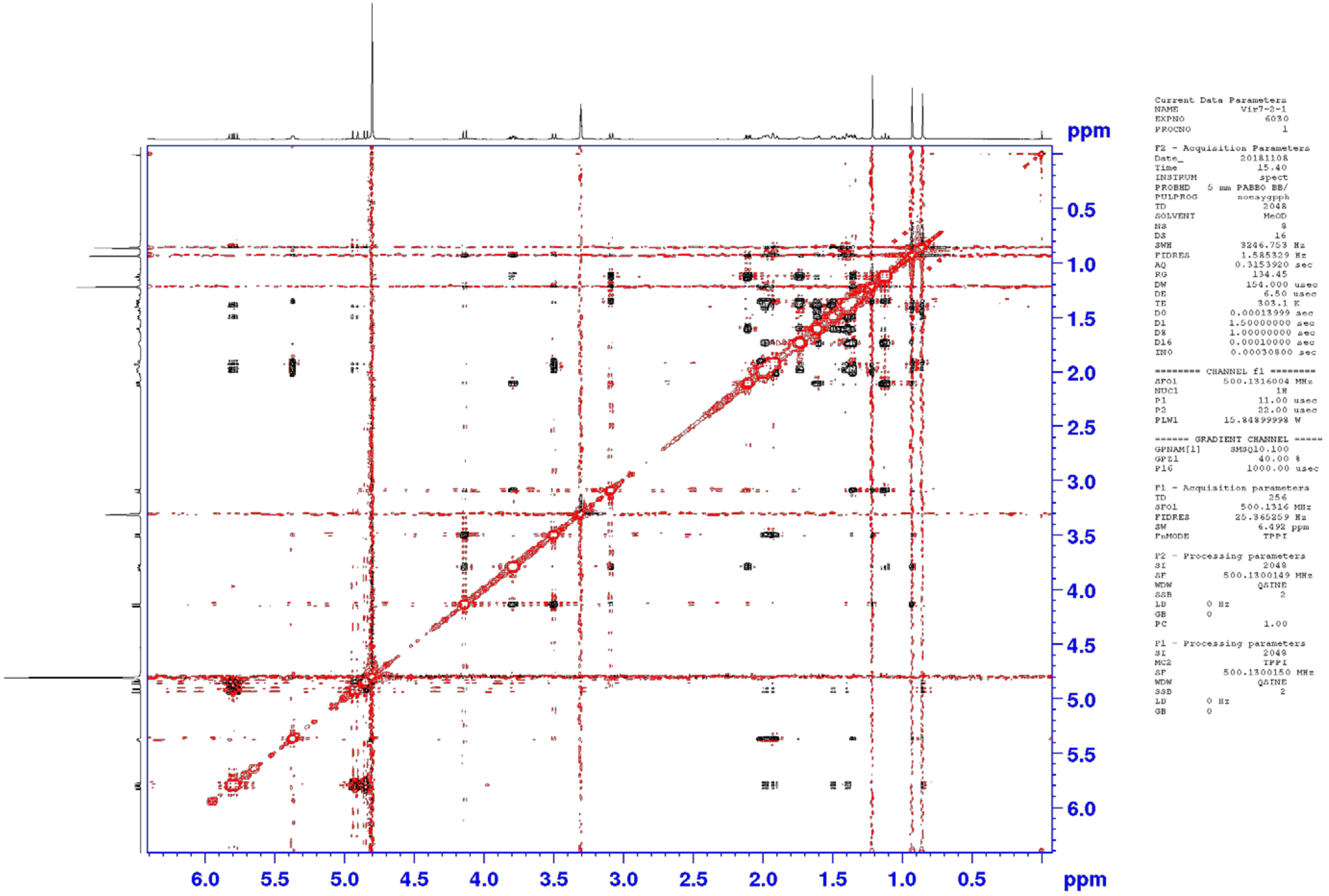

Figure S73. NOESY spectrum (500 MHz, CD_3OD) of **14**
